# Association studies of dopamine synthesis and metabolism genes with multiple phenotypes of heroin dependence

**DOI:** 10.1186/s12881-020-01092-0

**Published:** 2020-07-31

**Authors:** Yunxiao Li, Yongsheng Zhu, Jianghua Lai, Xugang Shi, Yuanyuan Chen, Jinyu Zhang, Shuguang Wei

**Affiliations:** 1 College of Forensic Science, School of Medicine, Xi’an Jiaotong University, Xi’an, 710061 Shaanxi China; 2 Key Laboratory of Environment and Genes Related to Diseases, Ministry of Education, Xi’an, 710061 Shaanxi China

**Keywords:** Dopamine, Heroin dependence, Phenotype, Memory

## Abstract

**Background:**

Heroin dependence is a complex disease with multiple phenotypes. Classification of heroin users into more homogeneous subgroups on the basis of these phenotypes could help to identify the involved genetic factors and precise treatments. This study aimed to identify the association between genetic polymorphisms of DA synthesis and metabolism genes, including tyrosine hydroxylase (*TH*), DOPA decarboxylase (*DDC)*, solute carrier family 6 member 3 (*SLC6A3*) and DA beta-hydroxylase (*DBH*), with six important phenotypes of heroin dependence.

**Methods:**

A total of 801 heroin dependent patients were recruited and fourteen potential functional single nucleotide polymorphisms (SNPs) were genotyped by SNaPshot. Associations between SNPs with six phenotypes were mainly assessed by binary logistic regression. Generalized multifactor dimensionality reduction was used to analyze the gene-by-gene and gene-by-environment interactions.

**Results:**

We found that *DBH* rs1611114 TT genotype had a protective effect on memory impairment after heroin dependence (*P* = 0.002, OR = 0.610). We also found that the income-rs12666409-rs129915-rs1611114 interaction yielded the highest testing balance accuracy and cross-validation consistency for memory change after heroin dependence.

**Conclusions:**

Our results suggest that the memory change after heroin dependence was a result of a combination of genetics and environment. This finding could lead to a better understanding of heroin dependence and further improve personalized treatment.

## Background

Drug dependence is a chronic relapsing brain disease that results from a combination of genetic and environmental factors [1], and the genetic factor accounts for approximately 30–60% of the total risk [2, 3]. Although multiple genes for the susceptibility to heroin dependence have been identified, a lot of these susceptibility genes could not be confirmed in subsequent studies, which further reminded us of the complexity of the genetic mechanism of heroin dependence. Heroin dependence is a complex disease with multiple phenotypes [4]. Classification of heroin users into more homogeneous subgroups on the basis of clinical and/or pathophysiological features could help to identify the involved genetic factors and precise treatments [4]. A previous study showed no significant association between the dopamine (DA) receptor D1 gene and opioid dependence in the overall analysis, but the association became apparent after stratifying based on the duration of the transition from first use to dependence (DTFUD) [5]. The DTFUD determined the addictive liability of the dependent patients [5, 6]. Early opioid use was associated with higher rates of impulsivity and relapse of drug seeking behaviour [7, 8]. Individuals have different tolerances to heroin [9] or methadone [10] in terms of the amount required to achieve the same efficacy. Therefore, individuals require different doses of heroin or methadone. Heroin dependence leads to pathological memory formation and seriously affects normal working memory [11]. These phenotypes of heroin dependence vary widely among heroin dependent individuals and may be affected by genetic factors.

DA system modulates motivation, cognition and reward in the context of dependence [12–14]. Extracellular DA concentrations are the net result of release (exocytosis) and clearance (uptake) from the extracellular space. Tyrosine hydroxylase (TH) converts L-tyrosine to L-DOPA, and then DOPA decarboxylase (DDC) catalyzes the decarboxylation of L-DOPA to generate DA [15]. The solute carrier family 6 member 3 (SLC6A3) encodes a DA transporter (DAT) that can re-uptake DA released into the synaptic cleft to the presynaptic terminal [16]. The DA beta-hydroxylase (DBH) protein catalyses the oxidative hydroxylation of DA to norepinephrine [17]. These factors regulate extracellular DA concentrations at different levels.

The A allele of *SLC6A3* rs27072 was associated with an early-onset age of nicotine dependence [18]. The frequency of the G allele of *TH* rs6356 was significantly lower in the late-onset Parkinson’s disease group [19]. Male ever-smokers with the AA genotype of *DBH* rs5320 smoked fewer cigarettes per day [20]. Haplotype-based association analysis revealed a protective T-G-T-G haplotype of *DDC* rs921451-rs3735273-rs1451371-rs2060762, which was significantly associated with nicotine dependence after correction for multiple testing [21]. However, the association of these genes with other important phenotypes of heroin dependence remained unclear.

We selected six phenotypes: memory change after heroin dependence, age of onset for heroin use, euphoria associated with first heroin use, DTFUD, daily heroin dose and daily methadone dose. We identified 14 potential functional single nucleotide polymorphisms (SNPs) by bioinformatics. We then analyzed the associations between the SNPs located in *SLC6A3* (rs10064525, rs27072, rs1042098 and rs6347), *TH* (rs10770140, rs10770141, rs3842727 and rs6356), *DDC* (rs11575553 and rs12666409) and *DBH* (rs129882, rs129915, rs1611114 and rs5320) with these phenotypes. We performed survival analysis for DTFUD. Finally, we analyzed the gene-by-gene and gene-by-environment interactions in these significant phenotypes.

## Methods

### Subjects

A total of 801 heroin-addicted individuals (mean age 39.30 ± 8.291 years) participated in our study. All of these participants were Chinese Han population. They were all unrelated individuals that were neither first-degree relatives, second-degree relatives nor third-degree relatives. This study included 709 males and 92 females. These participants were recruited from Lantian Compulsory Isolated and Detoxification Center, Xi’an Methadone Maintenance Treatment (MMT) Center and Xin’an Central Hospital Drug Rehabilitation Center. Heroin dependence was evaluated according to the Diagnostic and Statistical Manual of Mental Disorders fifth edition (DSM-V) criteria and a urine test. A structured interview was performed to assist with the diagnosis and to ask questions regarding (1) basic information: age, gender, nationality, education level, employment status, individual income, marital status and family atmosphere; (2) heroin use: the age of onset for heroin use, DTFUD, the daily dose of heroin used, reasons for first use of heroin, the administration routes of heroin, the euphoria associated with the first heroin use and the euphoria associated with heroin use after dependence; (3) individual responses after heroin use: body weight, sleep quality, memory, appetite and sexual desire; (4) methadone maintenance therapy or withdrawal symptoms: the number of withdrawal and relapse for heroin (absence of heroin use over 7 days counted as one withdrawal), the daily methadone dose used, and the methadone maintenance treatment duration. The detailed content of the questionnaire was shown in Supplementary file 1. Exclusion criteria were as follows: age < 18 years old; dependence on other substances; presence of other neuropsychiatric diseases; and involvement in other clinical trials. A total of 737 (92%) subjects were tobacco smokers in this study. All smokers consumed< 20 cigarettes per day and did not meet the criteria for nicotine dependence according to the Fagerström Test for Nicotine Dependence (FTND, score of 4 or lower defined as no dependence).

Written informed consent was obtained from all the study participants. The Ethical Committee of Xi’an Mental Health Center approved our study. We followed the approved guidelines to perform the protocol.

### SNP selection

SNP selection criteria were as follows: (1) located in the promoter, intron-exon border, exon, the 3’near region and untranslated regions (UTRs) of gene; (2) the minor allele frequency (MAF) greater than 0.05 based on dbSNP Han Chinese Beijing (HCB) and HapMap databases; (3) each SNP had three genotypes. Then 5 SNPs in *SLC6A3* (rs10064525, rs27072, rs1042098 and rs6347), 4 SNPs in *TH* (rs10770140, rs10770141, rs3842727 and rs6356), 2 SNPs in *DDC* (rs11575553 and rs12666409) and 4 SNPs in *DBH* (rs129882, rs129915, rs1611114 and rs5320) were selected. Some important information of these SNPs was summarized in Table 1. Potential functional effects of these SNPs were predicted by SNP Function Prediction (FuncPred, https://snpinfo.niehs.nih.gov/snpinfo/snpfunc.html). Detailed effects of these SNPs were shown in Table S1 of Supplementary file 2.

**Table 1:** The information of selected SNPs

Gene	Variable/ID	Allele	Gene location	Position (GRCh38.p7)	MAF
*SLC6A3*	rs10064525	G	3’near	5:1392606	0.0921
		T			
	rs27072	T	3’UTR	5:1394407	0.2051
		C			
	rs1042098	G	3’UTR	5:1394700	0.2951
		A			
	rs6347	C	Exon	5:1411297	0.2985
		T			
*TH*	rs10770140	C	5’near	11:2172367	0.3197
		T			
	rs10770141	A	5’near	11:2172610	0.3413
		G			
	rs3842727	G	3’near	11:2163618	0.3576
		T			
	rs6356	T	Exon	11:2169721	0.4305
		C			
*DDC*	rs11575553	A	3’UTR	7:50458521	0.0769
		G			
	rs12666409	A	5’near	7:50567279	0.2654
		T			
*DBH*	rs129882	T	3’UTR	9:133658547	0.2554
		C			
	rs129915	G	3’near	9:133659796	0.2698
		A			
	rs1611114	T	5’near	9:133635081	0.4101
		C			
	rs5320	A	Exon	9:133642351	0.1018
		G			

### Genotyping

The genomic DNA was extracted by E.Z.N.A™ Blood DNA Midi Kit (Omega Bio-TeK, Norcross, GA, USA). Then, we used SNaPshot technology to genotype these SNPs. These specific experimental steps can be found in our previous research [22].

### Statistical analysis

Hierarchical data on phenotype variables were used to identify subtypes, and associations between these subtypes with SNPs were first assessed by Pearson’s chi-square test or Fisher’s exact test. Then, these associations were assessed again by binary logistic regression, and the basic information (age, gender, education level, employment status, individual income, marital status and family atmosphere) were set as covariates.

We assessed the potential correlation of DTFUD with these genotypes using Kaplan–Meier survival analysis. Survival curves were used to estimate the probability that individuals had not experienced dependence over a period of time following their first heroin use. The DTFUD was regarded as survival time in months. The origin of the first use was specified as the time of first heroin use, and the outcome of interest was the first occurrence of dependence. We used three methods (Wilcoxon, log rank and Tarone–Ware tests) to compare the survival curves, and these methods give varying weight to different phases of follow-up time.

We used generalized multifactor dimensionality reduction (GMDR; http://sourceforge.net/projects/gmdr/) to analyze the gene-by-gene and gene-by-environment interactions. This method permits adjustment for discrete and quantitative covariates and is applicable to both dichotomous and continuous phenotypes in various population-based study designs [23]. The multi-locus model with the maximum testing balance accuracy (Testing Bal. Acc) and cross-validation consistency (CV Consistency) was the best interaction model.

*P* values were calculated based on our previous research [22] *P* values were presented as two-sided, and the statistically significant was defined as *P* < 0.05. We used Bonferroni’s correction to adjust the significance level, and the *P* value was divided by the total number of loci analyzed (15). Consequently the loci should be considered significant at *P* < 0.003. All statistical analyses were performed using SPSS 20.0 software (SPSS, USA).

## Results

### Association of *DBH* with memory change after dependence

For the phenotype of memory, a total of 586 subjects (73.2%) that memory impaired were assigned to the “reduction” group, and 215 subjects (26.8%) that memory no changed were assigned to the “no difference” group.

The frequency of *DBH* rs1611114 TT genotype was significantly lower in the reduction group (*P* = 0.002, OR = 0.610, 95%CI = 0.445–0.835, Table 2). This association was still significantly after adjustment of covariates (*P* = 0.002, OR = 0.607, 95%CI = 0.440–0.837, Table 2). These associations of other SNPs with memory change after dependence were listed in Table S2 of Supplementary file 2.

**Table 2:** The association between SNP and memory change after dependence

Gene	Variable/ID	No change (*n* = 215)		Reduced (*n* = 586)		*P*^a^	OR, 95% CI	*P*^b^	Exp(B), 95% CI
		Number	Percent	Number	Percent				
	rs1611114					**0.007**^**n**^		**0.005**^**n**^	
	TT	111	51.6	231	39.4	**0.002**	0.610, 0.445–0.835	**0.002**	0.607, 0.440–0.837
	TC	79	36.7	281	48.0	**0.005**^**n**^	1.586, 1.150–2.187	**0.002**	1.673, 1.204–2.325
	CC	25	11.6	74	12.6	0.703	1.098, 0.678–1.780	0.956	0.986, 0.600–1.620
	T	301	70.0	743	63.4	**0.014**^**n**^	0.742, 0.585–0.942		
	C	129	30.0	429	36.6				

*P*^a^: Genotypic and allele frequency difference assessment by chi-square test

*P*^b^: Genotypic and allele frequency difference assessment by binary logistic regression

^n^: The result was not significant after Bonferroni’s correction

### SNPs and the age of onset for heroin use

The age of onset for heroin use was converted to binary using median (28 years old), the sample was divided into earlier-onset (*n* = 440, 54.9%) and later-onset (*n* = 361, 45.1%) groups accordingly.

After Bonferroni’s correction, *TH* rs10770140 and rs10770141 were not associated with the age of onset for heroin use (*P* = 0.018 and *P* = 0.015, respectively, Table 3). These patients who carrying the rs10770140 TT genotype (*P* = 0.015, OR = 0.599, 95%CI = 0.395–0.908, Table 3) or the T allele (*P* = 0.013, OR = 0.604, 95%CI = 0.405–0.901, Table 3) were later-onset, but these findings were not observed after Bonferroni’s correction. These patients who carrying the rs10770141 GG genotype (*P* = 0.012, OR = 0.577, 95%CI = 0.374–0.890, Table 3) or the G allele (*P* = 0.010, OR = 0.580, 95%CI = 0.382–0.881, Table 3) were later-onset, but these findings were not observed after Bonferroni’s correction.

**Table 3:** The associations between SNPs and age of onset for heroin use

Gene	Variable/ID	Age ≤ 28 (*n* = 440)		Age > 28 (*n* = 361)		*P*^a^	OR, 95% CI	*P*^b^	Exp(B), 95% CI
		Number	Percent	Number	Percent				
*TH*	rs10770140					**0.018**^**n**^		0.092	
	TT	366	83.2	322	89.2	**0.015**^**n**^	0.599, 0.395–0.908	**0.019**^**n**^	0.568, 0.354–0.912
	TC	72	16.4	39	10.8	0.023	1.615, 1.064–2.452	0.032	1.681, 1.047–2.703
	CC	2	0.5	0	0.0	0.504	1.005, 0.998–1.011	0.999	0.000, 0.000-
	T	804	91.4	683	94.6	**0.013**^**n**^	0.604, 0.405–0.901		
	C	76	8.6	39	5.4				
	rs10770141					**0.015**^**n**^		0.071	
	GG	371	84.3	326	90.3	**0.012**^**n**^	0.577, 0.374–0.890	**0.014**^**n**^	0.538, 0.328–0.881
	GA	67	15.2	35	9.7	0.019	1.673, 1.083–2.585	0.024	1.77, 1.080–2.907
	AA	2	0.5	0	0.0	0.504	1.005, 0.998–1.001	0.999	0.000, 0.000-
	G	809	91.9	687	95.2	**0.010**^**n**^	0.580, 0.382–0.881		
	A	71	8.1	35	4.8				

*P*^a^: Genotypic and allele frequency difference assessment by chi-square test

*P*^b^: Genotypic and allele frequency difference assessment by binary logistic regression

^n^: The result was not significant after Bonferroni’s correction

After Bonferroni’s correction, these associations of rs10770140 TT (*P* = 0.019, OR = 0.568, 95%CI = 0.354–0.912, Table 3) and rs10770141 GG (*P* = 0.014, OR = 0.538, 95%CI = 0.328–0.881, Table 3) genotypes with age of onset were still not significant after adjustment of covariates. These associations of other SNPs with age of onset for heroin use were listed in Table S3 of Supplementary file 2.

### SNPs and the euphoria of the first heroin use

For the phenotype of euphoria, a total of 173 subjects (21.6%) that have obvious feelings of euphoria were assigned to the “strong euphoria” group, and 628 subjects (78.4%) that have minimal level of happiness, no euphoria or unclear were assigned to the “week euphoria” group.

After Bonferroni’s correction, *TH* rs10770140 (*P* = 0.047, Table 4) and rs10770141 (*P* = 0.035, Table 4) were not associated with the intensity of euphoria. The subjects carrying the rs10770140 CC (*P* = 0.046, OR = 1.012, 95%CI = 0.996–1.028, Table 4) or rs10770141 AA (*P* = 0.046, OR = 1.012, 95%CI = 0.996–1.028, Table 4) genotype had a stronger euphoric response to heroin, but this effect was not observed after Bonferroni’s correction. These associations were not significant after adjustment for the covariates. These associations of other SNPs with the intensity of euphoria were listed in Table S4 of Supplementary file 2.

**Table 4:** The associations between SNPs and euphoria associated with the first heroin use

Gene	Variable/ID	Weak(*n* = 628)		Strong (*n* = 173)		*P*^a^	OR, 95% CI	*P*^b^	Exp(B), 95% CI
		Number	Percent	Number	Percent				
*TH*	rs10770140					**0.047**^**n**^		0.791	
	TT	538	85.7	150	86.7	0.729	1.091, 0.667–1.785	0.741	1.088, 0.659–1.798
	TC	90	7.2	21	12.1	0.460	1.211,0.728–2.012	0.460	1.215, 0.724–2.037
	CC	0	0.0	2	1.2	**0.046**^**n**^	1.012, 0.996–1.028	0.999	7,208,139,313, 0.000-
	T	1166	92.8	321	92.8	0.970	1.009, 0.637–1.598		
	C	90	7.2	25	7.2				
	rs10770141					**0.035**^**n**^		0.593	
	GG	544	86.6	153	88.4	0.529	1.181, 0.703–1.986	0.515	1.192, 0.702–2.025
	GA	84	6.7	18	10.4	0.299	1.329,0.775–2.278	0.283	1.351,0.780–2.342
	AA	0	0.0	2	1.2	**0.046**^**n**^	1.012, 0.996–1.028	0.999	7,208,139,313, 0.000-
	G	1172	93.3	324	93.6	0.827	0.947, 0.583–1.539		
	A	84	6.7	22	6.4				

*P*^a^: Genotypic and allele frequency difference assessment by chi-square test

*P*^b^: Genotypic and allele frequency difference assessment by binary logistic regression

^n^: The result was not significant after Bonferroni’s correction

### SNPs and the daily dose of methadone or heroin

For the phenotype of methadone dose, the sample was divided into a high methadone dose group (*n* = 285, 44.9%) and a low methadone dose group (*n* = 350, 55.1%) based on the median daily methadone dose (45 mg). For the phenotype of heroin dose, the sample was divided into a high heroin dose group (*n* = 375, 46.8%) and a low heroin dose group (*n* = 426, 53.2%) based on the median daily heroin dose (0.5 g). No SNP was associated with the daily dose of methadone or heroin use (Table S5 and S6 of Supplementary file 2, respectively).

### SNPs and DTFUD

For the phenotype of DTFUD, the sample was divided into a long duration group (*n* = 376, 46.9%) and a short duration group (*n* = 425, 53.1%) based on the median of DTFUD (1 month). No SNP was associated with DTFUD in either Pearson’s chi-square test (Table S7 of Supplementary file 2) or the Kaplan–Meier survival analysis (Fig. S1 of Supplementary file 3).

### Gene-by-gene and gene-by-environment interactions

The one- to four-way gene-by-gene and gene-by-environment interaction models of the memory change after dependence were analyzed by GMDR.

For the phenotype of memory change after dependence, we found that these interactions in the one- to four-way models were significant. In two-way model (income-rs3842727), income and rs3842727 have interaction. In three-way model (income-rs129915-rs1611114), income, rs129915 and rs1611114 have interaction. In four-way model (income-rs12666409-rs129915-rs1611114), income, rs12666409, rs129915 and rs1611114 have interaction. Among these significant interactions, the CV Consistency and Testing Bal. Acc in the four-way model (income-rs12666409-rs129915-rs1611114) were the most significant, this model was the best model (Table 5).

**Table 5:** Gene-by-gene and gene-by-environment interactions in phenotype of memory change after dependence

Model	Training Bal. Acc	Testing Bal. Acc	CV Consistency	Sign Test (*P*)
Income	0.5872	0.5900	10/10	10 (**0.0010**)
Income rs3842727	0.5949	0.5651	4/10	10 (**0.0010**)
Income rs129915 rs1611114	0.6280	0.5804	9/10	9 (**0.0107**)
Income rs12666409 rs129915 rs1611114	0.6724	0.5946	10/10	9 (**0.0107**)

These sequencing data of SNPs were listed in Supplementary file 4.

## Discussion

Heroin dependence is a heterogeneous disease, and its clinical features and therapeutic outcomes vary substantially among patients. Regardless of linkage analysis or associative analysis, the results are obviously affected by disease grouping within samples, which finally result in poor stability. Features that correlated with dependence could be used as indicators to categorize heroin dependent patients into subgroups with uniform disease features.

In this study, we demonstrated that *DBH* rs1611114 was significantly associated with memory change after dependence. SNP rs1611114 was located in the 5’near region of *DBH* and was predicted to be located in the transcription factor binding site (Table S8 of Supplementary file 2). Research found that *DBH* − 1021C/T variant accounted for 35–52% of the variation in plasma DBH activity, and the T allele predicted very low DBH activity [24]. Studies have also found that lower DBH expression levels lead to higher levels of extracellular DA and therefore have better working memory [25, 26]. Hippocampal cannabinoid receptor 1, found exclusively in DBH-expressing cells, was both necessary and sufficient to impair memory consolidation produced by acute stress [27]. DBH also modulated working memory and verbal and visual memory and sustained attention during the cannabis intoxication period [28]. These studies shown that rs1611114 may affect memory phenotype by regulating DBH expression. Similar to these findings, we found that the frequency of the rs1611114 TT in the memory reduction group was significantly lower, which indicated that subjects in the memory reduction group may have lower levels of extracellular DA and therefore have worse memory. Furthermore, income and *TH* rs3842727 exhibited interactions in the memory change phenotype. Income, *DBH* rs129915, rs1611114 exhibited interactions in this phenotype. Rs1611114, *DDC* rs1266640, *DBH* rs129915 and income also exhibited interactions in this phenotype. These results showed that the memory change phenotype was a result of a combination of genetics and environment. TH converts L-tyrosine to L-DOPA, DDC catalyzes the decarboxylation of L-DOPA to generate DA, and then DBH catalyses the oxidative hydroxylation of DA to norepinephrine. SNPs in these genes may interact with each other in regulating the concentration of DA, and then affected the memory change after heroin dependence. Research showed that the higher-income group had greater memory capacity [29]. Children from economically disadvantaged background showed lower memory performance and had a smaller hippocampal volume [30]. Lower income was associated with a flatter cortisol awakening response, blunted reactivity and recovery to acute stress [31]. Therefore, higher income may have a protective effect on memory impairment.

*TH* rs10770140 and rs10770141 were promoter variants that differentially affected the transcriptional activity of TH [32] (Table S9 and Table S10 of Supplementary file 2, respectively). Under basal circumstances and nicotine stimulation circumstances, the rs10770141 A and rs10770140 C alleles exerted a greater effect on TH transcription [32, 33]. However, after Bonferroni’s correction, these associations of rs10770140 C and rs10770141 A alleles with age of onset for heroin use were not significant. These associations of rs10770140 CC and rs10770141 AA genotypes with the intensity of euphoria were still not significant after Bonferroni’s correction. The *TH* Val-81-Met polymorphism was found to be associated with early-onset alcoholism [34], as the *P* value of this article was not corrected.

Some studies have found that *SLC6A3* rs27072 may be related to the age of onset for tobacco or alcohol [35–37]. In particular, rs27072-A allele carriers were more likely to initiate smoking onset before 18 years old [36]. However, our research did not find that *SLC6A3* rs27072 was related to the age of onset for heroin use. We proposed three hypotheses to explain the difference. First, our study excluded alcohol and nicotine dependent subjects. Second, the proportion of our sample that use heroin before 18 years old was relatively small (Fig. S2 of Supplementary file 3), and age of onset in our study was converted to binary using median (28 years old). Third, the mechanism of heroin dependence may different from that of alcohol and nicotine dependence.

## Conclusions

In conclusion, our results supported the important role of *DBH* rs1611114 in memory change of heroin dependence. This phenotype was a result of a combination of genetics and environment. These findings were valuable for obtaining a better understanding of heroin dependence and improving personalized treatment.

## Supplementary information

**Additional file 1:.** Supplementary file 1: Questionnaire.

**Additional file 2: **Supplementary file 2: **Table S1** The functional effects of all selected SNPs. **Table S2** Results of association analysis between other SNPs and phenotype of memory. **Table S3** Results of association analysis between other SNPs and age of onset for heroin use. **Table S4** Results of association analysis between other SNPs and phenotype of euphoria. **Table S5** Results of association analysis between SNPs and phenotype of methadone dosage. **Table S6** Results of association analysis between SNPs and phenotype of heroin dosage. **Table S7** Results of association analysis between SNPs and phenotype of DTFUD. **Table S8** Transcription factor binding sites of rs1611114. **Table S9** Transcription factor binding sites of rs10770140. **Table S10** Transcription factor binding sites of rs10770141.

**Additional file 3:.** Supplementary file 3: Figure. S1. Kaplan–Meier survival analysis of DTFUD. Figure. S2. Frequency distribution of age of onset for heroin use.

**Additional file 4:.** Supplementary file 4: Sequence data.

## Data Availability

All the data supporting the conclusions of this article are presented within the manuscript or are available in the additional supporting file containing the supplementary material.

## Abbreviations

DA: Dopamine
DAT: DA transporter
DBH: DA beta-hydroxylase
DDC: DOPA decarboxylase
DSM-V: Diagnostic and Statistical Manual of Mental Disorders fifth edition
DTFUD: Duration of the transition from first use to dependence
FTND: Fagerström Test for Nicotine Dependence
GMDR: Generalized multifactor dimensionality reduction
HCB: Han Chinese Beijing
MAF: Minor allele frequency
MMT: Methadone Maintenance Treatment
SLC6A3: Solute carrier family 6 member 3
SNPs: Single nucleotide polymorphisms
TH: Tyrosine hydroxylase
UTRs: Untranslated regions
