# Supplementary material for: Association studies of dopamine synthesis and metabolism genes with multiple phenotypes of heroin dependence
Source: BMC Med Genet. 2020 Jul 31;21:157. doi: 10.1186/s12881-020-01092-0 (PMC7393710; doi:10.1186/s12881-020-01092-0)
Supplement: Supplementary file 1 — Additional file 1:. Supplementary file 1: Questionnaire. [file 12881_2020_1092_MOESM1_ESM.docx]

**Questionnaire**

Sample No.: ___________

【**Notes**】

Kindly remind you: To fill out the form as accurately as possible. Please write “do not know/unclear” if no relevant information for the question. Please tick(√) on the selected sequence number, or fill in the required information on the __________ .

1. **Basic Information**
2. **Gender:**

①Male; ②Female

1. **Age:**

______

1. **Nationality:**

①Han; ②Hui; ③Mongols; ④Tibetan; ⑤Uighur; ⑥Other______

1. **Education Level:**

①Primary or below;

②Junior high school;

③Technical secondary school;

④High school;

⑤Junior college;

⑥Bachelor degree or above

1. **Marital Status:**

①Unmarried;

②Cohabit;

③Married;

④Married but live separately;

⑤Divorced;

⑥Widowed

1. **Employment Status:**

①Unemployed;

②Worker;

③Farmer;

④Civil servant,public institution staff;

⑤Soldier, police;

⑥Student;

⑦Company employee;

⑧Entertainment employee;

⑨Service employee;

⑩Driver;

Individual household (the self-employed);

Others______

1. **Individual Income-Average Monthly(RMB/Yuan):**

①No income;

②1-500;

③501-1000;

④1001-2000;

⑤2001-3000;

⑥3001-5000;

⑦5001-10,000;

⑧10,000-30,000;

⑨more than 30,000

1. **Family atmosphere:**

①Harmonious;

②Not close;

③Terrible

**II. Drug Use**

1. The first time to abuse drugs when at ________ (age) / in the (year) of ______ .
2. The drugs abused are:
3. Heroin (please move to answer the third question);
4. Opium (please move to answer the third question) ;

③Amphetamine or methamphetamine (please move to answer the fourth question);

④Other drug______ (please move to answer the fourth question)

1. The duration from the first abuse until being addicted is:

①______ day (s); ②_____ month(s); ③______ year (s)

1. The heroin use for each time is ____gram (g), which is ____ Yuan per gram.
2. Heroin is used for ___ time(s) per day.
3. The maximum use of heroin is: ___ gram(g)for each time.
4. Before methadone maintenance treatment, the average dosage of heroin is ____ gram(g); ____ time(s) per day, and heroin has been used for ____ year(s).
5. The addictive substance once used is (Multiple choice):

①Heroin;②Opium;③Dolantin;④Morphine;⑤Dihydroetorphine(DHE);⑥Mathadone from black market;

⑦Buprenorphine;

⑧Tramadol;

⑨Sedative hypnotic drugs（E.g.: Diazepam);

⑩Amphetamines (E.g.: Methamphetamine, ecstasy, and Magu);

⑪Cannabis;

⑫Cocaine (Be attention, is not codeine);

⑬K powder (ketamine);

⑭Others ;

1. The reasons for the first use of heroin (Multiple choice):

①Imitation because of curiosity;

②Medical purpose;

③Amusement or in the pursuit of stimulation;

④Affected by friends or people around;

⑤Family influence;

⑥Relieving irritation or psychological pressure;

⑦Be persecuted or deceived;

⑧Anti fatigue and cheer up the spirit;

⑨Reduce weight;

⑩Social reasons;

Others

1. How to start drug use (Single choice)?

①Hot suction;

②Intravenous injection;

③Intramuscular injection;

④Nasal inhalation;

⑤Rolled with cigarette;

⑥Take orally;

⑦Take with wine;

⑧Take withsoft drink;

⑨Hot suction and intravenous injection;

⑩Others

1. The main drug abuse methods adopted later (multiple choice, but only the main ones):

①Hot suction;

②Intravenous injection;

③Intramuscular injection;

④Nasal inhalation;

⑤Rolled with cigarette;

⑥Take orally;

⑦Take with wine;

⑧Take withsoft drink;

⑨Hot suction and intravenous injection;

⑩Others

**III. Individual Responses after Heroin Use**

1. Euphoria for individual after the first use of heroin:

①No euphoria; ②Not too happy; ③Obvious feeling of euphoria;

④Strong euphoric feeling; ⑤Unclear; ⑥Others

1. Compared with the feelings before addiction, the euphoria after addiction are:

①No euphoria; ②Not too happy; ③Obvious feeling of euphoria;

④Strong euphoric feeling; ⑤Unclear; ⑥Others

3.Changes in sleep after addiction compared with those before addiction:

①No change in sleep;

②Poor sleep and frequent insomnia;

③Insomnia almost every time after use;

④Unclear

4.Changes for body weight after addiction compared with the weights before addiction:

①No change;

②Weight gain, about kg;

③Weight lose, about kg;

④Unclear

5. Changes for appetite after addiction compared to that before addiction:

①No change;

②Significant increase in appetite;

③Significantly decrease in appetite;

④Unclear

1. Changes in memory after addiction compared to that before addiction:

①No change;

②Impaired Memory;

③Significant impaired memory, forgetful;

④Unclear

1. Changes in sexual desire after addiction compared to that before addiction:

①No change;

②Obvious decrease;

③Disappear;

④Obviously enhanced;

⑤Unclear

**IV. Methadone Maintenance Therapy or withdrawal symptoms**

1. Up till now, trying drug withdrawal for___ (times) and drug relapse for___ (times).
2. The duration since the first time for coming to Drug Rehabilitation Center until now or the duration for receiving methadone maintenance treatment:

①____days;

②____months;

③____years;

1. The duration for coming to Addiction Treatment Centre this time or the duration for receiving methadone maintenance treatment:

①____days;

②____months

③____years

1. For receiving methadone maintenance treatment this time, the average dose of methadone taking for each time is ___mg. You take methadone in _____?(Which period of time in a day ) And take methadone once at the intervals of _____(days).
2. How long for receiving methadone maintenance treatment altogether?

①____days;

②____months

③____years

6.Urine test and heroin use within the recent one year after adopting methadone therapy:

①The urine test was negative, and stop heroin use;

②The urine test is positive, and heroin use is 1-4 times a month;

③The urine test is positive, and heroin use is 5-8 times a month;

④Urine test positive, the average monthly heroin use is more than 8 times;

⑤Unclear

7.When adopting methadone therapy, what are the reasons for keeping heroin use?

①Physical withdrawal symptoms;

②Psychological thirst;

③Pleasure seeking;

④Feel boring;

⑤Helpful for better sleeping;

⑥Influenced by friends surrounded;

⑦Bad mood

8. Improvements of social behaviors after methadone therapy:

①Returned to normal, social and family relations have improved significantly, fully being integrated into the society, restart to work, economically independent and relations with family members have been improvedsignificantly;

②Generally improved, social and family relations improved, partially being integrated into the society, no work, economically independent, and family relations have been generally improved;

③There is no change in social behaviors. Failure to be integrated into the society, no work, tension relationships with family members, and unhealthy social behaviors such as stealing, robbing, and cheating.

**V. Alcohol and Tobacco Abuse**

1. Smoking status before using heroin:

①Never smoke;

②Occasional smoking, no addiction;

③Having the habit of smoking;

④Used to smoke and abandoned smoking before start heroin use

1. Changes in smoking after addiction:

①No significant changes;

②Smoking heavily;

③Smoking lightly;

④Others

1. Alcohol drinking before the use of heroin:

①Never drink;

②Occasionally drink, no dependence;

③Regularly drink as a habit or alcohol dependence;

④Alcohol dependence before, stopped before using heroin

1. Changes in alcohol consumption after addiction:

①No significant changes;

②Significant increase;

③Significantly decrease;

④Others

**VI. Individual Health**

1. Have you ever had any serious diseases or have health problems (infectious diseases such as hepatitis B, hepatitis C, tuberculosis, and AIDS, diabetes, hypertension, coronary heart disease, etc.)?

①Yes; ②No; ③Unclear

2.For serious diseases or health problems, please describe the diseases and problems in details:

1. Name of the disease__________________;
2. Did you receive the treatment followed? ①Yes; ②No
3. Current situation_____________________:

①Obviously improved; ②No change or slight change; ③Continuous deterioration

3.Other mental illnesses accompanied:

①Yes, and write out the name of the disease_______________;

②No;

③Unclear

4.Temporal relation between mental illness and heroin addiction:

①This mental illness has existed prior to addiction;

②The disease or disorder occurs after heroin addiction;

③Unclear

5.Any treatments taken for this mental illness?

①Yes; ②No; ③Unclear;

6.Current status of the mental illness:

①Obviously improved; ②No change or slight change; ③Continuous deterioration

**VII. Family History**

1. What is the nationality of the biological father?

①the Han nationality; ②the Hui nationality; ③Other

2.What is the nationality of the biological mother?

①the Han nationality; ②the Hui nationality; ③Other

3.Family members (mainly parents) for having mental illness or any other serious diseases (hepatitis B, hepatitis C, tuberculosis, AIDS and other infectious diseases, organic disease such as diabetes, hypertension, coronary heart disease):

①Yes; ②No; ③Unclear

4.Detailed description (Followed the Question 3):

（1）What is the relationship with you ;

（2）Name of the disease ;

（3）With treatment followed: ①Yes; ②No

（4）Current situation__________________________________:

①Obviously improved; ②No change or slight change; ③Continuous deterioration;

5.For family members (mainly brothers and sisters), do they abusetobacco, alcohol or other addictive drugs ?

①Yes; ②No; ③Unclear;

6.Detailed description (Followed the Question 5):

（1）What is the relationship with you____________________?

（2）What do they abuse___________?
