# Supplementary material for: Association studies of dopamine synthesis and metabolism genes with multiple phenotypes of heroin dependence
Source: BMC Med Genet. 2020 Jul 31;21:157. doi: 10.1186/s12881-020-01092-0 (PMC7393710; doi:10.1186/s12881-020-01092-0)
Supplement: Supplementary file 2 — Additional file 2: Supplementary file 2: Table S1 The functional effects of all selected SNPs. Table S2 Results of association analysis between other SNPs and phenotype of memory. Table S3 Results of association analysis between other SNPs and age of onset for heroin use. Table S4 Results of association analysis between other SNPs and phenotype of euphoria. Table S5 Results of association analysis between SNPs and phenotype of methadone dosage. Table S6 Results of association analysis between SNPs and phenotype of heroin dosage. Table S7 Results of association analysis between SNPs and phenotype of DTFUD. Table S8 Transcription factor binding sites of rs1611114. Table S9 Transcription factor binding sites of rs10770140. Table S10 Transcription factor binding sites of rs10770141. [file 12881_2020_1092_MOESM2_ESM.docx]

**Table S1** The functional effects of all selected SNPs.

| **Genetic variation** | **Location** | **Prediction result** | **Possible functional effects** |
| --- | --- | --- | --- |
| **rs10064525** | 3’near | -- | -- |
| **rs27072** | 3’UTR | miRNA binding site | Transcriptional regulation |
| **rs1042098** | 3’UTR | miRNA binding site  ESE or ESS | Transcriptional regulation |
| **rs6347** | Exon | ESE or ESS | Transcriptional regulation |
| **rs10770140** | 5’near | TFBS | Transcriptional regulation |
| **rs10770141** | 5’near | TFBS | Transcriptional regulation |
| **rs3842727** | 3’near | TFBS | Transcriptional regulation |
| **rs6356** | Exon | nsSNP | missense |
| **rs11575553** | 3’UTR | miRNA binding site | Transcriptional regulation |
| **rs12666409** | 5’near | TFBS | Transcriptional regulation |
| **rs129882** | 3’UTR | miRNA binding site  TFBS | Transcriptional regulation |
| **rs129915** | 3’near | TFBS | Transcriptional regulation |
| **rs1611114** | 5’near | TFBS | Transcriptional regulation |
| **rs5320** | Exon | nsSNP | missense |

TFBS: transcription factor binding site.

ESE: Exonic Splicing Enhancer

ESS: Exonic Splicing Silencer

**Table S2** Results of association analysis between other SNPs and phenotype of memory

| Gene | Variable/ID | No change (n=215) | |  | Reduced (n=586) | | *P*^a^ | OR, 95% CI | *P*^b^ | Exp(B), 95% CI |
| --- | --- | --- | --- | --- | --- | --- | --- | --- | --- | --- |
|  |  | Number | Percent |  | Number | Percent |  |  |  |  |
| *SLC6A3* | rs10064525 |  |  |  |  |  | 0.617 |  | 0.746 |  |
|  | TT | 193 | 89.8 |  | 522 | 89.1 | 0.780 | 0.930, 0.557-1.551 | 0.703 | 0.903, 0.535-1.524 |
|  | TG | 21 | 9.8 |  | 63 | 10.8 | 0.687 | 1.113, 0.661-1.873 | 0.625 | 1.142, 0.671-1.944 |
|  | GG | 1 | 0.5 |  | 1 | 0.2 | 0.465 | 0.366, 0.023-5.874 | 0.549 | 0.418, 0.024-7.226 |
|  | T | 407 | 94.7 |  | 1107 | 94.5 | 0.878 | 1.039, 0.637-1.694 |  |  |
|  | G | 23 | 5.3 |  | 65 | 5.5 |  |  |  |  |
|  | rs27072 |  |  |  |  |  | 0.368 |  | 0.521 |  |
|  | CC | 131 | 60.9 |  | 325 | 55.5 | 0.166 | 0.798, 0.581-1.098 | 0.261 | 0.830, 0.599-1.149 |
|  | TC | 71 | 33.0 |  | 224 | 38.2 | 0.176 | 1.255, 0.903-1.745 | 0.281 | 1.204, 0.859-1.688 |
|  | TT | 13 | 6.0 |  | 37 | 6.3 | 0.890 | 1.047, 0.546-2.010 | 0.869 | 1.058, 0.544-2.057 |
|  | C | 333 | 77.4 |  | 874 | 74.6 | 0.238 | 1.171, 0.901-1.520 |  |  |
|  | T | 97 | 22.6 |  | 298 | 25.4 |  |  |  |  |
|  | rs1042098 |  |  |  |  |  | 0.413 |  | 0.382 |  |
|  | AA | 181 | 84.6 |  | 481 | 82.6 | 0.518 | 0.868, 0.566-1.333 | 0.598 | 0.889, 0.575-1.376 |
|  | GA | 30 | 14.0 |  | 97 | 16.7 | 0.366 | 1.227, 0.787-1.911 | 0.407 | 1.210, 0.771-1.900 |
|  | GG | 3 | 1.4 |  | 4 | 0.7 | 0.394 | 0.487, 0.108-2.193 | 0.252 | 0.407, 0.087-1.898 |
|  | A | 392 | 91.6 |  | 1059 | 91.0 | 0.115 | 0.720, 0.485-1.069 |  |  |
|  | G | 36 | 8.4 |  | 105 | 9.0 |  |  |  |  |
|  | rs6347 |  |  |  |  |  | 0.547 |  | 0.621 |  |
|  | TT | 161 | 74.9 |  | 454 | 77.5 | 0.442 | 1.154, 0.802-1.660 | 0.506 | 1.135, 0.782-1.646 |
|  | TC | 51 | 23.7 |  | 127 | 21.7 | 0.537 | 0.890, 0.614-1.289 | 0.631 | 0.911, 0.624-1.330 |
|  | CC | 3 | 1.4 |  | 5 | 0.9 | 0.449 | 0.608, 0.144-2.567 | 0.412 | 0.539, 0.123-2.362 |
|  | T | 373 | 86.7 |  | 1035 | 88.3 | 0.394 | 0.866, 0.622-1.206 |  |  |
|  | C | 57 | 13.3 |  | 137 | 11.7 |  |  |  |  |
| *TH* | rs10770140 |  |  |  |  |  | 0.951 |  | 0.964 |  |
|  | TT | 186 | 86.5 |  | 502 | 85.7 | 0.760 | 0.932, 0.592-1.468 | 0.703 | 0.914, 0.575-1.452 |
|  | TC | 29 | 13.5 |  | 82 | 14.0 | 0.855 | 1.044, 0.662-1.646 | 0.800 | 1.062, 0.667-1.690 |
|  | CC | 0 | 0.0 |  | 2 | 0.3 | 1.000 | 0.997, 0.992-1.001 | 0.999 | 633780115.4, 0.000- |
|  | T | 401 | 93.3 |  | 1086 | 92.7 | 0.683 | 1.095, 0.708-1.694 |  |  |
|  | C | 29 | 6.7 |  | 86 | 7.3 |  |  |  |  |
|  | rs10770141 |  |  |  |  |  | 0.899 |  | 0.915 |  |
|  | GG | 189 | 87.9 |  | 508 | 86.7 | 0.650 | 0.896, 0.558-1.440 | 0.592 | 0.876, 0.540-1.421 |
|  | GA | 26 | 12.1 |  | 76 | 13.0 | 0.742 | 1.083, 0.673-1.743 | 0.686 | 1.105, 0.681-1.795 |
|  | AA | 0 | 0.0 |  | 2 | 0.3 | 1.000 | 0.997, 0.992-1.001 | 0.999 | 633780115.4, 0.000- |
|  | G | 404 | 94.0 |  | 1092 | 93.2 | 0.578 | 1.138, 0.721-1.798 |  |  |
|  | A | 26 | 6.0 |  | 80 | 6.8 |  |  |  |  |
|  | rs3842727 |  |  |  |  |  | 0.596 |  | 0.807 |  |
|  | TT | 202 | 94.0 |  | 539 | 92.0 | 0.347 | 0.738, 0.391-1.393 | 0.462 | 0.784, 0.410-1.500 |
|  | TG | 13 | 6.0 |  | 46 | 7.8 | 0.387 | 1.324, 0.700-2.502 | 0.518 | 1.239, 0.647-2.375 |
|  | GG | 0 | 0.0 |  | 1 | 0.2 | 1.000 | 0.998, 0.995-1.002 | 1.000 | 703591981.7, 0.000- |
|  | T | 417 | 97.0 |  | 1124 | 95.9 | 0.320 | 1.370, 0.735-2.554 |  |  |
|  | G | 13 | 3.0 |  | 48 | 4.1 |  |  |  |  |
|  | rs6356 |  |  |  |  |  | 0.697 |  | 0.785 |  |
|  | TT | 160 | 74.4 |  | 426 | 72.7 | 0.626 | 0.915, 0.641-1.307 | 0.567 | 0.899, 0.625-1.293 |
|  | TC | 48 | 22.3 |  | 145 | 24.7 | 0.478 | 1.144, 0.789-1.659 | 0.497 | 1.141, 0.781-1.667 |
|  | CC | 7 | 3.3 |  | 15 | 2.6 | 0.593 | 0.781, 0.314-1.941 | 0.829 | 0.902, 0.355-2.295 |
|  | T | 368 | 85.6 |  | 997 | 85.1 | 0.798 | 1.024, 0.762-1.425 |  |  |
|  | C | 62 | 14.4 |  | 175 | 14.9 |  |  |  |  |
| *DDC* | rs11575553 |  |  |  |  |  | 0.790 |  | 0.647 |  |
|  | GG | 167 | 77.7 |  | 462 | 78.8 | 0.722 | 1.071, 0.734-1.562 | 0.662 | 1.090, 0.742-1.600 |
|  | GA | 43 | 20.0 |  | 107 | 18.3 | 0.576 | 0.894, 0.602-1.326 | 0.463 | 0.860, 0.574-1.287 |
|  | AA | 5 | 2.3 |  | 17 | 2.9 | 0.659 | 1.255, 0.457-3.444 | 0.530 | 1.390, 0.498-3.883 |
|  | G | 377 | 87.7 |  | 1031 | 88.0 | 0.873 | 0.973, 0.694-1.363 |  |  |
|  | A | 53 | 12.3 |  | 141 | 12.0 |  |  |  |  |
|  | rs12666409 |  |  |  |  |  | 0.589 |  | 0.589 |  |
|  | TT | 62 | 28.8 |  | 164 | 28.0 | 0.813 | 0.959, 0.679-1.355 | 0.938 | 0.986, 0.693-1.404 |
|  | TA | 109 | 50.7 |  | 282 | 48.1 | 0.518 | 0.902, 0.660-1.233 | 0.446 | 0.883, 0.641-1.216 |
|  | AA | 44 | 20.5 |  | 140 | 23.9 | 0.307 | 1.220, 0.833-1.787 | 0.322 | 1.218, 0.824-1.800 |
|  | T | 233 | 54.2 |  | 610 | 52.0 | 0.448 | 0.918, 0.735-1.145 |  |  |
|  | A | 197 | 45.8 |  | 562 | 48.0 |  |  |  |  |
| *DBH* | rs129882 |  |  |  |  |  | 0.853 |  | 0.649 |  |
|  | CC | 87 | 40.5 |  | 250 | 42.7 | 0.577 | 1.095, 0.797-1.504 | 0.355 | 1.166, 0.842-1.616 |
|  | TC | 98 | 45.6 |  | 256 | 43.7 | 0.632 | 0.926, 0.676-1.268 | 0.521 | 0.900, 0.653-1.241 |
|  | TT | 30 | 14.0 |  | 80 | 13.7 | 0.912 | 0.975, 0.620-1.532 | 0.687 | 0.909, 0.573-1.444 |
|  | C | 272 | 63.3 |  | 756 | 64.5 | 0.644 | 0.947, 0.753-1.192 |  |  |
|  | T | 158 | 36.7 |  | 416 | 35.5 |  |  |  |  |
|  | rs129915 |  |  |  |  |  | 0.234 |  | 0.157 |  |
|  | AA | 74 | 34.4 |  | 167 | 28.5 | 0.105 | 0.759, 0.544-1.060 | 0.061 | 0.722, 0.513-1.016 |
|  | GA | 100 | 46.5 |  | 307 | 52.4 | 0.043 | 1.375, 1.010-1.873 | 0.113 | 1.296, 0.941-1.784 |
|  | GG | 41 | 19.1 |  | 112 | 19.1 | 0.989 | 1.003, 0.674-1.493 | 0.875 | 1.033, 0.687-1.553 |
|  | A | 248 | 57.7 |  | 641 | 54.7 | 0.287 | 1.129, 0.903-1.411 |  |  |
|  | G | 182 | 42.3 |  | 531 | 45.3 |  |  |  |  |
|  | rs5320 |  |  |  |  |  | 0.478 |  | 0.370 |  |
|  | GG | 166 | 77.2 |  | 427 | 72.9 | 0.214 | 0.793, 0.549-1.144 | 0.168 | 0.768, 0.528-1.118 |
|  | GA | 45 | 20.9 |  | 147 | 25.1 | 0.222 | 1.265, 0.867-1.846 | 0.161 | 1.318, 0.896-1.940 |
|  | AA | 4 | 1.9 |  | 12 | 2.0 | 1.000 | 1.103, 0.352-3.457 | 0.971 | 1.022, 0.319-3.280 |
|  | G | 377 | 87.7 |  | 1001 | 85.4 | 0.247 | 1.215, 0.873-1.690 |  |  |
|  | A | 53 | 12.3 |  | 171 | 14.6 |  |  |  |  |

*P*^a^: Genotypic and allele frequency difference assessment by chi-square test.

*P*^b^: Genotypic and allele frequency difference assessment by binary logistic regression.

**Table S3** Results of association analysis between other SNPs and age of onset for heroin use

| Gene | Variable/ID | Age≤28(n=440) | |  | Age>28(n=361) | | *P*^a^ | OR, 95% CI | *P*^b^ | Exp(B), 95% CI |
| --- | --- | --- | --- | --- | --- | --- | --- | --- | --- | --- |
|  |  | Number | Percent |  | Number | Percent |  |  |  |  |
| *SLC6A3* | rs10064525 |  |  |  |  |  | 0.597 |  | 0.342 |  |
|  | TT | 393 | 89.3 |  | 322 | 89.2 | 0.956 | 1.013, 0.646-1.587 | 0.200 | 1.412, 0.833-2.392 |
|  | TG | 45 | 10.2 |  | 39 | 10.8 | 0.791 | 0.941, 0.598-1.480 | 0.138 | 0.668, 0.392-1.139 |
|  | GG | 2 | 0.5 |  | 0 | 0.0 | 0.504 | 0.995, 0.989-1.002 | 0.999 | 0.000, 0.000- |
|  | T | 831 | 94.4 |  | 683 | 94.6 | 0.884 | 0.968, 0.628-1.493 |  |  |
|  | G | 49 | 5.6 |  | 39 | 5.4 |  |  |  |  |
|  | rs27072 |  |  |  |  |  | 0.375 |  | 0.691 |  |
|  | CC | 244 | 55.5 |  | 212 | 58.7 | 0.352 | 0.875, 0.660-1.159 | 0.526 | 0.898, 0.646-1.250 |
|  | TC | 171 | 38.9 |  | 124 | 34.3 | 0.187 | 1.215, 0.909- 1.623 | 0.403 | 1.156, 0.822-1.626 |
|  | TT | 25 | 5.7 |  | 25 | 6.9 | 0.469 | 0.810, 0.457-1.436 | 0.724 | 0.887, 0.455-1.730 |
|  | C | 659 | 74.9 |  | 548 | 75.9 | 0.639 | 0.947, 0.753-1.190 |  |  |
|  | T | 221 | 25.1 |  | 174 | 24.1 |  |  |  |  |
|  | rs1042098 |  |  |  |  |  | 0.797 |  | 0.721 |  |
|  | AA | 363 | 82.9 |  | 299 | 83.5 | 0.809 | 0.955, 0.657-1.388 | 0.436 | 0.840, 0.543-1.300 |
|  | GA | 72 | 16.4 |  | 55 | 15.4 | 0.680 | 1.084, 0.739-1.589 | 0.419 | 1.202, 0.769-1.880 |
|  | GG | 3 | 0.7 |  | 4 | 1.1 | 0.707 | 0.610, 0.136-2.745 | 0.961 | 0.958, 0.169-5.435 |
|  | A | 798 | 91.1 |  | 653 | 91.2 | 1.000 | 0.987, 0.697-1.398 |  |  |
|  | G | 78 | 8.9 |  | 63 | 8.8 |  |  |  |  |
|  | rs6347 |  |  |  |  |  | 0.559 |  | 0.819 |  |
|  | TT | 332 | 75.5 |  | 283 | 78.4 | 0.327 | 0.847, 0.608-1.180 | 0.546 | 0.889, 0.604-1.306 |
|  | TC | 104 | 23.6 |  | 74 | 20.5 | 0.288 | 1.200, 0.857-1.682 | 0.527 | 1.136, 0.767-1.681 |
|  | CC | 4 | 0.9 |  | 4 | 1.1 | 1.000 | 0.819, 0.203-3.297 | 0.943 | 0.946, 0.203-4.425 |
|  | T | 768 | 87.3 |  | 640 | 88.6 | 0.403 | 0.879, 0.649-1.190 |  |  |
|  | C | 112 | 12.7 |  | 82 | 11.4 |  |  |  |  |
| *TH* | rs3842727 |  |  |  |  |  | 0.629 |  | 0.811 |  |
|  | TT | 404 | 91.8 |  | 337 | 93.4 | 0.412 | 0.799, 0.467-1.366 | 0.495 | 0.808, 0.438-1.491 |
|  | TG | 35 | 8.0 |  | 24 | 6.6 | 0.481 | 1.213, 0.708-2.081 | 0.520 | 1.224, 0.662-2.263 |
|  | GG | 1 | 0.2 |  | 0 | 0.0 | 1.000 | 1.002, 0.998-1.007 | 1.000 | 0.000, 0.000- |
|  | T | 843 | 95.8 |  | 698 | 96.7 | 0.360 | 0.783, 0.464-1.322 |  |  |
|  | G | 37 | 4.2 |  | 24 | 3.3 |  |  |  |  |
|  | rs6356 |  |  |  |  |  | 0.417 |  | 0.215 |  |
|  | TT | 322 | 73.2 |  | 264 | 73.2 | 0.987 | 1.003, 0.732-1.373 | 0.247 | 1.241, 0.861-1.787 |
|  | TC | 103 | 23.4 |  | 90 | 24.1 | 0.616 | 0.920, 0.665-1.274 | 0.552 | 0.892, 0.613-1.299 |
|  | CC | 15 | 3.4 |  | 7 | 2.7 | 0.061 | 2.334, 0.940-5.797 | 0.113 | 2.387, 0.814-6.994 |
|  | T | 747 | 84.9 |  | 618 | 85.6 | 0.691 | 0.945, 0.716-1.248 |  |  |
|  | C | 133 | 15.1 |  | 104 | 14.4 |  |  |  |  |
| *DDC* | rs11575553 |  |  |  |  |  | 0.438 |  | 0.332 |  |
|  | GG | 342 | 77.7 |  | 287 | 79.5 | 0.543 | 0.900, 0.640-1.264 | 0.485 | 0.869, 0.584-1.292 |
|  | GA | 83 | 18.9 |  | 67 | 18.6 | 0.913 | 1.020, 0.714-1.458 | 0.921 | 1.022, 0.673-1.553 |
|  | AA | 15 | 3.4 |  | 7 | 1.9 | 0.205 | 1.785, 0.720-4.426 | 0.141 | 2.198, 0.769-6.289 |
|  | G | 767 | 87.2 |  | 641 | 88.8 | 0.322 | 0.858, 0.633-1.162 |  |  |
|  | A | 113 | 12.8 |  | 81 | 11.2 |  |  |  |  |
|  | rs12666409 |  |  |  |  |  | 0.805 |  | 0.723 |  |
|  | TT | 120 | 27.3 |  | 106 | 29.4 | 0.513 | 0.902, 0.662-1.228 | 0.845 | 0.966,0.676-1.378 |
|  | TA | 218 | 49.5 |  | 173 | 47.9 | 0.648 | 1.067, 0.808-1.410 | 0.463 | 1.13, 0.815-1.568 |
|  | AA | 102 | 23.2 |  | 82 | 22.7 | 0.876 | 1.027, 0.737-1.430 | 0.507 | 1.141, 0.773-1.686 |
|  | T | 458 | 52.0 |  | 385 | 53.3 | 0.610 | 1.053, 0.864-1.282 |  |  |
|  | A | 422 | 48.0 |  | 337 | 46.7 |  |  |  |  |
| *DBH* | rs129882 |  |  |  |  |  | 0.157 |  | 0.275 |  |
|  | CC | 198 | 45.0 |  | 139 | 38.5 | 0.064 | 1.307, 0.984-1.735 | 0.131 | 1.292, 0.927-1.806 |
|  | TC | 187 | 42.5 |  | 167 | 46.3 | 0.286 | 0.859, 0.649-1.136 | 0.421 | 0.875, 0.630-1.214 |
|  | TT | 55 | 12.5 |  | 55 | 15.2 | 0.263 | 0.795, 0.531-1.189 | 0.312 | 0.781, 0.483-1.263 |
|  | C | 583 | 66.3 |  | 445 | 61.6 | 0.055 | 1.222, 0.995-1.500 |  |  |
|  | T | 297 | 33.8 |  | 277 | 38.4 |  |  |  |  |
|  | rs129915 |  |  |  |  |  | 0.233 |  | 0.067 |  |
|  | AA | 127 | 28.9 |  | 241 | 30.1 | 0.652 | 0.943, 0.730-1.217 | 0.296 | 0.827, 0.579-1.181 |
|  | GA | 211 | 48.0 |  | 407 | 50.8 | 0.336 | 0.892, 0.707-1.126 | 0.389 | 0.867, 0.627-1.201 |
|  | GG | 102 | 23.2 |  | 153 | 19.1 | 0.089 | 1.278, 0.963-1.696 | 0.021 | 1.648, 1.078-2.519 |
|  | A | 465 | 52.8 |  | 889 | 55.5 | 0.204 | 0.899, 0.762-1.060 |  |  |
|  | G | 415 | 47.2 |  | 713 | 44.5 |  |  |  |  |
|  | rs1611114 |  |  |  |  |  | 0.984 |  | 0.834 |  |
|  | TT | 187 | 42.5 |  | 155 | 42.9 | 0.901 | 0.982, 0.741-1.302 | 0.857 | 0.970, 0.697-1.351 |
|  | TC | 199 | 45.2 |  | 161 | 44.6 | 0.859 | 1.026, 0.775-1.357 | 0.832 | 1.037, 0.748-1.435 |
|  | CC | 54 | 12.3 |  | 45 | 12.5 | 0.934 | 0.982, 0.644-1.499 | 0.547 | 0.857, 0.519-1.417 |
|  | T | 573 | 65.1 |  | 471 | 65.2 | 0.959 | 0.995, 0.809-1.223 |  |  |
|  | C | 307 | 34.9 |  | 251 | 34.8 |  |  |  |  |
|  | rs5320 |  |  |  |  |  | 0.831 |  | 0.668 |  |
|  | GG | 322 | 73.2 |  | 271 | 75.1 | 0.544 | 0.906, 0.659-1.246 | 0.369 | 0.845, 0.584-1.222 |
|  | GA | 109 | 24.8 |  | 83 | 23.0 | 0.557 | 1.103, 0.795-1.530 | 0.387 | 1.183, 0.809-1.728 |
|  | AA | 9 | 2.0 |  | 7 | 1.9 | 0.915 | 1.056, 0.389-2.864 | 0.867 | 1.103, 0.350-3.485 |
|  | G | 753 | 85.6 |  | 625 | 86.6 | 0.567 | 0.920, 0.692-1.223 |  |  |
|  | A | 127 | 14.4 |  | 97 | 13.4 |  |  |  |  |

*P*^a^: Genotypic and allele frequency difference assessment by chi-square test.

*P*^b^: Genotypic and allele frequency difference assessment by binary logistic regression.

**Table S4** Results of association analysis between other SNPs and phenotype of euphoria

| Gene | Variable/ID | Weak（n=628） | |  | Strong (n=173) | | *P*^b^ | OR, 95% CI | *P*^c^ | Exp(B), 95% CI |
| --- | --- | --- | --- | --- | --- | --- | --- | --- | --- | --- |
|  |  | Number | Percent |  | Number | Percent |  |  |  |  |
| *SLC6A3* | rs10064525 |  |  |  |  |  | 0.634 |  | 0.670 |  |
|  | TT | 557 | 88.7 |  | 158 | 91.3 | 0.322 | 1.343, 0.749-2.408 | 0.318 | 1.356, 0.746-2.462 |
|  | TG | 69 | 5.5 |  | 15 | 8.7 | 0.379 | 1.300, 0.724-2.335 | 0.378 | 1.309,0.719-2.381 |
|  | GG | 2 | 0.2 |  | 0 | 0.0 | 1.000 | 1.003, 0.999-1.008 | 0.999 | 0.000, 0.000- |
|  | T | 1183 | 94.2 |  | 331 | 95.7 | 0.286 | 0.734, 0.416-1.297 |  |  |
|  | G | 73 | 5.8 |  | 15 | 4.3 |  |  |  |  |
|  | rs27072 |  |  |  |  |  | 0.132 |  | 0.227 |  |
|  | CC | 368 | 58.6 |  | 88 | 50.9 | 0.069 | 0.731, 0.522-1.025 | 0.105 | 0.751, 0.532-1.061 |
|  | TC | 220 | 18.1 |  | 75 | 43.4 | 0.045 | 1.419, 1.008-1.999 | 0.086 | 1.357, 0.955-1.928 |
|  | TT | 40 | 3.1 |  | 10 | 5.8 | 0.777 | 1.109,0.543-2.268 | 0.941 | 1.028,0.496-2.132 |
|  | C | 956 | 76.1 |  | 251 | 72.5 | 0.172 | 1.206, 0.921-1.579 |  |  |
|  | T | 300 | 23.9 |  | 95 | 27.5 |  |  |  |  |
|  | rs1042098 |  |  |  |  |  | 0.664 |  | 0.568 |  |
|  | AA | 522 | 83.7 |  | 140 | 81.4 | 0.483 | 0.855, 0.551-1.326 | 0.541 | 0.869, 0.554-1.363 |
|  | GA | 96 | 7.7 |  | 31 | 18.0 | 0.403 | 1.209, 0.775-1.888 | 0.411 | 1.211, 0.767-1.912 |
|  | GG | 6 | 0.5 |  | 1 | 0.6 | 1.000 | 1.661, 0.199-13.889 | 0.476 | 2.198,0.252-19.231 |
|  | A | 1140 | 91.3 |  | 311 | 90.4 | 0.587 | 1.120, 0.744-1.687 |  |  |
|  | G | 108 | 8.7 |  | 33 | 9.6 |  |  |  |  |
|  | rs6347 |  |  |  |  |  | 0.825 |  | 0.858 |  |
|  | TT | 484 | 77.1 |  | 131 | 75.7 | 0.710 | 0.928, 0.626-1.376 | 0.580 | 0.892, 0.596-1.337 |
|  | TC | 138 | 11.0 |  | 40 | 23.1 | 0.748 | 1.068, 0.715-1.594 | 0.597 | 1.117, 0.741-1.684 |
|  | CC | 6 | 0.5 |  | 2 | 1.2 | 0.685 | 1.212, 0.243-6.061 | 0.890 | 1.127, 0.206-6.153 |
|  | T | 1106 | 88.1 |  | 302 | 87.3 | 0.696 | 1.074, 0.750-1.539 |  |  |
|  | C | 150 | 11.9 |  | 44 | 12.7 |  |  |  |  |
| *TH* | rs3842727 |  |  |  |  |  | 0.238 |  | 0.647 |  |
|  | TT | 580 | 92.4 |  | 161 | 93.1 | 0.754 | 1.110, 0.576-2.140 | 0.498 | 1.261, 0.645-2.465 |
|  | TG | 48 | 3.8 |  | 11 | 6.4 | 0.567 | 1.219,0.619-2.398 | 0.339 | 1.401, 0.702-2.801 |
|  | GG | 0 | 0.0 |  | 1 | 0.6 | 0.216 | 1.006, 0.994-1.017 | 1.000 | 9666057428, 0.000- |
|  | T | 1208 | 96.2 |  | 333 | 96.2 | 0.956 | 0.982, 0.526-1.835 |  |  |
|  | G | 48 | 3.8 |  | 13 | 3.8 |  |  |  |  |
|  | rs6356 |  |  |  |  |  | 0.682 |  | 0.590 |  |
|  | TT | 458 | 72.9 |  | 128 | 74.0 | 0.781 | 1.056, 0.720-1.548 | 0.901 | 1.025, 0.693-1.516 |
|  | TC | 154 | 12.4 |  | 39 | 22.5 | 0.590 | 1.116, 0.748-1.667 | 0.632 | 1.105, 0.734-1.664 |
|  | CC | 16 | 1.3 |  | 6 | 3.5 | 0.598 | 1.374, 0.529-3.567 | 0.343 | 1.613, 0.601-4.329 |
|  | T | 1070 | 85.2 |  | 295 | 85.3 | 0.974 | 0.995, 0.711-1.391 |  |  |
|  | C | 186 | 14.8 |  | 51 | 14.7 |  |  |  |  |
| *DDC* | rs11575553 |  |  |  |  |  | 0.695 |  | 0.790 |  |
|  | GG | 493 | 78.5 |  | 136 | 78.6 | 0.975 | 1.007, 0.668-1.517 | 0.833 | 1.046, 0.688-1.591 |
|  | GA | 116 | 9.4 |  | 34 | 19.7 | 0.724 | 1.080, 0.705-1.653 | 0.956 | 1.012, 0.654-1.567 |
|  | AA | 19 | 1.5 |  | 3 | 1.7 | 0.442 | 1.767,0.517-6.061 | 0.492 | 1.550,0.444-5.405 |
|  | G | 1102 | 87.7 |  | 306 | 88.4 | 0.724 | 0.935, 0.646-1.354 |  |  |
|  | A | 154 | 12.3 |  | 40 | 11.6 |  |  |  |  |
|  | rs12666409 |  |  |  |  |  | 0.713 |  | 0.651 |  |
|  | TT | 176 | 12.3 |  | 50 | 28.9 | 0.821 | 1.044, 0.720-1.514 | 0.747 | 1.065, 0.728-1.558 |
|  | TA | 311 | 28.8 |  | 80 | 46.2 | 0.445 | 1.140,0.814-1.597 | 0.377 | 1.168,0.827-1.650 |
|  | AA | 141 | 22.5 |  | 43 | 24.9 | 0.506 | 1.142, 0.772-1.692 | 0.479 | 1.157, 0.773-1.731 |
|  | T | 663 | 52.8 |  | 180 | 52.0 | 0.801 | 0.970, 0.764-1.231 |  |  |
|  | A | 593 | 47.2 |  | 166 | 48.0 |  |  |  |  |
| *DBH* | rs129882 |  |  |  |  |  | 0.628 |  | 0.600 |  |
|  | CC | 268 | 42.7 |  | 69 | 39.9 | 0.510 | 0.891, 0.632-1.256 | 0.697 | 0.933, 0.656-1.326 |
|  | TC | 272 | 23.3 |  | 82 | 47.4 | 0.338 | 1.179, 0.841-1.653 | 0.363 | 1.174, 0.831-1.658 |
|  | TT | 88 | 6.5 |  | 22 | 12.7 | 0.661 | 1.119,0.678-1.845 | 0.455 | 1.215, 0.728-2.028 |
|  | C | 808 | 64.3 |  | 220 | 63.6 | 0.797 | 1.033, 0.806-1.323 |  |  |
|  | T | 448 | 35.7 |  | 126 | 36.4 |  |  |  |  |
|  | rs129915 |  |  |  |  |  | 0.143 |  | 0.105 |  |
|  | AA | 191 | 30.4 |  | 50 | 28.9 | 0.701 | 0.930, 0.642-1.347 | 0.613 | 0.907, 0.622-1.323 |
|  | GA | 326 | 28.5 |  | 81 | 46.8 | 0.236 | 1.225, 0.875-1.718 | 0.234 | 1.233, 0.873-1.739 |
|  | GG | 111 | 8.1 |  | 42 | 24.3 | 0.050 | 1.493, 0.997-2.236 | 0.034 | 1.569, 1.034-2.379 |
|  | A | 708 | 56.4 |  | 181 | 52.3 | 0.179 | 1.178, 0.928-1.795 |  |  |
|  | G | 548 | 43.6 |  | 165 | 47.7 |  |  |  |  |
|  | rs1611114 |  |  |  |  |  | 0.537 |  | 0.523 |  |
|  | TT | 274 | 43.6 |  | 68 | 39.3 | 0.309 | 0.837, 0.593-1.180 | 0.333 | 0.841, 0.592-1.194 |
|  | TC | 276 | 23.4 |  | 84 | 48.6 | 0.281 | 1.204, 0.859-1.686 | 0.258 | 1.209, 0.864-1.720 |
|  | CC | 78 | 5.8 |  | 21 | 12.1 | 0.921 | 1.027,0.614-1.715 | 0.797 | 1.072,0.630-1.825 |
|  | T | 824 | 65.6 |  | 220 | 63.6 | 0.485 | 1.092, 0.852-1.400 |  |  |
|  | C | 432 | 34.4 |  | 126 | 36.4 |  |  |  |  |
|  | rs5320 |  |  |  |  |  | 0.243 |  | 0.266 |  |
|  | GG | 469 | 74.7 |  | 124 | 71.7 | 0.425 | 0.858, 0.589-1.250 | 0.303 | 0.816, 0.555-1.201 |
|  | GA | 149 | 12.0 |  | 43 | 24.9 | 0.758 | 1.063, 0.719-1.572 | 0.578 | 1.120, 0.750-1.673 |
|  | AA | 10 | 0.8 |  | 6 | 3.5 | 0.128 | 2.220, 0.795-6.197 | 0.139 | 2.227, 0.771-6.443 |
|  | G | 1087 | 86.5 |  | 291 | 84.1 | 0.246 | 1.216, 0.873-1.692 |  |  |
|  | A | 169 | 13.5 |  | 55 | 15.9 |  |  |  |  |

*P*^a^: Genotypic and allele frequency difference assessment by chi-square test.

*P*^b^: Genotypic and allele frequency difference assessment by binary logistic regression.

**Table S5** Results of association analysis between SNPs and phenotype of methadone dosage

| Gene | Variable/ID | Methadone dose  ≤ 45mg (n=350) | |  | Methadone dose  >45mg (n=285) | | *P*^a^ | OR, 95% CI | *P*^b^ | Exp(B), 95% CI |
| --- | --- | --- | --- | --- | --- | --- | --- | --- | --- | --- |
|  |  | Number | Percent |  | Number | Percent |  |  |  |  |
| *SLC6A3* | rs10064525 |  |  |  |  |  | 0.449 |  | 0.682 |  |
|  | TT | 318 | 90.9 |  | 250 | 87.7 | 0.200 | 0.719, 0.433-1.194 | 0.385 | 0.794, 0.472-1.336 |
|  | TG | 31 | 8.9 |  | 34 | 11.9 | 0.204 | 1.394, 0.834-2.331 | 0.402 | 1.253, 0.740-2.122 |
|  | GG | 1 | 0.3 |  | 1 | 0.4 | 1.000 | 1.229, 0.077-19.734 | 0.816 | 1.400, 0.082-23.889 |
|  | T | 667 | 95.3 |  | 534 | 93.7 | 0.210 | 1.363, 0.838-2.215 |  |  |
|  | G | 33 | 4.7 |  | 36 | 6.3 |  |  |  |  |
|  | rs27072 |  |  |  |  |  | 0.539 |  | 0.693 |  |
|  | CC | 201 | 57.4 |  | 173 | 60.7 | 0.404 | 1.145, 0.833-1.574 | 0.648 | 1.080, 0.775-1.506 |
|  | TC | 124 | 35.4 |  | 98 | 34.4 | 0.784 | 0.955, 0.688-1.326 | 0.959 | 0.991, 0.702-1.399 |
|  | TT | 25 | 7.1 |  | 14 | 4.9 | 0.244 | 0.672, 0.342-1.317 | 0.405 | 0.744, 0.371-1.493 |
|  | C | 526 | 75.1 |  | 444 | 77.9 | 0.251 | 0.858, 0.660-1.115 |  |  |
|  | T | 174 | 24.9 |  | 126 | 22.1 |  |  |  |  |
|  | rs1042098 |  |  |  |  |  | 0.141 |  | 0.430 |  |
|  | AA | 281 | 80.3 |  | 242 | 85.8 | 0.067 | 1.486, 0.971-2.274 | 0.100 | 1.459, 0.930-2.289 |
|  | GA | 66 | 18.9 |  | 37 | 13.1 | 0.052 | 0.650, 0.420-1.006 | 0.099 | 0.678, 0.428-1.075 |
|  | GG | 3 | 0.9 |  | 3 | 1.1 | 1.000 | 1.244, 0.249-6.210 | 0.908 | 1.107, 0.163-4.329 |
|  | A | 628 | 89.7 |  | 521 | 92.4 | 0.102 | 0.720, 0.485-1.069 |  |  |
|  | G | 72 | 10.3 |  | 43 | 7.6 |  |  |  |  |
|  | rs6347 |  |  |  |  |  | 0.426 |  | 0.520 |  |
|  | TT | 270 | 77.1 |  | 218 | 76.5 | 0.846 | 0.964, 0.666-1.396 | 0.740 | 0.937, 0.637-1.378 |
|  | TC | 78 | 22.3 |  | 62 | 21.8 | 0.872 | 0.970, 0.665-1.414 | 0.967 | 0.992, 0.669-1.470 |
|  | CC | 2 | 0.6 |  | 5 | 1.8 | 0.252 | 3.107, 0.598-16.136 | 0.255 | 2.691, 0.490-14.779 |
|  | T | 618 | 88.3 |  | 498 | 87.4 | 0.618 | 1.090, 0.777-1.528 |  |  |
|  | C | 82 | 11.7 |  | 72 | 12.6 |  |  |  |  |
| *TH* | rs10770140 |  |  |  |  |  | 0.409 |  | 0.352 |  |
|  | TT | 301 | 86.0 |  | 234 | 82.1 | 0.180 | 0.747, 0.487-1.146 | 0.151 | 0.717, 0.456-1.129 |
|  | TC | 48 | 13.7 |  | 50 | 17.5 | 0.184 | 1.399, 0.870-2.060 | 0.164 | 1.384, 0.876-2.188 |
|  | CC | 1 | 0.3 |  | 1 | 0.4 | 1.000 | 1.229, 0.077-19.734 | 0.720 | 1.665, 0.103-26.946 |
|  | T | 650 | 92.9 |  | 518 | 90.9 | 0.213 | 1.305, 0.870-1.957 |  |  |
|  | C | 50 | 7.1 |  | 52 | 9.1 |  |  |  |  |
|  | rs10770141 |  |  |  |  |  | 0.244 |  | 0.243 |  |
|  | GG | 306 | 87.4 |  | 237 | 83.2 | 0.128 | 0.710, 0.456-1.105 | 0.094 | 0.668, 0.417-1.071 |
|  | GA | 43 | 12.3 |  | 47 | 16.5 | 0.131 | 1.410, 0.902-2.204 | 0.102 | 1.488, 0.924-2.396 |
|  | AA | 1 | 0.3 |  | 1 | 0.4 | 1.000 | 1.229, 0.077-19.734 | 0.720 | 1.665, 0.103-26.946 |
|  | G | 655 | 93.6 |  | 521 | 91.4 | 0.142 | 1.369, 0.899-2.085 |  |  |
|  | A | 45 | 6.4 |  | 49 | 8.6 |  |  |  |  |
|  | rs3842727 |  |  |  |  |  | 0.495 |  | 0.795 |  |
|  | TT | 325 | 92.9 |  | 261 | 91.6 | 0.548 | 0.837, 0.467-1.499 | 0.397 | 0.764, 0.411-1.423 |
|  | TG | 25 | 7.1 |  | 23 | 8.1 | 0.660 | 1.141, 0.633-2.057 | 0.512 | 1.234, 0.658-2.314 |
|  | GG | 0 | 0.0 |  | 1 | 0.4 | 0.457 | 0.997, 0.990-1.003 | 1.000 | 2745372121, 0.000- |
|  | T | 675 | 96.4 |  | 545 | 95.6 | 0.458 | 1.239, 0.703-2.181 |  |  |
|  | G | 25 | 3.6 |  | 25 | 4.4 |  |  |  |  |
|  | rs6356 |  |  |  |  |  | 0.518 |  | 0.498 |  |
|  | TT | 256 | 73.1 |  | 199 | 69.8 | 0.356 | 0.850, 0.601-1.201 | 0.663 | 0.922, 0.640-1.328 |
|  | TC | 84 | 24.0 |  | 74 | 26.0 | 0.569 | 1.111, 0.774-1.593 | 0.968 | 1.008, 0.689-1.474 |
|  | CC | 10 | 2.9 |  | 12 | 4.2 | 0.354 | 1.495, 0.636-3.511 | 0.239 | 1.716, 0.699-4。217 |
|  | T | 596 | 85.1 |  | 472 | 82.8 | 0.258 | 1.190, 0.880-1.608 |  |  |
|  | C | 104 | 14.9 |  | 98 | 17.2 |  |  |  |  |
| *DDC* | rs11575553 |  |  |  |  |  | 0.795 |  | 0.867 |  |
|  | GG | 281 | 80.3 |  | 227 | 79.6 | 0.842 | 0.961, 0.650-1.420 | 0.671 | 0.916, 0.610-1.375 |
|  | GA | 61 | 17.4 |  | 49 | 17.2 | 0.938 | 0.984, 0.651-1.487 | 0.800 | 0.946, 0.614-1.456 |
|  | AA | 8 | 2.3 |  | 9 | 3.2 | 0.498 | 1.394, 0.531-3.661 | 0.652 | 1.255, 0.468-3.368 |
|  | G | 623 | 89.0 |  | 503 | 88.2 | 0.673 | 1.078, 0.761-1.526 |  |  |
|  | A | 77 | 11.0 |  | 67 | 11.8 |  |  |  |  |
|  | rs12666409 |  |  |  |  |  | 0.243 |  | 0.214 |  |
|  | TT | 100 | 28.6 |  | 81 | 28.4 | 0.967 | 0.993, 0.702-1.404 | 0.851 | 0.966, 0.675-1.384 |
|  | TA | 179 | 51.1 |  | 131 | 46.0 | 0.194 | 0.813, 0.594-1.112 | 0.207 | 0.811, 0.585-1.123 |
|  | AA | 71 | 20.3 |  | 73 | 25.6 | 0.111 | 1.353, 0.932-1.963 | 0.088 | 1.398, 0.951-2.056 |
|  | T | 379 | 54.1 |  | 293 | 51.4 | 0.331 | 0.896, 0.718-1.118 |  |  |
|  | A | 321 | 45.9 |  | 277 | 48.6 |  |  |  |  |
| *DBH* | rs129882 |  |  |  |  |  | 0.842 |  | 0.897 |  |
|  | CC | 153 | 43.7 |  | 118 | 41.4 | 0.558 | 0.910, 0.663-1.249 | 0.644 | 0.925, 0.665-1.287 |
|  | TC | 153 | 43.7 |  | 130 | 45.6 | 0.632 | 1.080, 0.788-1.479 | 0.749 | 1.055, 0.759-1.466 |
|  | TT | 44 | 12.6 |  | 37 | 13.0 | 0.877 | 1.038, 0.650-1.657 | 0.830 | 1.056, 0.640-1.742 |
|  | C | 459 | 65.6 |  | 366 | 64.2 | 0.613 | 1.062, 0.842-1.338 |  |  |
|  | T | 241 | 34.4 |  | 204 | 35.8 |  |  |  |  |
|  | rs129915 |  |  |  |  |  | 0.136 |  | 0.167 |  |
|  | AA | 91 | 26.0 |  | 94 | 33.0 | 0.054 | 1.401, 0.993-1.975 | 0.074 | 1.386, 0.968-1.982 |
|  | GA | 185 | 52.9 |  | 132 | 46.3 | 0.101 | 0.769, 0.562-1.053 | 0.103 | 0.762, 0.549-1.056 |
|  | GG | 74 | 21.1 |  | 59 | 20.7 | 0.964 | 0.991, 0.675-1.456 | 0.991 | 0.998, 0.666-1.495 |
|  | A | 367 | 52.4 |  | 320 | 56.1 | 0.187 | 0.861, 0.689-1.075 |  |  |
|  | G | 333 | 47.6 |  | 250 | 43.9 |  |  |  |  |
|  | rs1611114 |  |  |  |  |  | 0.963 |  | 0.891 |  |
|  | TT | 151 | 43.1 |  | 124 | 43.5 | 0.926 | 1.015, 0.740-1.392 | 0.631 | 1.084, 0.781-1.503 |
|  | TC | 156 | 44.6 |  | 128 | 44.9 | 0.932 | 1.014, 0.740-1.389 | 0.699 | 1.067, 0.768-1.483 |
|  | CC | 43 | 12.3 |  | 33 | 11.6 | 0.785 | 0.935, 0.577-1.516 | 0.883 | 0.962, 0.578-1.601 |
|  | T | 458 | 65.4 |  | 376 | 66.0 | 0.841 | 0.976, 0.774-1.233 |  |  |
|  | C | 242 | 34.6 |  | 194 | 34.0 |  |  |  |  |
|  | rs5320 |  |  |  |  |  | 0.626 |  | 0.494 |  |
|  | GG | 259 | 74.0 |  | 219 | 76.8 | 0.409 | 1.166, 0.810-1.678 | 0.235 | 1.262, 0.860-1.852 |
|  | GA | 86 | 24.6 |  | 61 | 21.4 | 0.347 | 0.836, 0.576-1.214 | 0.254 | 0.796, 0.537-1.179 |
|  | AA | 5 | 1.4 |  | 5 | 1.8 | 0.759 | 1.232, 0.353-4.299 | 0.808 | 1.167, 0.336-4.055 |
|  | G | 604 | 86.3 |  | 499 | 87.5 | 0.509 | 0.895, 0.644-1.244 |  |  |
|  | A | 96 | 13.7 |  | 71 | 12.5 |  |  |  |  |

*P*^a^: Genotypic and allele frequency difference assessment by chi-square test.

*P*^b^: Genotypic and allele frequency difference assessment by binary logistic regression.

**Table S6** Results of association analysis between SNPs and phenotype of heroin dosage

| Gene | Variable/ID | Heroin dosage  ≤0.5g (n=426) | |  | Heroin dosage  >0.5g (n=375) | | *P*^a^ | OR, 95% CI | *P*^b^ | OR, 95% CI |
| --- | --- | --- | --- | --- | --- | --- | --- | --- | --- | --- |
|  |  | Number | Percent |  | Number | Percent |  |  |  |  |
| *SLC6A3* | rs10064525 |  |  |  |  |  | 0.745 |  | 0.626 |  |
|  | TT | 377 | 88.5 |  | 338 | 90.1 | 0.456 | 1.187, 0.756-1.865 | 0.346 | 1.253, 0.784-2.002 |
|  | TG | 48 | 11.3 |  | 36 | 9.6 | 0.442 | 0.836, 0.530-1.320 | 0.334 | 0.792, 0.493-1.272 |
|  | GG | 1 | 0.2 |  | 1 | 0.3 | 1.000 | 1.136, 0.071-18.231 | 0.934 | 1.127, 0.066-19.128 |
|  | T | 802 | 94.1 |  | 712 | 94.9 | 0.482 | 0.856, 0.555-1.321 |  |  |
|  | G | 50 | 5.9 |  | 38 | 5.1 |  |  |  |  |
|  | rs27072 |  |  |  |  |  | 0.317 |  | 0.206 |  |
|  | CC | 250 | 58.7 |  | 206 | 54.9 | 0.285 | 0.858, 0.648-1.136 | 0.546 | 0.915, 0.684-1.222 |
|  | TC | 154 | 36.2 |  | 141 | 37.6 | 0.671 | 1.064, 0.798-1.419 | 0.780 | 1.044，0.774-1.408 |
|  | TT | 22 | 5.2 |  | 28 | 7.5 | 0.179 | 1.482, 0.833-2.637 | 0.076 | 1.715, 0.945-3.109 |
|  | C | 654 | 76.8 |  | 553 | 73.7 | 0.161 | 1.177, 0.937-1.477 |  |  |
|  | T | 198 | 23.2 |  | 197 | 26.3 |  |  |  |  |
|  | rs1042098 |  |  |  |  |  | 0.934 |  | 0.914 |  |
|  | AA | 350 | 82.7 |  | 312 | 83.6 | 0.734 | 1.067, 0.735-1.549 | 0.757 | 1.063, 0.723-1.563 |
|  | GA | 69 | 16.3 |  | 58 | 15.5 | 0.769 | 0.945, 0.646-1.382 | 0.820 | 0.955, 0.644-1.416 |
|  | GG | 4 | 0.9 |  | 3 | 0.8 | 1.000 | 0.849, 0.189-3.820 | 0.728 | 0.757, 0.157-3.644 |
|  | A | 769 | 90.9 |  | 682 | 91.4 | 0.714 | 0.937, 0.662-1.326 |  |  |
|  | G | 77 | 9.1 |  | 64 | 8.6 |  |  |  |  |
|  | rs6347 |  |  |  |  |  | 0.674 |  | 0.719 |  |
|  | TT | 322 | 75.6 |  | 293 | 78.1 | 0.394 | 1.154, 0.830-1.605 | 0.499 | 1.125, 0.800-1.582 |
|  | TC | 99 | 23.2 |  | 79 | 21.1 | 0.460 | 0.882, 0.631-1.232 | 0.581 | 0.907, 0.642-1.282 |
|  | CC | 5 | 1.2 |  | 3 | 0.8 | 0.596 | 0.679, 0.161-2.861 | 0.573 | 0.645, 0.141-2.960 |
|  | T | 743 | 87.2 |  | 665 | 88.7 | 0.371 | 0.871, 0.644-1.179 |  |  |
|  | C | 109 | 12.8 |  | 85 | 11.3 |  |  |  |  |
| *TH* | rs10770140 |  |  |  |  |  | 0.631 |  | 0.989 |  |
|  | TT | 365 | 85.7 |  | 323 | 86.1 | 0.854 | 1.038, 0.696-1.547 | 0.763 | 1.065, 0.706-1.608 |
|  | TC | 59 | 13.8 |  | 52 | 13.9 | 0.994 | 1.001, 0.670-1.496 | 0.903 | 0.974, 0.644-1.475 |
|  | CC | 2 | 0.5 |  | 0 | 0.0 | 0.501 | 0.995, 0.989-1.002 | 0.999 | 0.000, 0.000- |
|  | T | 789 | 92.6 |  | 698 | 93.1 | 0.721 | 0.933, 0.637-1.366 |  |  |
|  | C | 63 | 7.4 |  | 52 | 6.9 |  |  |  |  |
|  | rs10770141 |  |  |  |  |  | 0.621 |  | 0.940 |  |
|  | GG | 369 | 86.6 |  | 328 | 87.5 | 0.722 | 1.078, 0.713-1.631 | 0.609 | 1.118, 0.729-1.715 |
|  | GA | 55 | 12.9 |  | 47 | 12.5 | 0.873 | 0.967, 0.637-1.466 | 0.744 | 0.931, 0.605-1.432 |
|  | AA | 2 | 0.5 |  | 0 | 0.0 | 0.501 | 0.995, 0.989-1.002 | 0.999 | 0.000, 0.000- |
|  | G | 793 | 93.1 |  | 703 | 93.7 | 0.597 | 0.899, 0.605-1.336 |  |  |
|  | A | 59 | 6.9 |  | 47 | 6.3 |  |  |  |  |
|  | rs3842727 |  |  |  |  |  | 1.000 |  | 0.909 |  |
|  | TT | 394 | 92.5 |  | 347 | 92.5 | 0.981 | 1.007, 0.594-1.705 | 0.589 | 1.163, 0.673-2.007 |
|  | TG | 31 | 7.3 |  | 28 | 7.5 | 0.918 | 1.028, 0.605-1.748 | 0.669 | 1.127, 0.651-1.953 |
|  | GG | 1 | 0.2 |  | 0 | 0.0 | 1.000 | 0.998, 0.993-1.002 | 1.000 | 0.000, 0.000- |
|  | T | 819 | 96.1 |  | 722 | 96.3 | 0.884 | 0.962, 0.576-1.608 |  |  |
|  | G | 33 | 3.9 |  | 28 | 3.7 |  |  |  |  |
|  | rs6356 |  |  |  |  |  | 0.360 |  | 0.537 |  |
|  | TT | 309 | 72.5 |  | 277 | 73.9 | 0.671 | 1.070, 0.782-1.464 | 0.973 | 1.006, 0.728-1.390 |
|  | TC | 102 | 23.9 |  | 91 | 24.3 | 0.915 | 1.018, 0.736-1.408 | 0.705 | 1.067, 0.763-1.492 |
|  | CC | 15 | 3.5 |  | 7 | 1.9 | 0.153 | 0.521, 0.210-1.292 | 0.280 | 0.599, 0.236-1.520 |
|  | T | 720 | 84.5 |  | 645 | 86.0 | 0.401 | 0.888, 0.673-1.172 |  |  |
|  | C | 132 | 15.5 |  | 105 | 14.0 |  |  |  |  |
| *DDC* | rs11575553 |  |  |  |  |  | 0.487 |  | 0.477 |  |
|  | GG | 329 | 77.2 |  | 300 | 80.0 | 0.341 | 1.179, 0.840-1.656 | 0.237 | 1.236, 0.870-1.756 |
|  | GA | 83 | 19.5 |  | 67 | 17.9 | 0.558 | 0.899, 0.629-1.284 | 0.333 | 0.833, 0.575-1.206 |
|  | AA | 14 | 3.3 |  | 8 | 2.1 | 0.319 | 0.641, 0.266-1.547 | 0.507 | 0.737, 0.300-1.813 |
|  | G | 741 | 87.0 |  | 667 | 88.9 | 0.230 | 0.831, 0.614-1.125 |  |  |
|  | A | 111 | 13.0 |  | 83 | 11.1 |  |  |  |  |
|  | rs12666409 |  |  |  |  |  | 0.560 |  | 0.701 |  |
|  | TT | 127 | 29.8 |  | 99 | 26.4 | 0.284 | 0.844, 0.620-1.151 | 0.403 | 0.872, 0.634-1.201 |
|  | TA | 204 | 47.9 |  | 187 | 49.9 | 0.576 | 1.082, 0.820-1.429 | 0.671 | 1.064, 0.798-1.419 |
|  | AA | 95 | 22.3 |  | 89 | 23.7 | 0.630 | 1.084, 0.780-1.508 | 0.696 | 1.071, 0.761-1.507 |
|  | T | 458 | 53.8 |  | 385 | 51.3 | 0.333 | 0.907, 0.745-1.105 |  |  |
|  | A | 394 | 46.2 |  | 365 | 48.7 |  |  |  |  |
| *DBH* | rs129882 |  |  |  |  |  | 0.631 |  | 0.568 |  |
|  | CC | 174 | 40.8 |  | 163 | 43.5 | 0.453 | 1.114, 0.841-1.475 | 0.306 | 1.165, 0.869-1.561 |
|  | TC | 195 | 45.8 |  | 159 | 42.4 | 0.337 | 0.872, 0.659-1.154 | 0.334 | 0.867, 0.649-1.158 |
|  | TT | 57 | 13.4 |  | 53 | 14.1 | 0.757 | 1.066, 0.712-1.594 | 0.946 | 1.014, 0.668-1.541 |
|  | C | 543 | 63.7 |  | 485 | 64.7 | 0.697 | 0.960, 0.782-1.178 |  |  |
|  | T | 309 | 36.3 |  | 265 | 35.3 |  |  |  |  |
|  | rs129915 |  |  |  |  |  | 0.903 |  | 0.772 |  |
|  | AA | 131 | 30.8 |  | 110 | 29.3 | 0.622 | 0.935, 0.690-1.265 | 0.476 | 0.892, 0.652-1.221 |
|  | GA | 215 | 50.5 |  | 192 | 51.2 | 0.837 | 1.030, 0.780-1.359 | 0.582 | 1.084, 0.813-1.445 |
|  | GG | 80 | 18.8 |  | 73 | 19.5 | 0.805 | 1.045, 0.735-1.488 | 0.894 | 1.025, 0.711-1.479 |
|  | A | 477 | 56.0 |  | 412 | 54.9 | 0.672 | 1.044, 0.857-1.271 |  |  |
|  | G | 375 | 44.0 |  | 338 | 45.1 |  |  |  |  |
|  | rs1611114 |  |  |  |  |  | 0.828 |  | 0.870 |  |
|  | TT | 186 | 43.7 |  | 156 | 41.6 | 0.556 | 0.919, 0.694-1.217 | 0.613 | 0.928, 0.694-1.241 |
|  | TC | 189 | 44.4 |  | 171 | 45.6 | 0.726 | 1.051, 0.795-1.389 | 0.624 | 1.075, 0.806-1.434 |
|  | CC | 51 | 12.0 |  | 48 | 12.8 | 0.722 | 1.079, 0.708-1.645 | 0.986 | 1.004, 0.646-1.560 |
|  | T | 561 | 65.8 |  | 483 | 64.4 | 0.545 | 1.066, 0.867-1.309 |  |  |
|  | C | 291 | 34.2 |  | 267 | 35.6 |  |  |  |  |
|  | rs5320 |  |  |  |  |  | 0.119 |  | 0.230 |  |
|  | GG | 305 | 71.6 |  | 288 | 76.8 | 0.094 | 1.313, 0.954-1.807 | 0.182 | 1.252, 0.900-1.742 |
|  | GA | 114 | 26.8 |  | 78 | 20.8 | 0.049 | 0.719, 0.517-0.999 | 0.108 | 0.757, 0.539-1.063 |
|  | AA | 7 | 1.6 |  | 9 | 2.4 | 0.445 | 1.472, 0.543-3.992 | 0.471 | 1.461, 0.521-4.096 |
|  | G | 724 | 85.0 |  | 654 | 87.2 | 0.200 | 0.830, 0.624-1.104 |  |  |
|  | A | 128 | 15.0 |  | 96 | 12.8 |  |  |  |  |

*P*^a^: Genotypic and allele frequency difference assessment by chi-square test.

*P*^b^: Genotypic and allele frequency difference assessment by binary logistic regression.

**Table S7** Results of association analysis between SNPs and phenotype of DTFUD

| Gene | Variable/ID | DTFUD≤1month(n=425) | |  | DTFUD>1month(n=376) | | *P*^a^ | OR, 95% CI | *P*^b^ | Exp(B), 95% CI |
| --- | --- | --- | --- | --- | --- | --- | --- | --- | --- | --- |
|  |  | Number | Percent |  | Number | Percent |  |  |  |  |
| *SLC6A3* | rs10064525 |  |  |  |  |  | 0.745 |  | 0.727 |  |
|  | TT | 376 | 88.5 |  | 339 | 90.2 | 0.441 | 0.838,0.533-1.316 | 0.470 | 0.844,0.532-1.339 |
|  | TG | 48 | 11.3 |  | 36 | 9.6 | 0.428 | 1.202,0.762-1.898 | 0.443 | 1.200,0.753-1.912 |
|  | GG | 1 | 0.2 |  | 1 | 0.3 | 1.000 | 0.884,0.055-14.286 | 0.814 | 0.713,0.043-11.905 |
|  | T | 800 | 94.1 |  | 714 | 94.9 | 0.467 | 0.852, 0.552-1.314 |  |  |
|  | G | 50 | 5.9 |  | 38 | 5.1 |  |  |  |  |
|  | rs27072 |  |  |  |  |  | 0.148 |  | 0.157 |  |
|  | CC | 233 | 54.8 |  | 223 | 59.3 | 0.201 | 0.833,0.629-1.103 | 0.238 | 0.841,0.631-1.121 |
|  | TC | 169 | 39.8 |  | 126 | 33.5 | 0.067 | 1.311,0.981-1.748 | 0.078 | 1.307,0.971-1.761 |
|  | TT | 23 | 5.4 |  | 27 | 7.2 | 0.302 | 0.739,0.416-1.314 | 0.278 | 0.722,0.400-1.300 |
|  | C | 635 | 74.7 |  | 572 | 76.1 | 0.529 | 0.929, 0.740-1.167 |  |  |
|  | T | 215 | 25.3 |  | 180 | 23.9 |  |  |  |  |
|  | rs1042098 |  |  |  |  |  | 0.178 |  | 0.209 |  |
|  | AA | 361 | 85.3 |  | 301 | 80.7 | 0.080 | 1.393,0.959-2.020 | 0.082 | 1.401,0.958-2.049 |
|  | GA | 58 | 13.7 |  | 69 | 18.5 | 0.066 | 0.700,0.478-1.025 | 0.077 | 0.704,0.477-1.039 |
|  | GG | 4 | 0.9 |  | 3 | 0.8 | 1.000 | 1.178,0.262-5.291 | 0.978 | 1.022,0.222-4.704 |
|  | A | 780 | 92.2 |  | 671 | 89.9 | 0.114 | 1.321, 0.934-1.868 |  |  |
|  | G | 66 | 7.8 |  | 75 | 10.1 |  |  |  |  |
|  | rs6347 |  |  |  |  |  | 0.526 |  | 0.662 |  |
|  | TT | 333 | 78.4 |  | 282 | 75.0 | 0.262 | 1.206,0.869-1.675 | 0.364 | 1.168,0.835-1.634 |
|  | TC | 88 | 20.7 |  | 90 | 23.9 | 0.272 | 0.829,0.595-1.159 | 0.375 | 0.858,0.609-1.205 |
|  | CC | 4 | 0.9 |  | 4 | 1.1 | 1.000 | 0.883,0.219-3.559 | 0.885 | 0.900,0.216-3.745 |
|  | T | 754 | 88.7 |  | 654 | 87.0 | 0.287 | 1.177, 0.872-1.589 |  |  |
|  | C | 96 | 11.3 |  | 98 | 13.0 |  |  |  |  |
| *TH* | rs10770140 |  |  |  |  |  | 0.959 |  | 0.972 |  |
|  | TT | 366 | 86.1 |  | 322 | 85.6 | 0.846 | 1.041,0.698-1.550 | 0.831 | 1.045,0.696-1.569 |
|  | TC | 58 | 13.6 |  | 53 | 14.1 | 0.854 | 0.963,0.645-1.439 | 0.845 | 0.959,0.637-1.447 |
|  | CC | 1 | 0.2 |  | 1 | 0.3 | 1.000 | 0.884,0.055-14.286 | 0.894 | 0.827,0.051-13.333 |
|  | T | 790 | 92.9 |  | 697 | 92.7 | 0.844 | 1.039, 0.711-1.519 |  |  |
|  | C | 60 | 7.1 |  | 55 | 7.3 |  |  |  |  |
|  | rs10770141 |  |  |  |  |  | 1.000 |  | 0.984 |  |
|  | GG | 370 | 87.1 |  | 327 | 87.0 | 0.970 | 1.008,0.667-1.522 | 0.888 | 1.031,0.676-1.572 |
|  | GA | 54 | 12.7 |  | 48 | 12.8 | 0.980 | 0.995,0.656-1.508 | 0.903 | 0.974,0.636-1.490 |
|  | AA | 1 | 0.2 |  | 1 | 0.3 | 1.000 | 0.884,0.055-14.286 | 0.894 | 0.827,0.051-13.333 |
|  | G | 794 | 93.4 |  | 702 | 93.4 | 0.844 | 1.039, 0.711-1.519 |  |  |
|  | A | 56 | 6.6 |  | 50 | 6.6 |  |  |  |  |
|  | rs3842727 |  |  |  |  |  | 0.456 |  | 0.892 |  |
|  | TT | 391 | 92.0 |  | 350 | 93.1 | 0.560 | 0.854,0.503-1.451 | 0.734 | 0.909,0.529-1.567 |
|  | TG | 34 | 8.0 |  | 25 | 6.6 | 0.465 | 1.221,0.714-2.088 | 0.623 | 1.148,0.663-1.988 |
|  | GG | 0 | 0.0 |  | 1 | 0.3 | 0.469 | 0.997,0.992-1.003 | 1.000 | 1941262790, 0.000- |
|  | T | 816 | 96.0 |  | 725 | 96.4 | 0.669 | 0.894, 0.534-1.496 |  |  |
|  | G | 34 | 4.0 |  | 27 | 3.6 |  |  |  |  |
|  | rs6356 |  |  |  |  |  | 0.984 |  | 0.997 |  |
|  | TT | 310 | 72.9 |  | 276 | 73.4 | 0.883 | 0.977,0.714-1.335 | 0.935 | 0.987,0.716-1.359 |
|  | TC | 103 | 24.2 |  | 90 | 23.9 | 0.921 | 1.016,0.735-1.406 | 0.937 | 1.013,0.727-1.412 |
|  | CC | 12 | 2.8 |  | 10 | 2.7 | 0.887 | 1.064,0.454-2.488 | 0.988 | 1.007,0.418-2.427 |
|  | T | 723 | 85.1 |  | 642 | 85.4 | 0.860 | 0.975, 0.740-1.286 |  |  |
|  | C | 127 | 14.9 |  | 110 | 14.6 |  |  |  |  |
| *DDC* | rs11575553 |  |  |  |  |  | 0.601 |  | 0.548 |  |
|  | GG | 332 | 78.1 |  | 297 | 79.0 | 0.764 | 0.949,0.677-1.332 | 0.791 | 0.954,0.675-1.349 |
|  | GA | 79 | 18.6 |  | 71 | 18.9 | 0.915 | 0.980,0.687-1.401 | 0.858 | 0.967,0.672-1.393 |
|  | AA | 14 | 3.3 |  | 8 | 2.1 | 0.313 | 1.567,0.649-3.774 | 0.275 | 1.658,0.669-4.115 |
|  | G | 743 | 87.4 |  | 665 | 88.4 | 0.533 | 0.908, 0.672-1.228 |  |  |
|  | A | 107 | 12.6 |  | 87 | 11.6 |  |  |  |  |
|  | rs12666409 |  |  |  |  |  | 0.811 |  | 0.826 |  |
|  | TT | 116 | 27.3 |  | 110 | 29.3 | 0.538 | 0.907,0.667-1.236 | 0.580 | 0.915,0.667-1.255 |
|  | TA | 209 | 49.2 |  | 182 | 48.4 | 0.827 | 1.031,0.781-1.361 | 0.904 | 1.017,0.766-1.351 |
|  | AA | 100 | 23.5 |  | 84 | 22.3 | 0.690 | 1.069,0.769-1.488 | 0.653 | 1.081,0.770-1.515 |
|  | T | 441 | 51.9 |  | 402 | 53.5 | 0.529 | 1.065, 0.875-1.297 |  |  |
|  | A | 409 | 48.1 |  | 350 | 46.5 |  |  |  |  |
| *DBH* | rs129882 |  |  |  |  |  | 0.633 |  | 0.704 |  |
|  | CC | 177 | 41.6 |  | 160 | 42.6 | 0.795 | 0.963,0.727-1.276 | 0.944 | 0.990,0.741-1.321 |
|  | TC | 185 | 43.5 |  | 169 | 44.9 | 0.687 | 0.944,0.714-1.248 | 0.622 | 0.930,0.699-1.239 |
|  | TT | 63 | 14.8 |  | 47 | 12.5 | 0.340 | 1.218,0.812-1.828 | 0.418 | 1.188,0.784-1.795 |
|  | C | 539 | 63.4 |  | 489 | 65.0 | 0.501 | 0.932, 0.760-1.144 |  |  |
|  | T | 311 | 36.6 |  | 263 | 35.0 |  |  |  |  |
|  | rs129915 |  |  |  |  |  | 0.814 |  | 0.819 |  |
|  | AA | 124 | 29.2 |  | 117 | 31.1 | 0.550 | 0.912,0.674-1.235 | 0.531 | 0.906,0.665-1.235 |
|  | GA | 220 | 51.8 |  | 187 | 49.7 | 0.566 | 1.085,0.822-1.433 | 0.636 | 1.071,0.806-1.422 |
|  | GG | 81 | 19.1 |  | 72 | 19.1 | 0.974 | 0.994,0.698-1.414 | 0.897 | 0.976,0.679-1.403 |
|  | A | 468 | 55.1 |  | 421 | 56.0 | 0.710 | 0.963, 0.791-1.174 |  |  |
|  | G | 382 | 44.9 |  | 331 | 44.0 |  |  |  |  |
|  | rs1611114 |  |  |  |  |  | 0.128 |  | 0.085 |  |
|  | TT | 186 | 43.8 |  | 156 | 41.5 | 0.516 | 1.098,0.829-1.453 | 0.428 | 1.124,0.842-1.497 |
|  | TC | 179 | 42.1 |  | 181 | 48.1 | 0.087 | 0.784,0.593-1.036 | 0.056 | 0.756,0.569-1.007 |
|  | CC | 60 | 14.1 |  | 39 | 10.4 | 0.108 | 1.420,0.924-2.183 | 0.087 | 1.471,0.945-2.288 |
|  | T | 551 | 64.8 |  | 493 | 65.6 | 0.758 | 0.968, 0.788-1.190 |  |  |
|  | C | 299 | 35.2 |  | 259 | 34.4 |  |  |  |  |
|  | rs5320 |  |  |  |  |  | 0.165 |  | 0.163 |  |
|  | GG | 316 | 74.4 |  | 277 | 73.7 | 0.826 | 1.036,0.755-1.422 | 0.851 | 1.032,0.746-1.427 |
|  | GA | 97 | 22.8 |  | 95 | 25.3 | 0.419 | 0.875,0.632-1.211 | 0.423 | 0.873,0.626-1.218 |
|  | AA | 12 | 2.8 |  | 4 | 1.1 | 0.076 | 2.703,0.864-8.475 | 0.073 | 2.915,0.903-9.346 |
|  | G | 729 | 85.8 |  | 649 | 86.3 | 0.756 | 0.956, 0.720-1.269 |  |  |
|  | A | 121 | 14.2 |  | 103 | 13.7 |  |  |  |  |

*P*^a^: Genotypic and allele frequency difference assessment by chi-square test.

*P*^b^: Genotypic and allele frequency difference assessment by binary logistic regression.

**Table S8** Transcription factor binding sites of rs1611114

| **SNP** | **Allele** | **Position** | **Prediction Strand** | **Forward Sequence** | **Core Match Score** | **Matrix Match Score** | **Transfac Matrix ID** |
| --- | --- | --- | --- | --- | --- | --- | --- |
| rs1611114 | T | 10 | + | tttCACGTttgt | 0.8 | 0.796 | V$AREB6_02 |
| rs1611114 | C | 12 | + | aatttcACGTTcgtgc | 0.792 | 0.804 | V$ARNT_01 |
| rs1611114 | C | 12 | - | aatttCACGTtcgtgc | 1 | 0.897 | V$ARNT_01 |
| rs1611114 | T | 12 | + | aatttcACGTTtgtgc | 0.792 | 0.803 | V$ARNT_01 |
| rs1611114 | T | 12 | - | aatttCACGTttgtgc | 1 | 0.896 | V$ARNT_01 |
| rs1611114 | C | 8 | + | TCACGttc | 0.8 | 0.677 | V$ATF6_01 |
| rs1611114 | C | 8 | - | tcaCGTTC | 0.6 | 0.645 | V$ATF6_01 |
| rs1611114 | T | 8 | + | TCACGttt | 0.8 | 0.677 | V$ATF6_01 |
| rs1611114 | T | 8 | - | tcaCGTTT | 0.6 | 0.645 | V$ATF6_01 |
| rs1611114 | C | 8 | + | tcaCGTTC | 0.881 | 0.855 | V$BRCA_01 |
| rs1611114 | T | 8 | + | tcaCGTTT | 0.889 | 0.862 | V$BRCA_01 |
| rs1611114 | C | 15 | - | CCAAAtttcacgttc | 0.744 | 0.57 | V$CDPCR3_01 |
| rs1611114 | T | 15 | - | CCAAAtttcacgttt | 0.744 | 0.57 | V$CDPCR3_01 |
| rs1611114 | C | 5 | - | cgttcgtGCAAAga | 0.977 | 0.898 | V$CEBPA_01 |
| rs1611114 | T | 5 | + | cgTTTGTgcaaaga | 0.906 | 0.915 | V$CEBPA_01 |
| rs1611114 | T | 5 | - | cgtttgtGCAAAga | 0.977 | 0.974 | V$CEBPA_01 |
| rs1611114 | C | 12 | + | aATTTCacgttc | 0.986 | 0.854 | V$CEBPDELTA_Q6 |
| rs1611114 | T | 12 | + | aATTTCacgttt | 0.986 | 0.862 | V$CEBPDELTA_Q6 |
| rs1611114 | C | 12 | - | aatttCACGTtcg | 0.659 | 0.699 | V$CEBPGAMMA_Q6 |
| rs1611114 | T | 12 | - | aatttCACGTttg | 0.659 | 0.699 | V$CEBPGAMMA_Q6 |
| rs1611114 | C | 5 | + | cgttcgTGCAAa | 0.997 | 0.96 | V$CEBP_Q3 |
| rs1611114 | C | 11 | - | aTTTCAcgttcg | 0.987 | 0.926 | V$CEBP_Q3 |
| rs1611114 | T | 5 | + | cgtttgTGCAAa | 0.997 | 0.989 | V$CEBP_Q3 |
| rs1611114 | T | 11 | - | aTTTCAcgtttg | 0.987 | 0.927 | V$CEBP_Q3 |
| rs1611114 | C | 20 | + | tttctccaaatTTCACgttcg | 0.812 | 0.643 | V$DR3_Q4 |
| rs1611114 | T | 20 | + | tttctccaaatTTCACgtttg | 0.812 | 0.645 | V$DR3_Q4 |
| rs1611114 | T | 8 | - | tcacgTTTGTgcaa | 0.925 | 0.784 | V$FAC1_01 |
| rs1611114 | T | 10 | - | tttcacgTTTGT | 0.79 | 0.74 | V$GATA4_Q3 |
| rs1611114 | C | 12 | - | aatttCACGTtcgt | 1 | 0.882 | V$HIF1_Q3 |
| rs1611114 | T | 12 | - | aatttCACGTttgt | 1 | 0.876 | V$HIF1_Q3 |
| rs1611114 | C | 2 | - | tcgtgcAAAGA | 0.908 | 0.835 | V$HNF3ALPHA_Q6 |
| rs1611114 | T | 2 | - | ttgtgcAAAGA | 0.908 | 0.862 | V$HNF3ALPHA_Q6 |
| rs1611114 | T | 12 | - | aatttcacGTTTG | 0.622 | 0.795 | V$IRF2_01 |
| rs1611114 | C | 2 | - | tcgtgCAAAGa | 1 | 0.869 | V$LEF1TCF1_Q4 |
| rs1611114 | T | 2 | - | ttgtgCAAAGa | 1 | 0.87 | V$LEF1TCF1_Q4 |
| rs1611114 | C | 11 | + | atttcACGTTc | 0.901 | 0.874 | V$MYB_Q3 |
| rs1611114 | T | 11 | + | atttcACGTTt | 0.901 | 0.872 | V$MYB_Q3 |
| rs1611114 | C | 15 | + | ccaaatttcacgTTCGTgcaaagacacag | 0.708 | 0.602 | V$MYOGNF1_01 |
| rs1611114 | C | 10 | - | tttcacgttcgtGCAAAgacacagtcatt | 0.819 | 0.533 | V$MYOGNF1_01 |
| rs1611114 | C | 28 | - | gctggcactttcTCCAAatttcacgttcg | 0.919 | 0.617 | V$MYOGNF1_01 |
| rs1611114 | T | 15 | + | ccaaatttcacgTTTGTgcaaagacacag | 0.738 | 0.613 | V$MYOGNF1_01 |
| rs1611114 | T | 13 | + | aaatttcacgttTGTGCaaagacacagtc | 0.638 | 0.526 | V$MYOGNF1_01 |
| rs1611114 | T | 10 | - | tttcacgtttgtGCAAAgacacagtcatt | 0.819 | 0.518 | V$MYOGNF1_01 |
| rs1611114 | T | 28 | - | gctggcactttcTCCAAatttcacgtttg | 0.919 | 0.632 | V$MYOGNF1_01 |
| rs1611114 | C | 10 | + | tttCACGTtc | 0.8 | 0.777 | V$NKX25_Q5 |
| rs1611114 | C | 1 | + | cgtGCAAAgacac | 0.774 | 0.783 | V$OCT1_03 |
| rs1611114 | T | 1 | + | tgtGCAAAgacac | 0.774 | 0.783 | V$OCT1_03 |
| rs1611114 | T | 2 | - | ttgTGCAAagacaca | 0.794 | 0.649 | V$OCT4_01 |
| rs1611114 | C | 1 | - | cGTGCAaagac | 0.736 | 0.751 | V$OCT_Q6 |
| rs1611114 | T | 1 | - | tGTGCAaagac | 0.736 | 0.761 | V$OCT_Q6 |
| rs1611114 | C | 13 | + | aaatTTCACgttcgtgcaa | 0.866 | 0.695 | V$PAX2_01 |
| rs1611114 | T | 13 | + | aaatTTCACgtttgtgcaa | 0.866 | 0.751 | V$PAX2_01 |
| rs1611114 | C | 16 | + | tccaaattTCACGttcgtgca | 1 | 0.843 | V$PAX3_B |
| rs1611114 | C | 9 | - | ttcacgttCGTGCaaagacac | 0.806 | 0.629 | V$PAX3_B |
| rs1611114 | T | 16 | + | tccaaattTCACGtttgtgca | 1 | 0.853 | V$PAX3_B |
| rs1611114 | C | 4 | - | gttcgtgcaaagacacagtcattccTTTCT | 0.943 | 0.544 | V$PAX4_04 |
| rs1611114 | T | 4 | - | gtttgtgcaaagacacagtcattccTTTCT | 0.943 | 0.54 | V$PAX4_04 |
| rs1611114 | T | 28 | - | gctggcactttctccaaatttcacgTTTGT | 0.927 | 0.577 | V$PAX4_04 |
| rs1611114 | C | 13 | + | aaattTCACGttcgtgcaaag | 1 | 0.626 | V$PAX6_01 |
| rs1611114 | C | 7 | + | cacgtTCGTGcaaagacacag | 0.653 | 0.508 | V$PAX6_01 |
| rs1611114 | C | 12 | - | aatttcacgttCGTGCaaaga | 0.728 | 0.489 | V$PAX6_01 |
| rs1611114 | C | 16 | - | tccaaatttcaCGTTCgtgca | 0.659 | 0.52 | V$PAX6_01 |
| rs1611114 | T | 13 | + | aaattTCACGtttgtgcaaag | 1 | 0.727 | V$PAX6_01 |
| rs1611114 | T | 7 | + | cacgtTTGTGcaaagacacag | 0.613 | 0.489 | V$PAX6_01 |
| rs1611114 | T | 16 | - | tccaaatttcaCGTTTgtgca | 0.665 | 0.523 | V$PAX6_01 |
| rs1611114 | C | 9 | + | ttcacgttCGTGCaa | 0.848 | 0.701 | V$PAX8_01 |
| rs1611114 | C | 10 | - | ttTCACGttcgtgca | 1 | 0.793 | V$PAX8_01 |
| rs1611114 | T | 10 | - | ttTCACGtttgtgca | 1 | 0.797 | V$PAX8_01 |
| rs1611114 | C | 26 | + | tggcactttctcCAAATttcacgttcgtg | 0.735 | 0.601 | V$PLZF_02 |
| rs1611114 | C | 9 | + | ttcacgttcgtgCAAAGacacagtcattc | 0.756 | 0.619 | V$PLZF_02 |
| rs1611114 | C | 23 | - | cactttctccaaATTTCacgttcgtgcaa | 0.735 | 0.698 | V$PLZF_02 |
| rs1611114 | T | 26 | + | tggcactttctcCAAATttcacgtttgtg | 0.735 | 0.602 | V$PLZF_02 |
| rs1611114 | T | 9 | + | ttcacgtttgtgCAAAGacacagtcattc | 0.756 | 0.618 | V$PLZF_02 |
| rs1611114 | T | 23 | - | cactttctccaaATTTCacgtttgtgcaa | 0.735 | 0.696 | V$PLZF_02 |
| rs1611114 | C | 8 | + | TCACGttcgt | 0.565 | 0.55 | V$POU3F2_02 |
| rs1611114 | T | 8 | + | TCACGtttgt | 0.565 | 0.55 | V$POU3F2_02 |
| rs1611114 | T | 2 | + | TTGTGcaaag | 0.782 | 0.654 | V$POU3F2_02 |
| rs1611114 | C | 2 | + | tcgtgcaaagaCACAGtcat | 0.898 | 0.542 | V$PPARA_01 |
| rs1611114 | T | 2 | + | ttgtgcaaagaCACAGtcat | 0.898 | 0.546 | V$PPARA_01 |
| rs1611114 | T | 8 | - | tcacGTTTGtgcaaagacac | 0.75 | 0.614 | V$PPARA_01 |
| rs1611114 | C | 5 | + | cgttcGTGCAaagacacagtcat | 0.639 | 0.496 | V$PPARG_02 |
| rs1611114 | C | 21 | - | ctttctccaaattTCACGttcgt | 0.65 | 0.436 | V$PPARG_02 |
| rs1611114 | T | 5 | + | cgtttGTGCAaagacacagtcat | 0.639 | 0.496 | V$PPARG_02 |
| rs1611114 | T | 21 | - | ctttctccaaattTCACGtttgt | 0.65 | 0.429 | V$PPARG_02 |
| rs1611114 | T | 22 | - | actttctccaaatTTCACgtttg | 0.522 | 0.434 | V$PPARG_02 |
| rs1611114 | C | 10 | + | tttCACGTtcg | 1 | 0.738 | V$SREBP1_01 |
| rs1611114 | C | 9 | - | ttcACGTTcgt | 0.749 | 0.669 | V$SREBP1_01 |
| rs1611114 | T | 10 | + | tttCACGTttg | 1 | 0.735 | V$SREBP1_01 |
| rs1611114 | T | 9 | - | ttcACGTTtgt | 0.749 | 0.673 | V$SREBP1_01 |
| rs1611114 | C | 12 | + | aattTCACGttcgtg | 0.993 | 0.878 | V$SREBP_Q6 |
| rs1611114 | T | 12 | + | aattTCACGtttgtg | 0.993 | 0.868 | V$SREBP_Q6 |
| rs1611114 | C | 9 | + | ttcacgttcGTGCAaagacac | 0.48 | 0.674 | V$STAT1_01 |
| rs1611114 | C | 9 | - | ttcacgtTCGTGcaaagacac | 0.671 | 0.687 | V$STAT1_01 |
| rs1611114 | C | 8 | + | tcacgTTCGTgca | 0.903 | 0.824 | V$STAT_Q6 |
| rs1611114 | T | 3 | + | TTTGTgc | 0.917 | 0.891 | V$TBP_Q6 |
| rs1611114 | C | 9 | + | TTCACgttcgtgc | 0.778 | 0.778 | V$TCF11_01 |
| rs1611114 | T | 9 | + | TTCACgtttgtgc | 0.778 | 0.781 | V$TCF11_01 |
| rs1611114 | C | 7 | + | cacgttCGTGCaa | 0.921 | 0.799 | V$ZF5_B |
| rs1611114 | C | 6 | - | acGTTCGtgcaaa | 0.807 | 0.682 | V$ZF5_B |

**Table S9** Transcription factor binding sites of rs10770140

| **SNP** | **Allele** | **Position** | **Prediction Strand** | **Forward Sequence** | **Core Match Score** | **Matrix Match Score** | **Transfac Matrix ID** |
| --- | --- | --- | --- | --- | --- | --- | --- |
| rs10770140 | C | 5 | + | GCCCCgtgg | 0.999 | 0.98 | V$AP2ALPHA_01 |
| rs10770140 | T | 5 | + | GCCCTgtgg | 0.998 | 0.979 | V$AP2ALPHA_01 |
| rs10770140 | C | 6 | + | cgCCCCGtgggg | 0.944 | 0.887 | V$AP2_Q6 |
| rs10770140 | C | 5 | + | gcCCCGTggggt | 0.922 | 0.799 | V$AP2_Q6 |
| rs10770140 | C | 4 | - | ccccgTGGGGtc | 0.934 | 0.859 | V$AP2_Q6 |
| rs10770140 | C | 5 | - | gccccGTGGGgt | 0.968 | 0.845 | V$AP2_Q6 |
| rs10770140 | T | 6 | + | cgCCCTGtgggg | 0.904 | 0.86 | V$AP2_Q6 |
| rs10770140 | T | 5 | - | gccctGTGGGgt | 0.968 | 0.846 | V$AP2_Q6 |
| rs10770140 | C | 5 | - | gCCCCGtggggtc | 0.737 | 0.805 | V$AP2_Q6_01 |
| rs10770140 | C | 6 | - | cGCCCCgtggggt | 0.889 | 0.789 | V$AP2_Q6_01 |
| rs10770140 | T | 5 | - | gCCCTGtggggtc | 0.751 | 0.814 | V$AP2_Q6_01 |
| rs10770140 | C | 8 | + | ctcgccCCGTGgggtc | 0.83 | 0.823 | V$ARNT_01 |
| rs10770140 | C | 8 | - | ctcgcCCCGTggggtc | 0.792 | 0.787 | V$ARNT_01 |
| rs10770140 | C | 4 | - | cccCGTGG | 0.6 | 0.593 | V$ATF6_01 |
| rs10770140 | T | 11 | + | aagctcgcccTGTGG | 0.744 | 0.53 | V$CDPCR3_01 |
| rs10770140 | C | 3 | - | cccgtggggtCCAGA | 0.957 | 0.863 | V$CP2_02 |
| rs10770140 | T | 3 | - | cctgtggggtCCAGA | 0.957 | 0.86 | V$CP2_02 |
| rs10770140 | C | 18 | + | gcttcccaagcTCGCCccgtg | 0.821 | 0.726 | V$DR3_Q4 |
| rs10770140 | T | 18 | + | gcttcccaagcTCGCCctgtg | 0.821 | 0.728 | V$DR3_Q4 |
| rs10770140 | T | 11 | - | aAGCTCgccctgtggg | 0.721 | 0.656 | V$GRE_C |
| rs10770140 | T | 8 | + | ctcgcccTGTGGggtc | 0.817 | 0.72 | V$HAND1E47_01 |
| rs10770140 | C | 1 | - | CGTGG | 1 | 1 | V$KID3_01 |
| rs10770140 | T | 1 | - | TGTGG | 0.994 | 0.994 | V$KID3_01 |
| rs10770140 | C | 8 | - | ctCGCCCc | 0.887 | 0.87 | V$MAZ_Q6 |
| rs10770140 | T | 8 | - | ctCGCCCt | 0.887 | 0.84 | V$MAZ_Q6 |
| rs10770140 | C | 20 | + | cggcttcccaagCTCGCcccgtggggtcc | 0.691 | 0.533 | V$MYOGNF1_01 |
| rs10770140 | C | 12 | + | caagctcgccccGTGGGgtccagaatgtc | 0.752 | 0.502 | V$MYOGNF1_01 |
| rs10770140 | C | 26 | - | ttgcagcggcttCCCAAgctcgccccgtg | 0.899 | 0.651 | V$MYOGNF1_01 |
| rs10770140 | T | 20 | + | cggcttcccaagCTCGCcctgtggggtcc | 0.691 | 0.533 | V$MYOGNF1_01 |
| rs10770140 | T | 12 | + | caagctcgccctGTGGGgtccagaatgtc | 0.752 | 0.527 | V$MYOGNF1_01 |
| rs10770140 | T | 26 | - | ttgcagcggcttCCCAAgctcgccctgtg | 0.899 | 0.658 | V$MYOGNF1_01 |
| rs10770140 | C | 9 | - | gctcgcccCGTGGggtccaga | 0.815 | 0.634 | V$PAX3_B |
| rs10770140 | C | 20 | + | cggcttccCAAGCtcgccccgtggggtc | 0.802 | 0.604 | V$PAX5_01 |
| rs10770140 | C | 5 | - | gccccgtggggtccaGAATGtcccagga | 0.812 | 0.688 | V$PAX5_01 |
| rs10770140 | T | 20 | + | cggcttccCAAGCtcgccctgtggggtc | 0.802 | 0.597 | V$PAX5_01 |
| rs10770140 | T | 5 | - | gccctgtggggtccaGAATGtcccagga | 0.812 | 0.675 | V$PAX5_01 |
| rs10770140 | C | 9 | + | gctcgcccCGTGGgg | 0.871 | 0.722 | V$PAX8_01 |
| rs10770140 | C | 15 | - | tcCCAAGctcgcccc | 0.76 | 0.711 | V$PAX8_01 |
| rs10770140 | T | 15 | - | tcCCAAGctcgccct | 0.76 | 0.707 | V$PAX8_01 |
| rs10770140 | C | 4 | - | ccccgtGGGGTccag | 0.88 | 0.797 | V$PEBP_Q6 |
| rs10770140 | T | 4 | - | ccctgtGGGGTccag | 0.88 | 0.813 | V$PEBP_Q6 |
| rs10770140 | T | 6 | - | cgccctGTGGGgtcc | 0.833 | 0.81 | V$PEBP_Q6 |
| rs10770140 | C | 20 | - | cggcttcccaagcTCGCCccgtg | 0.731 | 0.408 | V$PPARG_02 |
| rs10770140 | T | 20 | - | cggcttcccaagcTCGCCctgtg | 0.731 | 0.41 | V$PPARG_02 |
| rs10770140 | C | 12 | - | caagctcGCCCCgtg | 0.826 | 0.77 | V$SPZ1_01 |
| rs10770140 | C | 14 | - | cccaagcTCGCCccg | 0.768 | 0.728 | V$SPZ1_01 |
| rs10770140 | T | 14 | - | cccaagcTCGCCctg | 0.768 | 0.727 | V$SPZ1_01 |
| rs10770140 | C | 6 | + | cgcCCCGTggg | 0.749 | 0.634 | V$SREBP1_01 |
| rs10770140 | C | 5 | - | gccCCGTGggg | 0.749 | 0.629 | V$SREBP1_01 |
| rs10770140 | C | 11 | - | aagctcgccCCGTGg | 0.891 | 0.756 | V$SZF11_01 |
| rs10770140 | T | 13 | - | ccaagctcgCCCTGt | 1 | 0.658 | V$SZF11_01 |
| rs10770140 | C | 6 | + | cgccCCGTGggg | 0.855 | 0.818 | V$USF_Q6_01 |
| rs10770140 | C | 6 | - | cgcCCCGTgggg | 0.805 | 0.801 | V$USF_Q6_01 |
| rs10770140 | T | 5 | + | GCCCTgtgg | 0.791 | 0.772 | V$YY1_Q6 |
| rs10770140 | C | 12 | + | caagctCGCCCcg | 0.918 | 0.743 | V$ZF5_B |
| rs10770140 | C | 11 | - | aaGCTCGccccgt | 0.888 | 0.873 | V$ZF5_B |
| rs10770140 | T | 12 | + | caagctCGCCCtg | 0.918 | 0.838 | V$ZF5_B |
| rs10770140 | T | 2 | - | ctGTGGGgtccag | 0.737 | 0.657 | V$ZF5_B |
| rs10770140 | T | 11 | - | aaGCTCGccctgt | 0.888 | 0.861 | V$ZF5_B |

**Table S10** Transcription factor binding sites of rs10770141

| **SNP** | **Allele** | **Position** | **Prediction Strand** | **Forward Sequence** | **Core Match Score** | **Matrix Match Score** | **Transfac Matrix ID** |
| --- | --- | --- | --- | --- | --- | --- | --- |
| rs10770141 | G | 2 | + | TGAAGagc | 0.8 | 0.635 | V$ATF6_01 |
| rs10770141 | G | 8 | + | cttCTTTG | 0.838 | 0.825 | V$BRCA_01 |
| rs10770141 | G | 5 | + | cttTGAAGagca | 0.8 | 0.727 | V$GABP_B |
| rs10770141 | G | 6 | + | TCTTTgaagag | 0.908 | 0.816 | V$HNF3ALPHA_Q6 |
| rs10770141 | A | 5 | - | cttTAAAGagcac | 0.91 | 0.785 | V$HNF4ALPHA_Q6 |
| rs10770141 | A | 6 | + | tCTTTAaagag | 0.821 | 0.793 | V$LEF1TCF1_Q4 |
| rs10770141 | A | 7 | - | ttcttTAAAGa | 0.821 | 0.793 | V$LEF1TCF1_Q4 |
| rs10770141 | G | 6 | + | tCTTTGaagag | 1 | 0.938 | V$LEF1TCF1_Q4 |
| rs10770141 | G | 7 | - | ttCTTTGaag | 1 | 0.982 | V$LEF1_Q2_01 |
| rs10770141 | A | 27 | + | cctcggggtcctGTGGCcccttctttaaa | 0.852 | 0.516 | V$MYOGNF1_01 |
| rs10770141 | G | 27 | + | cctcggggtcctGTGGCcccttctttgaa | 0.852 | 0.512 | V$MYOGNF1_01 |
| rs10770141 | A | 6 | + | tctTTAAAgagca | 0.916 | 0.913 | V$OCT1_03 |
| rs10770141 | A | 9 | - | ccttcTTTAAaga | 0.916 | 0.913 | V$OCT1_03 |
| rs10770141 | G | 13 | - | ggccccttctTTGAAgagc | 0.845 | 0.657 | V$PAX2_01 |
| rs10770141 | A | 4 | + | tttaaagaGCACGcacgtccc | 0.806 | 0.644 | V$PAX3_B |
| rs10770141 | G | 4 | + | tttgaagaGCACGcacgtccc | 0.806 | 0.643 | V$PAX3_B |
| rs10770141 | A | 3 | + | ttaaagagCACGCacgtccctcagtgct | 0.802 | 0.664 | V$PAX5_01 |
| rs10770141 | G | 3 | + | ttgaagagCACGCacgtccctcagtgct | 0.802 | 0.667 | V$PAX5_01 |
| rs10770141 | G | 20 | - | gtcctgtggccccttCTTTGaagagcac | 0.964 | 0.721 | V$PAX5_01 |
| rs10770141 | A | 1 | + | aaagaGCACGcacgtccctca | 0.728 | 0.492 | V$PAX6_01 |
| rs10770141 | A | 16 | - | tgtggccccttCTTTAaagag | 0.762 | 0.508 | V$PAX6_01 |
| rs10770141 | G | 1 | + | gaagaGCACGcacgtccctca | 0.728 | 0.49 | V$PAX6_01 |
| rs10770141 | G | 12 | + | gccccttcTTTGAag | 0.841 | 0.703 | V$PAX8_01 |
| rs10770141 | A | 14 | + | tggccccttcttTAAAGagcacgcacgtc | 1 | 0.666 | V$PLZF_02 |
| rs10770141 | A | 17 | - | ctgtggccccttCTTTAaagagcacgcac | 1 | 0.711 | V$PLZF_02 |
| rs10770141 | G | 14 | + | tggccccttcttTGAAGagcacgcacgtc | 0.756 | 0.543 | V$PLZF_02 |
| rs10770141 | G | 17 | - | ctgtggccccttCTTTGaagagcacgcac | 0.756 | 0.588 | V$PLZF_02 |
| rs10770141 | A | 7 | + | TTCTTtaaag | 0.565 | 0.55 | V$POU3F2_02 |
| rs10770141 | G | 7 | + | TTCTTtgaag | 0.565 | 0.55 | V$POU3F2_02 |
| rs10770141 | A | 1 | - | aaagagcacgcacGTCCCtcagt | 0.487 | 0.456 | V$PPARG_02 |
| rs10770141 | G | 9 | + | ccttcTTTGAagagcacgcacgt | 0.437 | 0.472 | V$PPARG_02 |
| rs10770141 | G | 13 | + | ggccccttcTTTGAagagcac | 0.431 | 0.679 | V$STAT1_01 |
| rs10770141 | G | 13 | - | ggcccctTCTTTgaagagcac | 0.431 | 0.688 | V$STAT1_01 |
| rs10770141 | A | 12 | + | gccccTTCTTtaa | 0.993 | 0.811 | V$STAT_Q6 |
| rs10770141 | G | 12 | + | gccccTTCTTtga | 0.993 | 0.902 | V$STAT_Q6 |
| rs10770141 | G | 6 | - | tctTTGAAgagca | 0.888 | 0.863 | V$STAT_Q6 |
| rs10770141 | G | 3 | + | tTGAAGagcacgcac | 0.8 | 0.553 | V$TAXCREB_02 |
| rs10770141 | A | 4 | + | TTTAAag | 0.821 | 0.824 | V$TBP_Q6 |
| rs10770141 | A | 5 | - | ctTTAAA | 0.821 | 0.824 | V$TBP_Q6 |
| rs10770141 | A | 1 | + | aaagagCACGCac | 0.849 | 0.807 | V$ZF5_B |
| rs10770141 | G | 1 | + | gaagagCACGCac | 0.849 | 0.807 | V$ZF5_B |
