# Supplementary material for: Association studies of dopamine synthesis and metabolism genes with multiple phenotypes of heroin dependence
Source: BMC Med Genet. 2020 Jul 31;21:157. doi: 10.1186/s12881-020-01092-0 (PMC7393710; doi:10.1186/s12881-020-01092-0)
Supplement: Supplementary file 3 — Additional file 3:. Supplementary file 3: Figure. S1. Kaplan–Meier survival analysis of DTFUD. Figure. S2. Frequency distribution of age of onset for heroin use. [file 12881_2020_1092_MOESM3_ESM.docx]

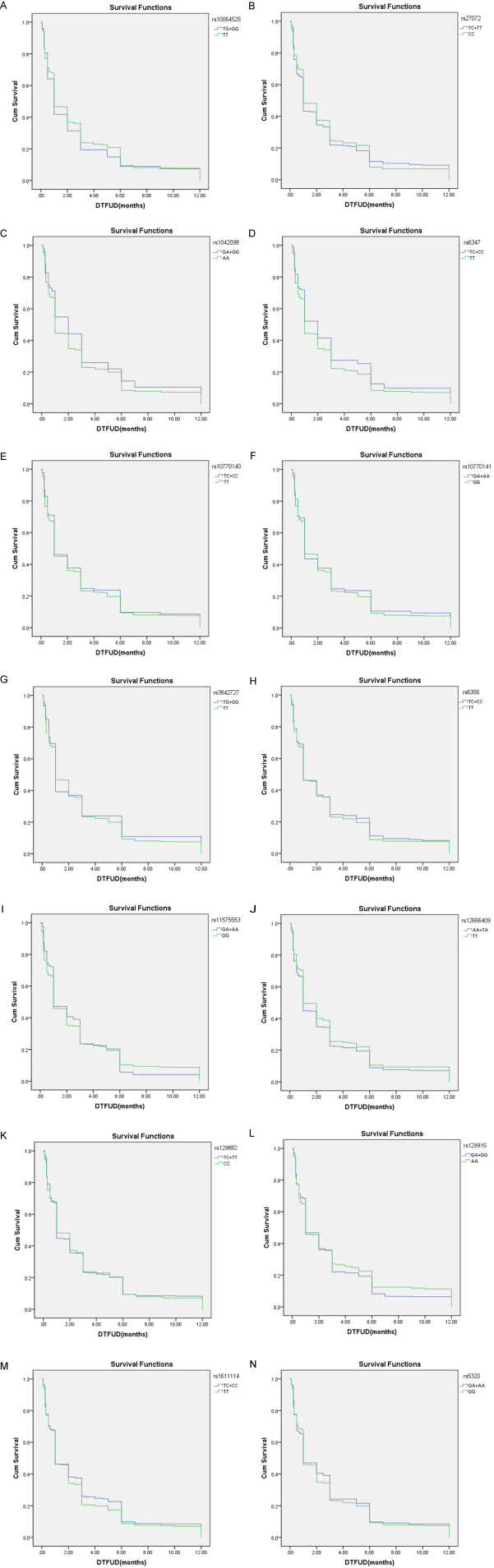


Fig. S1. Kaplan–Meier survival analysis of DTFUD. (A-N) Combined minor allele homozygote and heterozygote vs. major allele homozygote of rs10064525, rs27072, rs1042098, rs6347, rs10770140, rs10770141, rs3842727, rs6356, rs11575553, rs12666409, rs129882, rs129915, rs1611114 and rs5320, respectively.

**
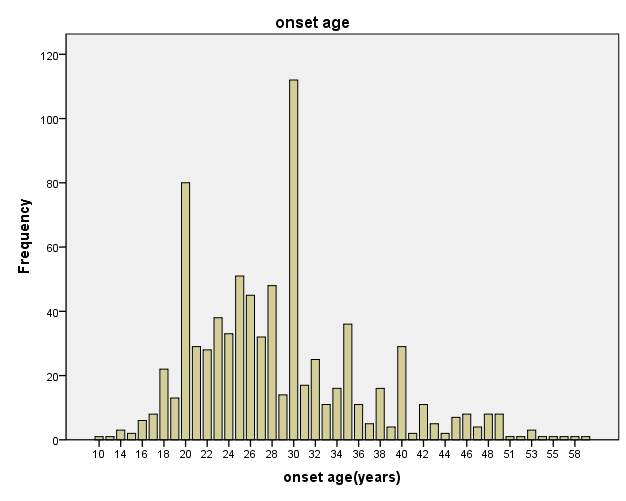
**

Fig. S2. Frequency distribution of age of onset for heroin use. The median of age of onset for heroin use was 28 years old.
