# Supplementary material for: Association studies of dopamine synthesis and metabolism genes with multiple phenotypes of heroin dependence
Source: BMC Med Genet. 2020 Jul 31;21:157. doi: 10.1186/s12881-020-01092-0 (PMC7393710; doi:10.1186/s12881-020-01092-0)
Supplement: Supplementary file 4 — Additional file 4:. Supplementary file 4: Sequence data. [file 12881_2020_1092_MOESM4_ESM.pdf]

Sample number	rs10064525	rs27072	rs1042098	rs6347	rs10770140	rs10770141	rs3842727	rs6356	rs11575553	rs12666409	rs129882	rs129915	rs1611114	rs5320
1	T/T	T/C	A/A	T/C	T/T	G/G	T/T	T/T	G/G	A/A	T/C	G/A	T/T	G/G
2	T/T	C/C	A/A	T/T	T/T	G/G	T/T	T/T	G/A	T/A	C/C	G/A	T/C	G/G
3	T/T	T/C	A/A	T/T	T/T	G/G	T/T	T/T	G/G	T/A	C/C	G/A	T/T	G/G
4	T/T	T/C	A/A	T/T	T/T	G/G	T/T	T/T	G/G	T/A	T/C	G/A	T/C	G/A
5	T/T	C/C	A/A	T/T	T/T	G/G	T/T	T/T	G/A	T/T	C/C	G/A	T/C	G/G
6	T/T	T/T	A/A	T/T	T/T	G/G	T/T	T/T	G/A	T/A	C/C	G/A	T/T	G/G
7	T/T	C/C	G/A	T/C	T/C	G/A	T/T	T/T	G/A	T/T	T/C	A/A	T/C	G/G
8	T/G	C/C	A/A	T/T	T/T	G/G	T/T	T/T	G/G	A/A	C/C	G/A	T/T	G/G
9	T/T	C/C	A/A	T/T	T/T	G/G	T/T	T/T	G/A	T/T	T/C	G/A	T/T	G/G
10	T/T	C/C		T/T	T/T	G/G	T/T	T/T	G/G	T/A	T/C	G/A	T/C	G/G
11	T/T	C/C	G/A	T/T	T/T	G/G	T/T	T/T	G/G	A/A	T/C	G/A	T/C	G/G
12	T/T	C/C	A/A	T/T	T/T	G/G	T/T	T/T	G/G	A/A	T/T	A/A	T/T	G/G
13	T/T	T/C	A/A	T/T	T/T	G/G	T/T	T/T	G/G	T/A	C/C	G/A	T/T	G/G
14	T/T	C/C	A/A	T/T	T/T	G/G	T/T	T/T	G/G	T/A	T/C	G/A	T/T	G/G
15	T/T	T/C	A/A	T/T	T/T	G/G	T/T	T/T	G/A	T/A	T/C	A/A	T/C	G/A
16	T/T	C/C	A/A	T/T	T/T	G/G	T/T	T/T	G/G	T/T	C/C	G/A	T/C	G/G
17	T/T	T/C	A/A	T/T	T/C	G/A	T/T	T/T	G/A	T/A	C/C	G/G	T/T	G/G
18	T/T	C/C	A/A	T/T	T/T	G/G	T/T	T/T	G/A	A/A	T/C	G/A	T/C	G/G
19	T/T	T/C	A/A	T/T	T/C	G/A	T/T	T/T	G/G	A/A	T/C	G/A	T/T	G/G
20	T/T	C/C	A/A	T/T	T/T	G/G	T/T	T/C	G/G	A/A	T/C	A/A	T/T	G/G
21	T/T	C/C	A/A	T/T	T/T	G/G	T/T	T/T	A/A	T/T	T/C	A/A	T/C	G/G
22	T/T	T/C	A/A	T/T	T/T	G/G	T/T	T/T	G/A	T/T	T/T	A/A	C/C	G/G
23	T/T	C/C	A/A	T/T	T/T	G/G	T/T	T/T	G/G	T/T	T/C	G/A	T/T	G/G
24	T/G	T/C	A/A	T/T	T/T	G/G	T/T	T/T	G/G	T/A	T/C	G/A	C/C	G/A
25	T/T	T/C	A/A	T/T	T/T	G/G	T/T	T/T	G/G	T/T	T/C	G/A	C/C	G/G
26	T/G	C/C	A/A	T/T	T/T	G/G	T/T	T/T	G/G	A/A	T/C	A/A	T/T	G/G
27	T/T	C/C	A/A	T/T	T/T	G/G	T/T	T/T	G/G	T/T	T/T	A/A	T/T	G/G
28	T/T	C/C	A/A	T/T	T/T	G/G	T/T	T/T	G/G	T/A	C/C	G/A	T/T	G/G
29	T/T	C/C	A/A	T/T	T/T	G/G	T/T	T/T	G/G	T/A	T/C	G/A	C/C	G/A
30	T/T	C/C	A/A	T/T	T/T	G/G	T/T	T/T	G/A	T/A	T/T	A/A	T/T	G/G
31	T/T	T/C	A/A	T/C	T/T	G/G	T/T	T/T	G/G	T/A	T/C	G/A	T/T	G/G
32	T/T	T/C	A/A	T/T	T/T	G/G	T/T	T/T	G/A	T/T	C/C	G/G	T/C	G/G
33	T/T	C/C	G/A	T/C	T/T	G/G	T/T	T/T	G/G	T/A	T/C	G/A	T/T	G/G

Sample number	rs10064525	rs27072	rs1042098	rs6347	rs10770140	rs10770141	rs3842727	rs6356	rs11575553	rs12666409	rs129882	rs129915	rs1611114	rs5320
34	T/T	C/C	A/A	T/T	T/T	G/G	T/T	T/T	G/G	T/A	T/T	A/A	T/T	G/G
35	T/T	T/C	A/A	T/T	T/C	G/A	T/G	T/C	G/G	T/A	T/T	A/A	T/C	G/G
36	T/T	C/C	A/A	T/C	T/T	G/G	T/G	T/C	G/G	T/A	T/T	A/A	T/C	G/G
37	T/T	C/C	G/A	T/C	T/T	G/G	T/T	T/T	G/A	T/A	C/C	G/G	T/C	G/A
38	T/T	C/C	A/A	T/T	T/T	G/G	T/T	T/T	G/G	T/T	T/T	A/A	T/T	G/G
39	T/T	C/C	A/A	T/T	T/T	G/G	T/T	T/T	G/G	A/A	T/C	G/A	T/C	G/A
40	T/T	T/C	A/A	T/T	T/T	G/G	T/T	T/T	G/G	A/A	T/C	G/A	T/T	G/G
41	T/T	C/C	G/A	T/C	T/T	G/G	T/T	T/T	G/G	A/A	T/C	G/A	T/T	G/G
42	T/T	T/C	G/A	T/C	T/T	G/G	T/T	T/T	G/G	A/A	T/C	G/A	T/T	G/G
43	T/T	T/C	A/A	T/T	T/T	G/G	T/T	T/T	G/G	A/A	C/C	G/A	T/C	G/G
44	T/T	C/C	A/A	T/T	T/T	G/G	T/T	T/C	G/A	T/T	C/C	A/A	T/C	G/A
45	T/T	T/C	G/A	T/T	T/C	G/A	T/T	T/C	G/G	T/A	T/C	A/A	C/C	G/A
46	T/T	C/C	A/A	T/T	T/T	G/G	T/T	T/C	G/G	T/A	T/C	G/A	C/C	G/A
47	T/T	C/C	A/A	T/T	T/T	G/G	T/T	T/T	G/G	A/A	T/C	G/A	T/C	G/G
48	T/T	C/C	G/A	T/C	C/C	A/A	G/G	C/C	G/G	T/A	C/C	G/G	T/C	G/A
49	T/T	C/C	A/A	T/T	T/T	G/G	T/T	T/T	G/G	T/T	T/C	A/A	T/T	G/G
50	T/T	C/C	A/A	T/T	T/T	G/G	T/T	T/T	G/G	T/A	T/C	G/A	T/T	G/G
51	T/T	C/C	G/A	T/T	T/T	G/G	T/G	T/T	G/G	T/A	C/C	G/A	T/C	G/A
52	T/T	T/C	G/A	T/T	T/T	G/G	T/T	T/T	G/G	T/A	T/T	A/A	T/T	G/G
53	T/T	C/C	G/G	C/C	T/C	G/A	T/T	T/T	G/G	A/A	T/T	A/A	T/T	G/G
54	T/T	C/C	A/A	T/T	T/T	G/G	T/T	T/T	G/G	T/T	C/C	A/A	T/C	G/G
55	T/T	T/C	A/A	T/T	T/C	G/G	T/T	T/C	G/G	A/A	C/C	G/G	T/T	G/G
56	T/T	C/C	A/A	T/T	T/T	G/G	T/T	T/T	G/G	A/A	T/C	G/A	T/C	G/G
57	T/T	C/C	A/A	T/T	T/T	G/G	T/T	T/C	G/G	T/A	T/T	A/A	T/C	G/G
58	T/T	T/C	G/A	T/C	T/T	G/G	T/T	T/C	G/A	T/T	T/C	G/A	T/C	G/A
59	T/T	T/C	G/A	T/C	T/T	G/G	T/T	C/C	A/A	T/T	C/C	G/A	T/T	G/G
60	T/T	T/C	G/A	T/C	T/T	G/G	T/T	T/T	G/G	T/A	C/C	G/G	T/C	G/G
61	T/T	C/C	A/A	T/T	T/T	G/G	T/T	T/T	G/A	T/A	T/T	A/A	T/T	G/G
62	T/T	C/C	A/A	T/T	T/T	G/G	T/T	T/T	G/G	T/A	T/T	A/A	T/T	G/G
63	T/T	T/T	A/A	T/T	T/T	G/G	T/T	T/C	G/G	T/T	T/C	A/A	C/C	G/G
64	T/T	T/C	A/A	T/T	T/T	G/G	T/T	T/T	G/G	T/A	T/C	G/A	T/C	G/G
65	T/T	T/C	A/A	T/T	T/T	G/G	T/T	T/C	G/G	T/T	T/C	G/A	T/C	G/G
66	T/T	C/C	A/A	T/T	T/T	G/G	T/T	T/T	G/G	T/A	C/C	G/G	C/C	A/A

Sample number	rs10064525	rs27072	rs1042098	rs6347	rs10770140	rs10770141	rs3842727	rs6356	rs11575553	rs12666409	rs129882	rs129915	rs1611114	rs5320
67	T/T	C/C	A/A	T/T	T/T	G/G	T/T	T/C	G/A	T/A	T/T	A/A	T/T	G/G
68	T/T	C/C	G/A	T/C	T/T	G/G	T/T	T/C	G/G	T/A	T/T	A/A	C/C	G/A
69	T/T	C/C	G/A	T/C	T/T	G/G	T/T	T/T	G/A	T/A	C/C	G/A	T/C	G/A
70	T/T	C/C	A/A	T/C	T/T	G/G	T/T	T/C	G/A	T/A	T/C	G/A	T/T	G/G
71	T/T	C/C	A/A	T/T	T/C	G/A	T/G	T/C	G/A	T/T	C/C	G/G	T/C	G/G
72	T/T	T/C	G/A	T/C	T/C	G/A	T/T	T/C	G/G	T/A	T/C	G/A	T/C	G/G
73	T/T	T/C	A/A	T/T	T/T	G/G	T/T	T/T	G/A	T/A	T/C	G/A	T/C	G/G
74	T/T	C/C	A/A	T/T	T/T	G/G	T/T	T/T	G/G	T/A	C/C	G/G	T/C	G/A
75	T/G	C/C	A/A	T/C	T/T	G/G	T/T	T/T	G/G	A/A	T/C	A/A	T/T	G/G
76	T/T	T/C	A/A	T/C	T/T	G/G	T/T	T/T	G/G	A/A	C/C	G/G	T/C	G/A
77	T/T	T/C	A/A	T/T	T/T	G/G	T/T	T/T	G/G	T/A	C/C	G/A	T/T	G/G
78	T/T	T/C	A/A	T/T	T/T	G/G	T/T	T/T	G/G	A/A	C/C	G/G	C/C	G/G
79	T/T	C/C	A/A	T/T	T/T	G/G	T/T	T/T	G/G	A/A	C/C	G/A	C/C	G/A
80	T/T	T/C	A/A	T/T	T/T	G/G	T/T	T/T	G/G	T/T	T/C	A/A	T/T	G/G
81	T/T	T/C	A/A	T/C	T/T	G/G	T/T	T/T	G/G	T/A	T/C	A/A	T/C	G/G
82	T/T	C/C	A/A	T/T	T/T	G/G	T/T	T/T	G/G	T/A	T/C	G/A	T/C	G/G
83	T/T	C/C	A/A	T/C	T/T	G/G	T/T	T/T	G/A	T/T	C/C	G/A	T/C	G/A
84	T/T	C/C	G/A	T/C	T/T	G/G	T/T	T/T	G/A	T/A	T/T	A/A	T/C	G/A
85	T/T	T/C	A/A	T/T	T/T	G/G	T/T	T/C	G/G	T/T	T/C	A/A	C/C	A/A
86	T/T	C/C	G/A	T/C	T/T	G/G	T/T	T/T	G/G	T/A	C/C	G/A	T/T	G/G
87	T/T	C/C	A/A	T/T	T/T	G/G	T/T	T/C	G/G	T/T	C/C	A/A	T/C	G/G
88	T/T	T/C	A/A	T/T	T/C	G/A	T/G	T/T	G/G	T/A	T/T	A/A	C/C	G/G
89	T/T	T/C	A/A	T/T	T/T	G/G	T/T	T/T	G/G	T/A	T/C	G/A	T/T	G/G
90	T/G	T/C	A/A	T/T	T/T	G/G	T/T	T/T	G/G	T/A	T/C	G/A	C/C	G/A
91	T/T	T/C	A/A	T/T	T/T	G/G	T/T	T/T	G/G	T/A	T/C	A/A	T/C	G/G
92	T/T	T/C	A/A	T/T	T/T	G/G	T/T	T/T	G/G	T/A	T/C	G/A	T/T	G/A
93	T/T	C/C	G/A	T/C	T/C	G/A	T/G	T/C	G/G	T/T	T/C	G/A	T/C	G/G
94	T/T	C/C	A/A	T/T	T/T	G/G	T/T	T/T	G/G	T/A	C/C	G/A	T/T	G/G
95	T/T	C/C	A/A	T/T	T/C	G/A	T/G	T/C	G/G	T/A	T/C	A/A	T/T	G/G
96	T/T	C/C	A/A	T/T	T/T	G/G	T/T	T/T	G/G	T/A	T/C	A/A	T/C	G/A
97	T/T	T/C	A/A	T/T	T/T	G/G	T/T	T/T	G/G	T/A	C/C	G/G	T/T	G/G
98	T/T	C/C	A/A	T/T	T/T	G/G	T/G	T/C	G/G	T/A	C/C	A/A	T/T	G/G
99	T/T	C/C	A/A	T/T	T/T	G/G	T/G	T/T	G/G	T/A	T/T	A/A	T/C	G/G

Sample number	rs10064525	rs27072	rs1042098	rs6347	rs10770140	rs10770141	rs3842727	rs6356	rs11575553	rs12666409	rs129882	rs129915	rs1611114	rs5320
100	T/G	T/C	A/A	T/T	T/C	G/A	T/G	T/C	G/A	T/A	T/T	A/A	T/C	G/A
101	T/T	C/C	A/A	T/T	T/T	G/G	T/T	T/T	G/G	T/A	C/C	G/A	T/T	G/G
102	T/T	T/T	A/A	T/T	T/T	G/G	T/T	T/C	G/G	T/A	T/C	G/A	T/T	G/G
103	T/T	C/C	A/A	T/T	T/T	G/G	T/T	T/C	G/G	A/A	T/C	G/A	T/C	G/G
104	T/T	T/C	A/A	T/T	T/T	G/G	T/T	T/T	G/G	T/T	C/C	G/A	T/C	G/G
105	T/T	C/C	G/A	T/C	T/T	G/G	T/T	T/T	G/G	A/A	T/C	A/A	T/T	G/G
106	T/T	T/C	A/A	T/C	T/T	G/G	T/T	T/T	G/G	T/A	T/C	A/A	T/T	G/G
107	T/T	T/C	A/A	T/T	T/T	G/G	T/G	T/C	A/A	T/T	T/T	A/A	C/C	G/G
108	T/T	T/C	A/A	T/T	T/T	G/G	T/T	T/T	G/G	T/A	C/C	G/G	C/C	G/A
109	T/T	T/T	A/A	T/T	T/C	G/A	T/T	T/C	G/G	A/A	C/C	G/A	T/T	G/G
110	T/T	T/T	A/A	T/T	T/T	G/G	T/T	T/T	G/G	T/A	C/C	A/A	T/C	G/A
111	T/T	C/C	G/A	T/C	T/T	G/G	T/T	T/T	G/G	T/A	T/C	G/A	T/T	G/G
112	T/T	T/C	A/A	T/T	T/T	G/G	T/T	T/T	G/G	A/A	T/T	A/A	C/C	G/G
113	T/T	T/T	A/A	T/T	T/T	G/G	T/T	T/T	G/G	T/T	T/T	A/A	T/T	G/G
114	T/T	C/C	A/A	T/T	T/T	G/G	T/T	T/T	G/G	T/T	T/C	G/A	C/C	G/G
115	T/T	C/C	A/A	T/T	T/T	G/G	T/T	T/T	G/G	T/A	T/T	A/A	T/T	G/G
116	T/G	T/C	A/A	T/T	T/T	G/G	T/T	T/T	G/G	T/A	C/C	G/G	C/C	G/A
117	T/T	T/C	A/A	T/T	T/T	G/G	T/T	T/T	G/G	T/T	C/C	G/A	C/C	G/A
118	T/T	C/C	A/A	T/T	T/T	G/G	T/T	T/T	G/G	T/A	T/C	A/A	T/C	G/G
119	T/T	T/C	A/A	T/T	T/T	G/G	T/T	T/T	G/G	T/A	C/C	G/A	T/T	G/G
120	T/T	C/C	A/A	T/T	T/T	G/G	T/T	T/T	G/G	T/A	T/C	G/A	T/T	G/G
121	T/T	T/C	A/A	T/T	T/T	G/G	T/T	T/T	G/A	A/A	C/C	G/A	T/C	G/A
122	T/T	C/C	G/A	T/C	T/T	G/G	T/T	T/T	G/G	T/T	T/C	G/A	T/C	G/G
123	T/G	T/C	A/A	T/T	T/T	G/G	T/T	T/T	G/A	T/A	C/C	A/A	C/C	A/A
124	T/G	C/C	A/A	T/T	T/T	G/G	T/T	T/T	G/A	T/T	T/T	A/A	T/T	G/G
125	T/T	T/C	A/A	T/T	T/C	G/A	T/G	T/C	G/G	T/A	T/T	A/A	T/C	G/A
126	T/T	C/C	A/A	T/T	T/T	G/G	T/T	T/T	G/G	T/A	C/C	G/G	T/T	G/G
127	T/T	T/C	G/A	T/C	T/T	G/G	T/T	T/T	G/G	A/A	C/C	G/G	C/C	G/G
128	T/G	C/C	A/A	T/C	T/T	G/G	T/T	T/T	G/A	T/A	T/C	G/A	T/C	G/G
129	T/T	C/C	A/A	T/T	T/T	G/G	T/T	C/C	G/G	T/T	C/C	G/G	C/C	G/G
130	T/T	C/C	G/A	T/C	T/T	G/G	T/T	T/T	G/G	T/T	T/C	G/A	T/C	G/G
131	T/T	T/C	A/A	T/C	T/T	G/G	T/T	T/C	G/G	A/A	C/C	G/A	C/C	A/A
132	T/T	T/C	G/A	T/C	T/T	G/G	T/T	T/T	G/A	T/T	T/T	A/A	T/C	G/G

Sample number	rs10064525	rs27072	rs1042098	rs6347	rs10770140	rs10770141	rs3842727	rs6356	rs11575553	rs12666409	rs129882	rs129915	rs1611114	rs5320
133	T/T	T/T	A/A	T/T	T/T	G/G	T/T	T/C	G/G	A/A	T/C	G/A	T/C	G/G
134	T/T	T/C	A/A	T/T	T/T	G/G	T/T	T/T	G/G	T/A	T/T	G/A	T/T	G/G
135	T/T	T/C	A/A	T/T	T/T	G/G	T/T	T/T	G/A	T/T	C/C	G/A	T/C	G/G
136	T/T	T/C	A/A	T/T	T/T	G/G	T/T	T/T	G/G	T/A	T/T	A/A	T/C	G/G
137	T/T	C/C	A/A	T/T	T/T	G/G	T/T	T/T	G/G	T/A	C/C	G/G	T/C	G/A
138	T/T	T/C	A/A	T/T	T/C	G/A	T/G	C/C	G/A	T/A	C/C	G/G	T/T	G/G
139	T/T	C/C	A/A	T/T	T/T	G/G	T/T	T/T	G/G	A/A	C/C	G/A	T/T	G/G
140	T/T	C/C	A/A	T/T	T/T	G/G	T/T	T/T	G/G	T/T	C/C	G/G	T/T	G/G
141	T/G	T/C	A/A	T/T	T/T	G/G	T/T	T/T	G/G	T/A	C/C	G/G	C/C	G/A
142	T/T	T/C	A/A	T/T	T/T	G/G	T/T	T/T	G/G	T/T	T/T	A/A	T/C	G/G
143	T/G	C/C	A/A	T/T	T/T	G/G	T/T	T/T	G/G	A/A	T/C	G/A	T/C	G/G
144	T/T	T/C	A/A	T/T	T/C	G/A	T/G	T/C	G/G	T/A	T/C	G/A	T/T	G/G
145	T/T	T/C	A/A	T/T	T/T	G/G	T/T	T/T	A/A	T/T	C/C	A/A	T/T	G/G
146	T/T	T/C	A/A	T/T	T/C	G/A	T/G	T/C	G/A	T/T	T/T	A/A	T/C	G/G
147	T/T	C/C	A/A	T/T	T/T	G/G	T/T	T/T	G/G	T/A	T/C	G/A	T/C	G/G
148	T/T	T/C	A/A	T/T	T/T	G/G	T/T	T/T	G/G	T/A	C/C	G/A	C/C	G/G
149	T/T	C/C	A/A	T/T	T/T	G/G	T/T	T/T	G/G	T/T	C/C	G/G	T/C	G/G
150	T/T	C/C	A/A	T/T	T/T	G/G	T/T	T/T	G/G	A/A	T/C	G/A	T/T	G/G
151	T/T	T/C	A/A	T/T	T/T	G/G	T/T	T/T	G/G	T/A	C/C	G/G	T/T	G/G
152	T/G	C/C	A/A	T/C	T/T	G/G	T/T	T/T	G/G	T/A	C/C	G/A	T/T	G/G
153	T/T	C/C	A/A	T/T	T/T	G/G	T/T	T/T	G/G	T/A	T/C	G/A	T/C	G/G
154	T/T	C/C	G/A	T/C	T/T	G/G	T/T	T/T	G/A	T/T	C/C	A/A	T/C	G/A
155	T/T	T/C	A/A	T/T	T/T	G/G	T/T	T/T	G/G	T/A	C/C	G/G	T/C	G/G
156	T/T	C/C	A/A	T/C	T/T	G/G	T/T	T/T	A/A	T/T	C/C	G/A	T/C	G/A
157	T/T	C/C	G/A	T/C	T/T	G/G	T/T	T/T	G/G	T/A	C/C	G/G	T/T	G/G
158	T/T	T/C	A/A	T/T	T/T	G/G	T/T	T/T	G/G	A/A	T/C	G/A	T/T	G/G
159	T/T	T/C	A/A	T/T	T/T	G/G	T/T	T/T	G/G	A/A	T/C	G/A	T/T	G/G
160	T/T	C/C	A/A	T/T	T/T	G/G	T/T	T/T	G/G	T/T	T/C	G/A	T/C	G/A
161	T/T	T/C	A/A	T/T	T/T	G/G	T/T	T/T	G/G	T/A	C/C	G/A	T/C	G/A
162	T/T	C/C	A/A	T/T	T/T	G/G	T/T	T/T	G/G	T/A	T/C	G/A	T/C	G/G
163	T/T	T/C	G/A	T/C	T/T	G/G	T/T	T/T	G/G	A/A	T/C	G/A	T/T	G/G
164	T/T	T/T	A/A	T/T	T/T	G/G	T/T	T/T	G/G	T/T	T/C	G/A	T/C	G/G
165	T/T	T/C	A/A	T/T	T/T	G/G	T/T	T/T	G/G	T/T	C/C	A/A	T/T	G/G

Sample number	rs10064525	rs27072	rs1042098	rs6347	rs10770140	rs10770141	rs3842727	rs6356	rs11575553	rs12666409	rs129882	rs129915	rs1611114	rs5320
166	T/T	T/C	A/A	T/T	T/T	G/G	T/T	T/T	G/G	T/A	T/C	A/A	T/T	G/G
167	T/T	C/C	A/A	T/C	T/T	G/G	T/T	T/T	G/G	T/T	C/C	A/A	T/T	G/G
168	T/T	C/C	A/A	T/T	T/T	G/G	T/T	T/T	G/G	T/T	T/C	A/A	T/T	G/G
169	T/T	T/C	A/A	T/T	T/T	G/G	T/T	T/T	G/G	T/T	T/T	A/A	T/T	G/G
170	T/T	T/C	A/A	T/T	C/C	A/A	T/T	T/C	G/G	T/A	T/C	G/A	T/T	G/G
171	T/T	T/C		T/T	T/T	G/G	T/T	T/T	G/G	T/T	T/C	G/A	T/T	G/G
172	T/T	T/C	A/A	T/T	T/T	G/G	T/T	T/T	G/G	T/A	C/C	A/A	T/T	G/G
173	T/T	C/C	A/A	T/C	T/T	G/G	T/G	T/T	G/G	T/A	T/C	G/A	T/C	G/A
174	T/T	C/C	G/A	T/C	T/T	G/G	T/T	T/T	G/G	A/A	C/C	A/A	T/C	G/G
175	T/T	C/C	G/A	T/C	T/T	G/G	T/T	T/T	G/G	A/A	T/C	A/A	T/C	G/G
176	T/T	C/C	A/A	T/T	T/C	G/A	T/T	T/T	G/G	T/A	T/C	G/A	T/T	G/G
177	T/T	T/C	A/A	T/T	T/T	G/G	T/T	T/T	G/G	A/A	T/C	G/A	T/T	G/G
178	T/G	C/C	A/A	T/T	T/T	G/G	T/T	T/T	G/G	T/A	T/T	A/A	T/C	G/G
179	T/T	T/C	A/A	T/T	T/T	G/G	T/T	T/T	G/A	T/A	C/C	G/A	C/C	G/A
180	T/T	T/T	A/A	T/T	T/T	G/G	T/T	T/C	G/G	A/A	T/T	A/A	T/T	G/G
181	T/T	T/C	A/A	T/T	T/T	G/G	T/T	T/T	G/G	A/A	T/C	A/A	T/C	G/G
182	T/T	C/C	A/A	T/T	T/T	G/G	T/T	T/C	G/G	T/T	C/C	A/A	T/C	G/G
183	T/T	T/C	A/A	T/T	T/C	G/A	T/G	C/C	G/G	A/A	T/C	G/A	T/T	G/G
184	T/T	C/C	A/A	T/T	T/T	G/G	T/T	T/T	G/G	T/T	C/C	G/G	T/C	G/G
185	T/T	C/C	A/A	T/T	T/T	G/G	T/T	T/T	G/G	A/A	T/C	G/A	T/T	G/G
186	T/T	C/C	A/A	T/T	T/T	G/G	T/T	T/T	G/G	T/A	C/C	G/A	T/T	G/G
187	T/G	C/C	A/A	T/C	T/T	G/G	T/T	T/T	G/G	A/A	T/C	G/A	T/T	G/G
188	T/T	C/C	A/A	T/T	T/T	G/G	T/T	T/C	G/G	A/A	T/C	G/A	T/C	G/A
189	T/G	T/C	A/A	T/T	T/T	G/G	T/T	T/T	G/G	A/A	C/C	G/G	T/T	G/G
190	T/T	C/C	A/A	T/C	T/C	G/A	T/G	T/C	G/G	T/A	T/C	A/A	T/C	G/G
191	T/G	C/C	A/A	T/T	T/T	G/G	T/T	T/T	G/A	T/A	C/C	G/A	T/C	G/A
192	T/G	C/C	A/A	T/C	T/T	G/G	T/T	T/C	G/G	A/A	T/C	G/A	T/C	G/G
193	T/T	T/C	A/A	T/T	T/T	G/G	T/T	T/T	G/A	T/T	T/C	G/A	T/C	G/A
194	T/T	C/C	G/A	T/C	T/T	G/G	T/T	T/C	G/G	T/A	C/C	G/A	T/C	G/A
195	T/T	T/C	A/A	T/T	T/T	G/G	T/T	T/T	G/A	T/T	C/C	G/G	T/C	G/A
196	T/T	C/C	A/A	T/T	T/T	G/G	T/T	T/T	G/G	T/T	T/C	G/A	T/C	G/A
197	T/T	T/C	A/A	T/T	T/T	G/G	T/T	T/T	G/G	T/A	T/C	G/A	T/T	G/G
198	T/T	C/C	A/A	T/C	T/T	G/G	T/T	T/T	G/G	T/A	T/C	G/A	T/C	G/G

Sample number	rs10064525	rs27072	rs1042098	rs6347	rs10770140	rs10770141	rs3842727	rs6356	rs11575553	rs12666409	rs129882	rs129915	rs1611114	rs5320
199	T/T	C/C	A/A	T/T	T/T	G/G	T/T	T/T	G/G	T/T	T/C	G/A	T/C	G/G
200	T/T	C/C	A/A	T/T	T/T	G/G	T/T	T/T	G/A	T/A	T/C	G/A	T/T	G/G
201	T/T	T/C	A/A	T/T	T/C	G/A	T/G	C/C	A/A	T/A	T/C	A/A	T/T	G/G
202	T/T	C/C	A/A	T/T	T/T	G/G	T/T	T/T	G/G	T/A	T/C	G/A	T/C	G/G
203	T/T	C/C	A/A	T/T	T/T	G/G	T/T	T/T	G/G	T/A	C/C	G/G	T/C	G/G
204	T/T	T/T	A/A	T/T	T/C	G/A	T/G	T/C	G/G	T/A	T/C	G/A	T/C	G/G
205	T/T	C/C	G/A	T/C	T/C	G/A	T/T	T/C	G/G	T/A	T/C	G/A	T/C	G/A
206	T/T	C/C	A/A	T/T	T/C	G/A	T/T	T/C	G/G	A/A	T/C	G/A	T/C	G/G
207	T/T	T/C	A/A	T/C	T/T	G/G	T/T	T/T	G/G	A/A	T/C	A/A	T/T	G/G
208	T/T	T/C	G/A	T/T	T/T	G/G	T/T	T/T	G/G	T/A	C/C	G/A	C/C	G/A
209	T/T	T/T	A/A	T/T	T/T	G/G	T/T	T/T	G/G	T/A	T/C	G/A	T/C	G/A
210	T/T	C/C	A/A	T/C	T/T	G/G	T/T	T/T	G/A	A/A	T/C	G/A	T/C	G/G
211	T/T	C/C	A/A	T/T	T/T	G/G	T/T	T/T	G/G	T/A	C/C	G/G	T/C	G/G
212	T/T	C/C	G/A	T/C	T/T	G/G	T/T	T/T	G/A	T/T	C/C	G/G	T/C	G/G
213	T/T	T/C	G/A	T/C	T/T	G/G	T/T	T/T	A/A	T/T	C/C	G/A	T/C	G/A
214	T/T	C/C	A/A	T/T	T/T	G/G	T/T	T/T	G/G	T/A	C/C	G/G	T/T	G/G
215	T/T	C/C	A/A	T/T	T/T	G/G	T/T	T/C	G/A	T/T	T/T	A/A	T/C	G/G
216	T/T	T/C	A/A	T/T	T/T	G/G	T/T	T/C	G/A	T/A	T/T	A/A	T/T	G/G
217	T/T	C/C	G/A	T/T	T/T	G/G	T/T	T/C	G/G	A/A	C/C	G/G	C/C	G/G
218	T/T	T/T	A/A	T/T	T/T	G/G	T/T	T/T	G/G	A/A	T/T	A/A	T/T	G/A
219	T/T	C/C	A/A	T/T	T/T	G/G	T/T	T/C	G/A	T/A	T/C	A/A	C/C	G/A
220	T/T	T/T	A/A	T/T	T/T	G/G	T/T	T/T	G/A	T/A	C/C	G/A	T/T	G/G
221	T/T	C/C	G/A	T/C	T/T	G/G	T/T	T/C	G/G	T/T	T/C	G/A	T/C	G/G
222	T/T	T/C	A/A	T/T	T/T	G/G	T/T	T/T	G/G	T/T	T/C	A/A	T/T	G/G
223	T/T	T/C	A/A	T/T	T/T	G/G	T/T	T/C	G/G	A/A	T/C	G/G	C/C	G/G
224	T/T	T/C	G/A	T/C	T/T	G/G	T/T	T/T	G/G	T/T	T/C	G/A	T/C	G/G
225	T/T	C/C	G/A	T/C	T/T	G/G	T/T	T/T	G/G	T/A	C/C	A/A	T/C	G/A
226	T/T	T/C	A/A	T/T	T/T	G/G	T/T	T/C	G/G	T/A	C/C	G/G	T/C	G/A
227	T/T	C/C	G/A	T/T	T/T	G/G	T/T	T/T	G/G	T/T	C/C	A/A	T/T	G/G
228	T/T	T/C	A/A	T/T	T/T	G/G	T/T	T/T	G/G	T/A	T/T	A/A	T/T	G/G
229	T/T	C/C	A/A	T/T	T/T	G/G	T/T	T/T	G/G	T/T	T/T	A/A	T/C	G/G
230	T/T	T/C	A/A	T/T	T/T	G/G	T/T	T/T	G/G	T/A	T/C	A/A	T/T	G/G
231	T/T	T/C	A/A	T/T	T/T	G/G	T/T	T/T	G/G	T/A	T/T	A/A	T/T	G/G

Sample number	rs10064525	rs27072	rs1042098	rs6347	rs10770140	rs10770141	rs3842727	rs6356	rs11575553	rs12666409	rs129882	rs129915	rs1611114	rs5320
232	T/T	C/C	A/A	T/T	T/T	G/G	T/T	T/T	G/G	A/A	C/C	G/A	T/C	G/G
233	T/T	C/C	A/A	T/T	T/T	G/G	T/T	T/T	G/A	T/T	T/T	A/A	C/C	G/A
234	T/T	C/C	G/A	T/C	T/T	G/G	T/T	T/T	G/G	A/A	C/C	G/A	T/T	G/G
235	T/T	C/C	A/A	T/T	T/T	G/G	T/T	T/T	G/G	A/A	T/T	A/A	T/C	G/G
236	T/T	T/C	A/A	T/T	T/T	G/G	T/T	T/T	G/G	T/A	C/C	A/A	T/T	G/G
237	T/G	T/C	A/A	T/T	T/T	G/G	T/T	T/T	G/G	T/A	T/T	A/A	T/T	G/G
238	T/G	T/C	A/A	T/T	T/T	G/G	T/T	T/C	G/A	T/T	T/C	A/A	T/T	G/G
239	T/G	T/C	G/A	T/C	T/T	G/G	T/T	T/T	G/G	T/A	C/C	G/A	C/C	A/A
240	T/T	C/C	A/A	T/T	T/C	G/A	T/G	T/C	G/G	A/A	C/C	G/G	T/T	G/G
241	T/G	T/C	A/A	T/T	T/T	G/G	T/T	T/T	G/A	T/A	C/C	G/A	T/C	G/A
242	T/T	T/C	A/A	T/T	T/C	G/G	T/T	T/C	G/G	T/T	T/C	G/A	T/C	G/G
243	T/T	C/C	A/A	T/T	T/T	G/G	T/T	T/T	G/G	T/A	T/C	G/G	T/C	G/A
244	T/T	C/C	G/A	T/C	T/T	G/G	T/T	T/T	G/G	T/A	C/C	A/A	T/C	G/A
245	T/T	T/C	G/A	T/C	T/T	G/G	T/T	T/T	G/A	T/A	T/C	G/A	T/T	G/G
246	T/T	T/C	A/A	T/T	T/T	G/G	T/T	T/T	G/A	T/T	C/C	G/A	T/C	G/A
247	T/T	C/C	A/A	T/T	T/T	G/G	T/T	T/T	G/G	T/T	C/C	G/G	T/C	G/G
248	T/T	T/C	A/A	T/T	T/T	G/G	T/T	T/C	G/G	T/A	C/C	G/G	T/T	G/G
249	T/T	C/C	A/A	T/T	T/T	G/G	T/T	T/T	G/G	T/A	T/T	A/A	T/T	G/G
250	T/T	T/C	A/A	T/C	T/T	G/G	T/T	T/T	A/A	T/T	C/C	G/G	T/T	G/G
251	T/T	T/C	A/A	T/C	T/T	G/G	T/T	T/T	G/G	T/A	C/C	G/G	T/C	G/A
252	T/T	C/C	A/A	T/C	T/T	G/G	T/T	T/T	G/G	T/A	T/C	G/A	T/T	G/G
253	T/T	C/C	A/A	T/T	T/C	G/G	T/T	T/C	G/G	T/A	T/C	G/A	T/C	G/G
254	T/T	C/C	A/A	T/T	T/T	G/G	T/T	T/C	G/G	A/A	C/C	G/A	T/C	G/A
255	T/T	T/C	A/A	T/T	T/T	G/G	T/T	T/T	G/G	A/A	T/T	A/A	T/T	G/G
256	T/T	T/C	A/A	T/T	T/T	G/G	T/T	T/T	G/G	T/T	T/T	A/A	T/C	G/G
257	T/T	C/C	A/A	T/T	T/T	G/G	T/T	T/T	G/G	T/A	T/C	G/A	T/T	G/G
258	T/T	C/C	A/A	T/T	T/C	G/A	T/G	T/C	G/A	T/T	T/C	G/A	T/C	G/G
259	T/G	T/T	A/A	T/T	T/T	G/G	T/T	T/T	G/G	A/A	T/T	A/A	T/T	G/G
260	T/T	C/C	A/A	T/C	T/T	G/G	T/T	T/T	G/G	T/T	C/C	G/A	T/C	G/G
261	T/T	T/C	A/A	T/T	T/T	G/G	T/T	T/T	G/G	T/A	T/C	A/A	T/C	G/A
262	T/T	C/C	A/A	T/T	T/T	G/G	T/T	T/C	G/G	A/A	C/C	G/G	T/C	G/G
263	T/T	C/C	A/A	T/T	T/T	G/G	T/T	T/T	G/G	T/A	C/C	G/G	T/C	G/G
264	T/T	C/C	A/A	T/T	T/T	G/G	T/T	T/T	G/G	T/A	C/C	G/G	C/C	G/G

Sample number	rs10064525	rs27072	rs1042098	rs6347	rs10770140	rs10770141	rs3842727	rs6356	rs11575553	rs12666409	rs129882	rs129915	rs1611114	rs5320
265	T/T	C/C	G/G	T/T	T/T	G/G	T/T	T/C	G/G	A/A	C/C	G/A	C/C	G/A
266	T/T	T/C	A/A	T/T	T/T	G/G	T/T	T/T	G/G	A/A	C/C	G/A	T/C	G/A
267	T/T	T/C	A/A	T/T	T/T	G/G	T/T	T/T	G/G	T/A	C/C	G/A	T/C	G/A
268	T/T	C/C	A/A	T/T	T/T	G/G	T/T	T/T	G/G	A/A	T/C	G/A	T/C	G/G
269	T/T	T/C	A/A	T/T	T/T	G/G	T/T	T/C	G/G	T/T	C/C	A/A	T/T	G/G
270	T/G	T/C	A/A	T/T	T/T	G/G	T/T	T/T	G/G	A/A	C/C	G/A	T/C	G/A
271	T/T	C/C	G/A	C/C	T/T	G/G	T/T	T/C	G/G	T/A	C/C	G/A	T/C	G/A
272	T/G	T/T	A/A	T/T	T/T	G/G	T/T	T/T	G/A	T/T	C/C	G/G	C/C	G/A
273	T/T	T/C	A/A	T/T	T/T	G/G	T/T	T/T	G/G	T/A	C/C	G/A	T/T	G/G
274	T/G	T/C	A/A	T/C	T/T	G/G	T/T	T/T	G/G	T/A	T/C	G/A	T/C	G/G
275	T/T	C/C	A/A	T/T	T/T	G/G	T/T	T/T	G/G	A/A	C/C	G/G	T/T	G/G
276	T/T	C/C	A/A	T/T	T/T	G/G	T/T	T/T	G/G	A/A	C/C	G/A	C/C	G/G
277	T/T	C/C	G/G	C/C	T/C	G/A	T/T	T/C	G/A	T/A	T/C	G/A	T/C	G/A
278	T/T	C/C	A/A	T/C	T/T	G/G	T/T	T/T	G/G	A/A	C/C	G/G	T/T	G/G
279	T/T	T/C	A/A	T/T	T/T	G/G	T/T	T/T	G/G	T/T	T/C	G/A	T/T	G/G
280	T/T	T/C	A/A	T/T	T/T	G/G	T/T	T/T	G/G	A/A	T/T	A/A	T/C	G/G
281	T/G	T/C	A/A	T/T	T/C	G/A	T/G	C/C	G/G	T/A	T/C	A/A	T/C	G/A
282	T/T	C/C	A/A	T/T	T/T	G/G	T/T	T/T	G/G	A/A	C/C	G/G	T/C	G/A
283	T/T	T/C	A/A	T/T	T/C	G/A	T/T	T/C	A/A	T/T	C/C	G/G	T/T	G/G
284	T/T	C/C	G/A	T/C	T/T	G/G	T/T	T/T	G/G	T/T	T/C	G/A	T/C	G/G
285	T/T	T/C	G/A	T/T	T/T	G/G	T/T	T/T	G/G	T/T	T/T	A/A	C/C	G/A
286	T/T	T/C	A/A	T/T	T/T	G/G	T/T	T/T	G/G	T/A	T/C	A/A	T/C	G/G
287	T/T	C/C	A/A	T/T	T/T	G/G	T/T	T/T	G/G	T/T	T/C	A/A	T/T	G/G
288	T/T	C/C	A/A	T/T	T/T	G/G	T/T	T/T	G/G	A/A	T/C	G/A	T/T	G/G
289	T/T	C/C	A/A	T/T	T/T	G/G	T/T	T/T	G/G	T/A	C/C	G/G	T/C	G/G
290	T/T	T/C	A/A	T/T	T/T	G/G	T/T	T/T	G/A	T/T	T/C	G/A	T/C	G/A
291	T/T	T/C	A/A	T/T	T/T	G/G	T/T	T/C	G/A	T/T	C/C	G/G	T/C	G/G
292	T/T	T/C	A/A	T/T	T/T	G/G	T/T	T/C	G/G	T/T	T/C	A/A	T/T	G/G
293	T/T	C/C	A/A	T/C	T/T	G/G	T/T	T/T	G/G	A/A	C/C	G/G	T/C	G/G
294	T/T	T/C	A/A	T/C	T/C	G/G	T/T	T/T	G/G	T/T	C/C	G/A	T/T	G/G
295	G/G	C/C	A/A	T/C	T/T	G/G	T/T	T/T	G/G	T/A	C/C	G/A	T/C	G/G
296	T/G	C/C	A/A	T/C	T/T	G/G	T/T	T/T	G/G	A/A	T/C	G/A	T/T	G/G
297	T/T	C/C	G/A	T/C	T/T	G/G	T/T	T/T	G/G	T/A	T/C	A/A	T/T	G/G

Sample number	rs10064525	rs27072	rs1042098	rs6347	rs10770140	rs10770141	rs3842727	rs6356	rs11575553	rs12666409	rs129882	rs129915	rs1611114	rs5320
298	T/T	T/C	A/A	T/T	T/T	G/G	T/T	T/C	G/G	A/A	C/C	G/A	T/T	G/G
299	T/T	C/C	A/A	T/T	T/T	G/G	T/T	T/T	G/G	T/A	C/C	G/A	T/T	G/G
300	T/T	T/C	A/A	T/T	T/T	G/G	T/T	T/T	G/G	T/A	C/C	G/G	T/C	G/A
301	T/T	C/C	A/A	T/T	T/T	G/G	T/T	T/T	G/G	T/A	T/C	A/A	T/C	G/G
302	T/T	C/C	A/A	T/T	T/T	G/G	T/T	T/T	G/G	A/A	T/C	A/A	T/T	G/G
303	T/G	C/C	A/A	T/C	T/T	G/G	T/G	T/C	G/G	T/A	C/C	G/A	T/C	G/A
304	T/T	C/C	A/A	T/T	T/T	G/G	T/T	T/T	A/A	T/A	T/T	A/A	T/T	G/G
305	T/T	C/C	G/A	T/C	T/T	G/G	T/T	T/T	G/G	T/A	T/C	G/A	C/C	G/A
306	T/T	C/C	G/A	T/C	T/C	G/A	T/T	C/C	G/G	T/A	T/C	G/A	T/T	G/G
307	T/T	C/C	A/A	T/T	T/C	G/A	T/T	T/C	G/A	T/T	T/C	A/A	T/T	G/G
308	T/T	C/C	A/A	T/T	T/T	G/G	T/T	T/T	G/G	T/A	C/C	A/A	T/C	G/G
309	T/T	T/C	A/A	T/T	T/T	G/G	T/T	T/T	G/G	A/A	T/C	G/A	T/T	G/G
310	T/G	T/C	A/A	T/T	T/T	G/G	T/T	T/T	G/G	T/A	T/C	G/A	T/T	G/G
311	T/T	C/C	A/A	T/T	T/C	G/A	T/G	T/C	G/G	A/A	T/C	A/A	T/T	G/G
312	T/G	C/C	A/A	T/C	T/T	G/G	T/T	T/T	G/G	T/A	T/T	A/A	T/T	G/G
313	T/T	C/C	A/A	T/T	T/T	G/G	T/T	T/T	G/A	T/A	T/C	A/A	T/C	G/A
314	T/T	C/C	A/A	T/T	T/T	G/G	T/T	T/T	G/G	A/A	C/C	A/A	C/C	A/A
315	T/T	C/C	A/A	T/T	T/T	G/G	T/T	T/T	G/G	T/T	T/C	G/A	T/C	G/G
316	T/T	C/C	A/A	T/T	T/T	G/G	T/G	C/C	G/G	A/A	T/T	A/A	T/C	G/G
317	T/T	T/C	A/A	T/T	T/T	G/G	T/T	T/C	G/G	T/A	C/C	G/A	T/T	G/G
318	T/G	C/C	A/A	T/C	T/T	G/G	T/T	T/T	G/G	T/T	T/C	A/A	T/T	G/G
319	T/T	C/C	A/A	T/T	T/T	G/G	T/T	T/T	G/G	T/T	C/C	G/G	T/C	G/G
320	T/T	C/C	A/A	T/T	T/T	G/G	T/T	T/T	G/G	T/A	C/C	G/G	T/C	G/G
321	T/T	C/C	A/A	T/T	T/T	G/G	T/T	T/C	G/G	T/T	T/T	A/A	T/T	G/G
322	T/T	T/C	A/A	T/T	T/C	G/A	T/T	C/C	G/G	T/A	C/C	G/G	T/C	G/A
323	T/T	C/C	A/A	T/T	T/T	G/G	T/T	T/T	G/G	T/A	T/C	G/A	T/T	G/G
324	T/T	C/C	A/A	T/C	T/T	G/G	T/T	T/T	G/G	A/A	C/C	G/G	T/C	G/G
325	T/T	C/C	A/A	T/T	T/T	G/G	T/T	T/T	G/G	T/T	T/C	G/A	T/C	G/G
326	T/T	C/C	G/A	T/C	T/T	G/G	T/T	T/T	G/G	A/A	T/C	G/A	T/C	G/G
327	T/T	C/C	G/A	T/C	T/T	G/G	T/T	T/T	G/G	T/A	T/C	G/A	T/T	G/G
328	T/G	C/C	A/A	T/T	T/T	G/G	T/T	T/T	G/G	T/T	T/C	G/A	T/C	G/G
329	T/T	C/C	A/A	T/T	T/T	G/G	T/T	T/T	G/G	A/A	T/C	G/A	T/T	G/G
330	T/T	C/C	A/A	T/T	T/T	G/G	T/T	T/T	G/A	T/T	T/C	A/A	T/T	G/G

Sample number	rs10064525	rs27072	rs1042098	rs6347	rs10770140	rs10770141	rs3842727	rs6356	rs11575553	rs12666409	rs129882	rs129915	rs1611114	rs5320
331	T/T	C/C	A/A	T/T	T/T	G/G	T/T	T/T	G/G	T/T	T/C	A/A	T/T	G/G
332	T/T	T/C	G/A	T/C	T/T	G/G	T/T	T/T	G/G	A/A	C/C	G/A	C/C	A/A
333	T/G	C/C	G/A	T/C	T/C	G/A	T/G	C/C	G/G	A/A	T/C	G/A	T/C	G/G
334	T/T	T/C	A/A	T/T	T/C	G/A	T/G	T/C	G/G	T/T	T/C	A/A	T/T	G/G
335	T/T	T/C	A/A	T/T	T/C	G/A	T/G	T/C	G/G	T/T	T/C	A/A	T/T	G/G
336	T/G	C/C	A/A	T/C	T/T	G/G	T/T	T/T	G/A	T/A	C/C	G/A	T/T	G/G
337	T/T	C/C	G/G	T/C	T/T	G/G	T/T	T/T	G/G	T/T	C/C	G/G	T/T	G/G
338	T/T	C/C	A/A	T/C	T/T	G/G	T/T	T/T	G/G	A/A	C/C	G/A	T/C	G/A
339	T/T	C/C	A/A	T/T	T/T	G/G	T/T	T/T	G/G	T/A	C/C	G/A	T/C	G/A
340	T/T	C/C	A/A	T/T	T/T	G/G	T/T	T/T	G/G	T/T	T/C	G/A	T/C	G/G
341	T/T	T/C	A/A	T/T	T/C	G/A	T/T	T/C	G/G	T/T	C/C	G/G	T/C	G/A
342	T/T	T/C	A/A	T/T	T/T	G/G	T/T	T/C	G/G	T/A	T/T	A/A	T/T	G/G
343	T/T	T/C	A/A	T/T	T/T	G/G	T/T	T/T	G/G	T/A	C/C	A/A	T/C	G/A
344	T/G	C/C	A/A	T/T	T/C	G/A	T/T	C/C	G/G	T/A	T/C	G/A	T/C	G/A
345	T/T	T/C	A/A	T/T	T/T	G/G	T/T	T/T	G/G	T/A	C/C	G/G	T/C	G/G
346	T/T	T/C	A/A	T/T	T/T	G/G	T/T	T/C	G/G	A/A	T/C	G/A	T/T	G/G
347	T/T	T/C	A/A	T/T	T/T	G/G	T/T	T/T	G/G	T/A	T/C	A/A	C/C	G/A
348	T/T	C/C	A/A	T/C	T/T	G/G	T/T	T/T	G/G	T/T	C/C	G/A	T/C	G/G
349	T/T	C/C	A/A	T/T	T/T	G/G	T/T	T/C	G/G	T/T	T/C	A/A	T/T	G/G
350	T/T	T/C	A/A	T/T	T/C	G/A	T/G	T/C	G/G	A/A	T/C	G/A	T/T	G/G
351	T/T	T/C	A/A	T/T	T/T	G/G	T/T	T/T	G/G	T/A	C/C	G/A	T/C	G/A
352	T/T	T/C	A/A	T/T	T/C	G/A	T/G	C/C	G/G	T/A	T/T	A/A	T/T	G/G
353	T/T	T/C	A/A	T/T	T/T	G/G	T/T	T/T	G/A	T/A	T/C	G/A	T/C	G/A
354	T/T	C/C	A/A	T/T	T/T	G/G	T/T	T/C	A/A	T/T	C/C	G/A	T/C	G/A
355	T/T	C/C	A/A	T/C	T/T	G/G	T/T	T/T	G/G	T/T	T/C	G/A	T/C	G/A
356	T/T	T/C	A/A	T/T	T/T	G/G	T/T	T/T	G/G	A/A	C/C	G/G	T/T	G/G
357	T/T	T/C	A/A	T/T	T/C	G/A	T/T	T/C	G/G	A/A	C/C	G/G	T/T	G/G
358	T/T	T/C	A/A	T/T	T/T	G/G	T/T	T/T	G/G	A/A	T/T	A/A	T/T	G/G
359	T/T	T/C	A/A	T/T	T/C	G/A	T/T	T/T	G/G	T/T	C/C	A/A	T/T	G/G
360	T/T	T/C	A/A	T/T	T/T	G/G	T/T	T/T	G/A	T/T	T/C	G/A	T/C	G/A
361	T/T	C/C	A/A	T/T	T/T	G/G	T/T	T/T	G/G	A/A	C/C	G/G	T/T	G/G
362	T/T	T/C	A/A	T/T	T/C	G/A	T/T	T/C	G/G	T/A	T/T	G/A	T/C	G/A
363	T/T	T/C	A/A	T/T	T/T	G/G	T/T	T/T	G/G	A/A	T/C	G/A	T/T	G/G

Sample number	rs10064525	rs27072	rs1042098	rs6347	rs10770140	rs10770141	rs3842727	rs6356	rs11575553	rs12666409	rs129882	rs129915	rs1611114	rs5320
364	T/T	C/C	A/A	T/T	T/T	G/G	T/T	T/C	G/A	T/T	T/C	G/A	T/C	G/A
365	T/G	T/C	A/A	T/T	T/T	G/G	T/T	T/T	G/A	T/T	T/C	G/A	T/T	G/G
366	T/T	C/C	A/A	T/C	T/T	G/G	T/T	T/T	G/G	T/A	C/C	A/A	T/C	G/G
367	T/T	T/C	A/A	T/T	T/T	G/G	T/T	T/T	G/G	T/A	T/C	G/A	T/T	G/G
368	T/T	C/C	A/A	T/T	T/C	G/A	T/G	T/C	G/A	T/T	C/C	G/A	T/C	G/A
369	T/T	C/C	A/A	T/T	T/T	G/G	T/T	T/C	G/G	T/A	C/C	G/A	T/C	G/A
370	T/T	T/T	A/A	T/T	T/T	G/G	T/T	T/T	G/G	T/A	C/C	G/A	T/C	G/A
371	T/T	C/C	A/A	T/T	T/C	G/G	T/T	T/T	G/G	A/A	C/C	G/G	T/T	G/G
372	T/T	T/T	A/A	T/T	T/T	G/G	T/T	T/C	G/G	T/A	T/C	A/A	C/C	G/A
373	T/T	C/C	A/A	T/C	T/T	G/G	T/T	T/T	G/A	T/T	T/C	G/A	T/C	G/A
374	T/T	C/C	A/A	T/T	T/T	G/G	T/T	T/T	G/G	T/A	C/C	G/A	T/C	G/A
375	T/T	C/C	A/A	T/T	T/T	G/G	T/T	T/T	G/G	A/A	C/C	G/A	T/C	G/G
376	T/G	C/C	A/A	T/C	T/T	G/G	T/T	T/T	G/G	T/T	T/C	G/A	T/C	G/G
377	T/T	C/C	A/A	T/C	T/T	G/G	T/T	T/T	G/G	T/A	T/T	A/A	T/T	G/G
378	T/T	C/C	G/A	T/C	T/T	G/G	T/T	T/T	G/G	T/A	T/T	A/A	T/C	G/G
379	T/T	T/C	G/A	T/C	T/T	G/G	T/T	T/T	G/G	T/A	T/C	G/A	T/C	G/G
380	T/T	C/C	G/A	T/C	T/T	G/G	T/T	T/T	G/G	T/T	T/C	G/A	C/C	G/G
381	T/G	T/C	A/A	T/C	T/T	G/G	T/T	T/T	G/G	A/A	C/C	G/G	T/T	G/G
382	T/T	C/C	A/A	T/T	T/T	G/G	T/T	T/T	G/G	T/A	T/C	G/A	T/C	G/G
383	T/G	C/C	A/A	T/C	T/T	G/G	T/T	T/T	G/G	A/A	C/C	G/A	T/C	G/A
384	T/T	T/C	A/A	T/T	T/T	G/G	T/T	T/T	G/G	T/A	T/C	A/A	T/T	G/G
385	T/T	C/C	G/A	T/T	T/T	G/G	T/T	T/T	G/G	A/A	C/C	G/G	T/C	G/A
386	T/T	T/C	A/A	T/T	T/T	G/G	T/T	T/T	G/G	T/A	C/C	G/G	T/T	G/G
387	T/T	C/C	A/A	T/T	T/T	G/G	T/T	T/T	G/G	A/A	C/C	G/A	T/C	G/A
388	T/G	C/C	A/A	T/T	T/T	G/G	T/T	T/T	G/A	T/A	T/C	A/A	T/C	G/A
389	T/T	C/C	A/A	T/T	T/T	G/G	T/T	T/T	G/G	A/A	T/C	A/A	T/T	G/G
390	T/T	T/C		T/T	T/T	G/G	T/T	T/C	G/G	T/A	C/C	G/A	T/T	G/G
391	T/G	T/C	A/A	T/T	T/T	G/G	T/T	T/T	G/G	T/A	T/C	G/A	T/T	G/G
392	T/T	T/C		T/T	T/T	G/G	T/T	T/C	G/G	T/T	T/C	A/A	T/C	G/G
393	T/T	T/C	A/A	T/T	T/T	G/G	T/T	T/T	G/G	A/A	C/C	G/A	T/C	G/A
394	T/T	T/C	A/A	T/T	T/T	G/G	T/T	T/T	G/G	T/A	C/C	G/A	C/C	G/G
395	T/T	T/C	G/A	T/C	T/T	G/G	T/T	T/T	A/A	T/T	C/C	G/G	T/C	G/G
396	T/T	C/C	A/A	T/T	T/T	G/G	T/T	T/T	G/G	A/A	T/C	G/A	T/T	G/G

Sample number	rs10064525	rs27072	rs1042098	rs6347	rs10770140	rs10770141	rs3842727	rs6356	rs11575553	rs12666409	rs129882	rs129915	rs1611114	rs5320
397	T/G	C/C	A/A	T/T	T/T	G/G	T/T	T/T	G/A	T/T	T/C	G/A	T/C	G/G
398	T/T	T/C	A/A	T/T	T/C	G/A	T/T	T/C	G/A	T/T	T/C	G/A	T/C	G/G
399	T/T	C/C	G/A	T/C	T/T	G/G	T/T	T/T	G/A	T/A	T/C	G/A	T/T	G/G
400	T/T	C/C	A/A	T/C	T/T	G/G	T/T	T/T	G/G	T/A	C/C	G/G	T/C	G/G
401	T/T	C/C	A/A	T/C	T/C	G/A	T/T	T/T	A/A	T/T	C/C	G/A	T/C	G/A
402	T/T	C/C	A/A	T/T	T/T	G/G	T/T	T/T	G/G	A/A	T/T	A/A	T/C	G/G
403	T/T	C/C	A/A	T/T	T/T	G/G	T/T	T/C	G/A	T/A	C/C	G/A	T/C	G/A
404	T/T	C/C	A/A	T/T	T/T	G/G	T/T	T/T	G/A	T/A	T/C	G/A	T/T	G/G
405	T/T	C/C	A/A	T/T	T/T	G/G	T/T	T/T	G/G	T/A	C/C	G/G	C/C	G/A
406	T/T	C/C	A/A	T/T	T/T	G/G	T/T	T/T	G/G	T/A	T/C	G/A	C/C	G/A
407	T/T	C/C	A/A	T/T	T/T	G/G	T/T	T/T	G/G	A/A	T/T	A/A	T/C	G/G
408	T/T	T/C	A/A	T/T	T/T	G/G	T/T	T/C	G/G	T/T	T/C	A/A	T/T	G/G
409	T/T	C/C	A/A	T/T	T/T	G/G	T/T	T/T	G/A	T/A	C/C	G/G	T/T	G/G
410	T/T	C/C	A/A	T/T	T/T	G/G	T/T	T/T	G/G	T/A	T/C	G/A	T/T	G/G
411	T/T	T/C	G/A	T/T	T/T	G/G	T/T	T/T	A/A	T/T	T/C	A/A	T/T	G/G
412	T/T	T/C	A/A	T/T	T/T	G/G	T/T	T/T	G/G	T/A	T/T	A/A	T/T	G/G
413	T/T	C/C	A/A	T/T	T/T	G/G	T/T	T/T	G/G	T/A	C/C	G/G	T/C	G/A
414	T/G	T/C	A/A	T/T	T/T	G/G	T/T	T/T	G/G	T/T	C/C	A/A	C/C	A/A
415	T/T	T/T	A/A	T/T	T/T	G/G	T/T	T/T	G/G	T/A	C/C	G/G	T/T	G/G
416	T/T	C/C	A/A	T/T	T/T	G/G	T/T	T/T	G/G	A/A	C/C	G/G	T/T	G/G
417	T/T	C/C	G/A	T/C	T/T	G/G	T/T	T/T	G/A	T/T	T/C	A/A	T/C	G/G
418	T/T	T/T	A/A	T/C	T/T	G/G	T/T	T/T	A/A	T/T	T/C	A/A	T/C	G/G
419	T/T	C/C	A/A	T/T	T/T	G/G	T/T	T/T	G/G	A/A	C/C	G/A	C/C	G/A
420	T/G	T/C	A/A	T/T	T/T	G/G	T/T	T/T	G/G	T/T	C/C	A/A	T/T	G/G
421	T/T	C/C	A/A	T/T	T/T	G/G	T/T	T/T	G/G	T/A	C/C	G/G	T/T	G/G
422	T/T	T/C	G/A	T/C	T/T	G/G	T/T	T/T	G/G	T/A	T/C	G/A	T/C	G/A
423	T/T	T/C	G/A	T/T	T/T	G/G	T/T	T/C	G/A	T/A	C/C	G/A	T/C	G/G
424	T/T	C/C	A/A	T/T	T/T	G/G	T/T	T/T	G/A	T/A	C/C	G/A	T/T	G/G
425	T/T	T/C	A/A	T/C	T/C	G/A	T/T	T/C	G/G	T/T	C/C	G/A	T/T	G/G
426	T/T	C/C	A/A	T/T	T/T	G/G	T/T	T/T	G/G	T/T	T/C	A/A	T/T	G/G
427	T/T	C/C	G/A	T/T	T/T	G/G	T/T	T/C	G/G	T/A	C/C	G/A	T/C	G/A
428	T/T	C/C	A/A	T/T	T/C	G/G	T/T	T/T	G/G	A/A	C/C	G/G	T/T	G/G
429	T/T	T/T	A/A	T/T	T/T	G/G	T/T	T/T	G/G	T/T	T/C	G/A	C/C	G/G

Sample number	rs10064525	rs27072	rs1042098	rs6347	rs10770140	rs10770141	rs3842727	rs6356	rs11575553	rs12666409	rs129882	rs129915	rs1611114	rs5320
430	T/T	T/C	A/A	T/T	T/T	G/G	T/T	T/T	G/G	T/A	C/C	G/A	T/C	G/A
431	T/G	T/C	A/A	T/C	T/T	G/G	T/T	T/C	G/G	A/A	C/C	A/A	C/C	A/A
432	T/T	C/C	A/A	T/T	T/T	G/G	T/T	T/T	G/A	T/T	T/T	A/A	T/T	G/G
433	T/T	C/C	A/A	T/T	T/T	G/G	T/T	T/T	G/G	T/T	T/T	A/A	T/C	G/G
434	T/T	T/T	A/A	T/C	T/C	G/A	T/G	T/C	G/A	T/T	C/C	G/A	T/C	G/A
435	T/T	T/C	A/A	T/T	T/T	G/G	T/T	T/C	G/G	T/A	C/C	G/G	T/C	G/A
436	T/T	C/C	A/A	T/T	T/T	G/G	T/T	T/T	G/G	A/A	T/T	A/A	T/C	G/G
437	T/T	T/C	A/A	T/T	T/T	G/G	T/T	T/C	G/G	A/A	T/C	G/A	T/T	G/G
438	T/T	T/C	A/A	T/T	T/T	G/G	T/T	T/T	G/G	A/A	T/T	A/A	T/T	G/G
439	T/T	T/T	A/A	T/T	T/T	G/G	T/T	T/C	G/G	T/A	T/C	A/A	T/T	G/G
440	T/T	C/C	G/A	T/C	T/T	G/G	T/T	T/C	G/G	T/T	T/C	G/A	C/C	G/G
441	T/T	C/C	A/A	T/T	T/T	G/G	T/T	T/T	G/G	A/A	T/T	A/A	T/C	G/G
442	T/T	C/C	A/A	T/T	T/T	G/G	T/T	T/T	G/A	T/A	C/C	G/A	C/C	G/G
443	T/T	C/C	A/A	T/T	T/T	G/G	T/T	T/T	G/G	T/A	C/C	G/G	C/C	G/A
444	T/T	C/C	A/A	T/T	T/C	G/A	T/G	T/C	G/G	A/A	C/C	G/A	T/C	G/G
445	T/T	C/C	G/A	T/C	T/C	G/A	T/G	T/C	G/G	T/A	C/C	G/G	T/C	G/A
446	T/G	C/C	A/A	T/T	T/T	G/G	T/T	T/T	G/G	A/A	T/T	A/A	T/T	G/G
447	T/T	C/C	G/A	T/T	T/T	G/G	T/T	T/T	G/G	T/A	C/C	G/G	T/T	G/G
448	T/T	C/C	A/A	T/T	T/C	G/A	T/T	T/C	G/G	T/T	T/C	G/A	T/C	G/G
449	T/T	T/C	A/A	T/T	T/C	G/A	T/G	T/T	G/A	T/T	T/C	A/A	T/C	G/G
450	T/T	C/C	A/A	T/T	T/T	G/G	T/T	T/T	G/A	T/T	T/C	G/A	T/C	G/G
451	T/T	C/C	A/A	T/T	T/T	G/G	T/T	T/T	G/G	T/T	T/C	G/A	T/C	G/G
452	T/T	C/C	A/A	T/T	T/T	G/G	T/T	T/T	G/G	T/A	C/C	G/A	T/C	G/G
453	T/T	C/C	A/A	T/T	T/T	G/G	T/T	T/T	G/G	T/A	T/C	A/A	T/T	G/G
454	T/T	C/C	G/A	T/T	T/T	G/G	T/T	T/T	G/G	T/A	C/C	G/A	T/C	G/G
455	T/T	T/C	A/A	T/T	T/T	G/G	T/T	T/T	G/G	T/A	T/T	A/A	T/T	G/G
456	T/T	T/C	A/A	T/T	T/T	G/G	T/T	T/T	G/G	T/A	T/C	A/A	T/T	G/G
457	T/T	C/C	A/A	T/T	T/T	G/G	T/T	T/T	G/A	T/A	T/C	G/A	T/C	G/G
458	T/T	T/C	A/A	T/T	T/T	G/G	T/T	T/T	G/G	T/A	T/T	A/A	T/T	G/G
459	T/T	C/C	A/A	T/T	T/T	G/G	T/T	T/T	G/G	A/A	C/C	G/A	T/C	G/A
460	T/T	C/C	A/A	T/C	T/T	G/G	T/T	T/T	A/A	T/T	T/C	G/A	C/C	G/A
461	T/T	T/C	A/A	T/C	T/T	G/G	T/T	T/T	A/A	T/T	T/C	A/A	T/T	G/G
462	T/G	T/T	A/A	T/C	T/T	G/G	T/T	T/T	G/G	T/A	T/C	G/A	T/C	G/G

Sample number	rs10064525	rs27072	rs1042098	rs6347	rs10770140	rs10770141	rs3842727	rs6356	rs11575553	rs12666409	rs129882	rs129915	rs1611114	rs5320
463	T/T	T/C	A/A	T/T	T/T	G/G	T/T	T/C	G/G	T/T	C/C	G/G	T/C	G/G
464	T/T	C/C	A/A	T/T	T/T	G/G	T/T	T/C	G/G	T/T	C/C	G/A	T/T	G/G
465	T/T	C/C	A/A	T/T	T/T	G/G	T/T	T/T	G/G	A/A	C/C	G/G	T/C	G/G
466	T/T	T/C	A/A	T/T	T/T	G/G	T/T	T/T	G/A	T/A	T/T	A/A	T/T	G/G
467	T/T	C/C	A/A	T/T	T/T	G/G	T/T	T/T	G/G	T/T	T/C	G/A	C/C	G/G
468	T/T	C/C	A/A	T/T	T/T	G/G	T/T	T/C	G/A	T/A	T/C	G/A	T/T	G/G
469	T/T	C/C	G/G	C/C	T/T	G/G	T/T	T/T	G/G	T/A	T/C	G/A	T/T	G/G
470	T/G	T/C	A/A	T/T	T/T	G/G	T/T	T/T	G/G	T/T	T/C	G/G	C/C	G/G
471	T/T	C/C	A/A	T/T	T/T	G/G	T/T	T/C	G/G	A/A	T/C	G/A	T/C	G/G
472	T/T	C/C	A/A	T/T	T/T	G/G	T/T	T/T	G/G	T/T	C/C	G/G	C/C	G/A
473	T/T	T/T	A/A	T/T	T/T	G/G	T/T	T/T	G/G	T/A	T/C	A/A	T/T	G/G
474	T/T	T/C	A/A	T/T	T/T	G/G	T/T	T/T	G/G	T/A	T/C	A/A	T/C	G/A
475	T/T	T/T	A/A	T/T	T/T	G/G	T/T	T/T	G/G	T/T	T/C	A/A	T/C	G/A
476	T/T	T/C	A/A	T/T	T/T	G/G	T/T	T/T	G/G	T/A	T/C	G/A	T/C	G/G
477	T/T	T/C	A/A	T/T	T/T	G/G	T/T	T/T	G/G	A/A	C/C	G/A	T/C	G/G
478	T/G	C/C	A/A	T/T	T/T	G/G	T/T	T/T	G/G	A/A	T/C	G/A	T/C	G/A
479	T/T	C/C	A/A	T/T	T/T	G/G	T/T	T/T	G/G	A/A	T/C	G/A	C/C	G/A
480	T/T	T/C	A/A	T/T	T/T	G/G	T/T	T/T	G/A	T/A	C/C	G/G	T/C	G/G
481	T/G	C/C	G/A	T/C	T/T	G/G	T/T	T/T	G/A	T/A	T/T	A/A	T/C	G/G
482	T/T	C/C	A/A	T/T	T/T	G/G	T/T	T/T	G/G	T/A	T/T	A/A	T/C	G/G
483	T/T	T/C	A/A	T/T	T/T	G/G	T/T	T/T	G/G	T/A	T/T	A/A	T/C	G/G
484	T/G	T/C	A/A	T/C	T/T	G/G	T/T	T/T	G/G	T/A	C/C	G/A	C/C	G/G
485	T/T	C/C	G/A	T/C	T/T	G/G	T/T	T/T	G/G	T/A	T/C	G/A	T/T	G/G
486	T/T	C/C	G/A	T/C	T/T	G/G	T/T	T/T	G/A	T/T	C/C	G/A	T/C	G/A
487	T/T	T/C	A/A	T/T	T/T	G/G	T/T	T/T	G/G	A/A	T/T	A/A	C/C	G/G
488	T/T	C/C	A/A	T/T	T/T	G/G	T/T	T/T	G/G	T/A	T/C	G/A	C/C	A/A
489	T/T	C/C	A/A	T/C	T/T	G/G	T/T	T/C	G/G	A/A	C/C	G/G	C/C	G/A
490	T/T	C/C	A/A	T/T	T/T	G/G	T/T	T/T	G/G	A/A	T/C	G/A	T/C	G/G
491	T/T	C/C	A/A	T/T	T/T	G/G	T/T	T/T	G/G	A/A	C/C	G/A	T/C	G/G
492	T/T	C/C	A/A	T/T	T/C	G/A	T/G	T/C	G/G	T/A	C/C	G/G	T/C	G/G
493	T/T	C/C	A/A	T/C	T/T	G/G	T/T	T/T	G/G	T/T	C/C	G/G	T/C	G/G
494	T/T	T/C	G/A	T/T	T/C	G/A	T/T	T/T	G/G	T/A	C/C	G/G	T/C	G/G
495	T/T	T/C	A/A	T/T	T/C	G/G	T/T	T/C	G/A	T/A	C/C	G/A	C/C	G/G

Sample number	rs10064525	rs27072	rs1042098	rs6347	rs10770140	rs10770141	rs3842727	rs6356	rs11575553	rs12666409	rs129882	rs129915	rs1611114	rs5320
496	T/T	T/T	A/A	T/T	T/T	G/G	T/T	T/T	G/G	T/A	T/C	A/A	T/C	G/G
497	T/T	C/C	G/A	T/T	T/T	G/G	T/T	T/T	G/G	T/A	T/C	G/A	T/C	G/G
498	T/G	C/C	A/A	T/T	T/T	G/G	T/T	T/T	G/G	T/T	C/C	G/G	T/T	G/G
499	T/T	C/C	A/A	T/T	T/T	G/G	T/T	T/T	G/A	T/A	C/C	G/A	T/C	G/A
500	T/T	C/C	A/A	T/T	T/C	G/A	T/T	T/C	G/G	T/A	T/C	G/A	T/C	G/A
501	T/T	C/C	A/A	T/T	T/T	G/G	T/T	T/T	G/G	T/A	C/C	G/A	T/C	G/A
502	T/T	T/C	A/A	T/T	T/T	G/G	T/T	T/T	G/G	T/A	C/C	G/A	T/C	G/A
503	T/T	T/C	A/A	T/T	T/T	G/G	T/T	T/C	G/G	A/A	C/C	G/A	T/C	G/A
504	T/T	C/C	A/A	T/T	T/T	G/G	T/T	T/T	G/A	T/A	C/C	G/A	T/T	G/G
505	T/T	C/C	G/A	T/C	T/T	G/G	T/T	T/T	G/G	T/A	C/C	G/G	T/C	G/A
506	T/T	C/C	A/A	T/T	T/T	G/G	T/T	T/T	G/G	T/A	C/C	G/A	T/T	G/G
507	T/G	T/T	A/A	T/T	T/T	G/G	T/T	T/C	G/G	T/A	C/C	G/G	T/C	G/A
508	T/T	C/C	A/A	T/T	T/T	G/G	T/T	T/T	G/G	T/A	C/C	G/G	T/C	G/G
509	T/G	T/C	A/A	T/T	T/T	G/G	T/T	T/T	G/G	T/A	T/C	G/A	C/C	G/A
510	T/T	T/T	A/A	T/C	T/T	G/G	T/T	T/T	G/G	T/A	T/C	G/A	T/T	G/G
511	T/T	C/C	G/A	T/C	T/C	G/A	T/G	T/C	G/G	A/A	C/C	G/G	T/C	G/A
512	T/T	C/C	G/G	C/C	T/C	G/A	T/G	T/C	G/G	T/T	T/T	A/A	T/T	G/G
513	T/T	T/C	A/A	T/T	T/T	G/G	T/T	T/T	G/G	A/A	C/C	G/A	T/C	G/G
514	T/T	T/C	A/A	T/T	T/T	G/G	T/T	T/T	G/G	T/A	T/C	G/A	T/T	G/G
515	T/T	T/C	A/A	T/T	T/C	G/A	T/T	T/C	G/G	T/A	T/C	A/A	T/C	G/G
516	T/T	C/C	A/A	T/T	T/T	G/G	T/T	T/T	G/G	A/A	T/C	G/A	T/T	G/G
517	T/T	C/C	G/A	T/C	T/T	G/G	T/T	T/T	G/A	T/T	C/C	G/G	T/T	G/G
518	T/T	T/C	A/A	T/T	T/T	G/G	T/T	T/C	G/G	T/A	T/C	G/A	C/C	G/A
519	T/T	T/T	G/A	T/T	T/T	G/G	T/T	T/C	G/G	T/A	T/C	G/A	T/T	G/G
520	T/T	T/C	A/A	T/T	T/C	G/A	T/G	T/C	G/G	T/A	C/C	G/G	T/C	G/G
521	T/T	T/T	A/A	T/T	T/T	G/G	T/T	T/T	G/G	A/A	T/C	G/A	T/C	G/A
522	T/T	C/C	A/A	T/T	T/T	G/G	T/T	T/C	G/G	A/A	T/C	G/A	C/C	G/A
523	T/T	C/C	A/A	T/C	T/T	G/G	T/T	T/T	A/A	T/T	T/C	G/A	C/C	G/A
524	T/T	C/C	G/A	T/T	T/T	G/G	T/T	T/C	G/G	A/A	C/C	G/G	C/C	G/G
525	T/T	T/C	A/A	T/T	T/T	G/G	T/T	T/T	G/G	A/A	T/C	G/A	T/T	G/G
526	T/T	T/C	A/A	T/T	T/T	G/G	T/T	T/T	G/G	T/A	T/T	A/A	T/C	G/G
527	T/T	C/C	A/A	T/T	T/C	G/A	T/T	C/C	G/G	T/A	C/C	G/G	T/C	G/A
528	T/T	C/C	G/A	T/C	T/T	G/G	T/T	T/C	G/G	T/T	C/C	G/A	T/T	G/G

Sample number	rs10064525	rs27072	rs1042098	rs6347	rs10770140	rs10770141	rs3842727	rs6356	rs11575553	rs12666409	rs129882	rs129915	rs1611114	rs5320
529	T/T	C/C	G/A	T/T	T/T	G/G	T/T	T/C	G/G	T/A	T/C	G/A	T/C	G/A
530	T/T	T/C	A/A	T/T	T/T	G/G	T/T	T/T	G/G	A/A	C/C	G/G	T/C	G/A
531	T/T	T/C	A/A	T/T	T/T	G/G	T/T	T/T	G/G	T/T	T/C	G/A	T/T	G/G
532	T/T	C/C	A/A	T/T	T/C	G/A	T/T	T/T	G/G	T/A	C/C	A/A	T/T	G/G
533	T/T	C/C	A/A	T/T	T/C	G/A	T/G	T/C	G/A	T/A	T/C	G/A	T/T	G/G
534	T/T	T/T	A/A	T/C	T/T	G/G	T/T	T/T	G/A	T/A	T/T	A/A	T/C	G/A
535	T/T	T/T	A/A	T/C	T/T	G/G	T/T	T/T	G/G	A/A	C/C	A/A	C/C	G/A
536	T/T	C/C	G/A	T/T	T/C	G/A	T/G	T/C	G/G	T/A	T/C	G/A	T/C	G/G
537	T/T	T/C	A/A	T/T	T/T	G/G	T/T	T/T	A/A	T/T	T/C	A/A	T/C	G/G
538	T/T	T/C	A/A	T/T	T/T	G/G	T/T	T/C	G/G	A/A	T/C	G/A	T/T	G/G
539	T/T	C/C	A/A	T/T	T/T	G/G	T/T	T/T	G/G	A/A	C/C	G/A	T/C	G/A
540	T/T	T/T	A/A	T/T	T/T	G/G	T/T	T/T	G/A	T/T	T/T	A/A	T/C	G/G
541	T/T	T/T	A/A	T/T	T/T	G/G	T/T	T/T	G/G	T/A	C/C	G/A	T/T	G/G
542	T/T	C/C	G/A	T/C	T/T	G/G	T/T	T/T	G/G	A/A	T/C	G/A	T/T	G/G
543	T/T	C/C	G/A	T/T	T/T	G/G	T/T	T/T	G/G	T/A	T/C	A/A	C/C	G/A
544	T/T	T/C	A/A	T/T	T/T	G/G	T/T	T/T	G/G	T/A	C/C	G/A	T/C	G/A
545	T/T	C/C	A/A	T/T	T/T	G/G	T/T	T/T	G/G	T/A	T/C	G/A	T/T	G/G
546	T/T	C/C	A/A	T/T	T/T	G/G	T/T	T/C	A/A	T/T	T/C	G/A	T/T	G/G
547	T/T	T/C	A/A	T/T	T/T	G/G	T/T	T/T	G/G	T/A	C/C	G/G	T/T	G/G
548	T/T	T/C	A/A	T/T	T/C	G/A	T/T	T/T	G/A	T/T	C/C	G/A	T/T	G/G
549	T/G	C/C	A/A	T/T	T/T	G/G	T/T	T/C	G/G	T/T	T/T	A/A	T/T	G/G
550	T/T	C/C	A/A	T/T	T/T	G/G	T/T	T/T	G/G	T/A	T/C	A/A	T/T	G/G
551	T/G	T/C	A/A	T/T	T/T	G/G	T/T	T/T	G/G	T/A	T/T	A/A	T/T	G/G
552	T/T	T/C	G/A	T/C	T/T	G/G	T/T	T/T	G/G	A/A	T/C	A/A	T/C	G/G
553	T/T	T/C	A/A	T/T	T/T	G/G	T/T	T/T	G/G	T/A	T/C	G/A	T/T	G/G
554	T/T	T/C	A/A	T/T	T/T	G/G	T/T	T/C	G/G	T/A	T/C	G/A	T/T	G/G
555	T/T	C/C	A/A	T/T	T/T	G/G	T/T	T/T	G/G	T/A	T/T	A/A	T/C	G/G
556	T/T	T/C	G/A	T/T	T/T	G/G	T/T	T/T	G/G	T/A	T/T	A/A	C/C	G/A
557	T/T	T/C	A/A	T/T	T/C	G/A	T/T	T/C	G/G	T/T	T/T	A/A	T/T	G/G
558	T/T	C/C	A/A	T/T	T/T	G/G	T/T	T/C	G/G	T/T	C/C	G/A	C/C	G/A
559	T/T	T/C	A/A	T/T	T/T	G/G	T/T	T/T	G/A	T/A	T/T	A/A	C/C	G/G
560	T/G	C/C	A/A	T/C	T/T	G/G	T/T	T/T	G/G	A/A	C/C	G/A	T/T	G/G
561	T/T	T/C	A/A	T/T	T/T	G/G	T/T	T/T	G/A	T/T	T/C	G/A	T/T	G/G

Sample number	rs10064525	rs27072	rs1042098	rs6347	rs10770140	rs10770141	rs3842727	rs6356	rs11575553	rs12666409	rs129882	rs129915	rs1611114	rs5320
562	T/T	T/C	A/A	T/T	T/T	G/G	T/T	T/T	G/G	A/A	T/T	A/A	T/T	G/G
563	T/T	C/C	G/A	T/C	T/T	G/G	T/T	T/T	G/G	T/A	C/C	G/A	T/C	G/A
564	T/T	T/T	A/A	T/T	T/T	G/G	T/T	T/T	G/G	T/A	T/C	G/A	T/T	G/G
565	T/T	C/C	A/A	T/T	T/T	G/G	T/T	T/T	G/G	T/T	C/C	G/A	T/C	G/A
566	T/T	T/T	A/A	T/T	T/T	G/G	T/T	T/T	G/G	A/A	T/C	G/A	T/T	G/G
567	T/T	C/C	A/A	T/T	T/T	G/G	T/T	T/T	G/A	T/A	T/C	G/A	T/T	G/G
568	T/T	T/C	A/A	T/T	T/T	G/G	T/T	T/T	G/G	T/A	T/T	A/A	T/T	G/G
569	T/T	C/C	A/A	T/T	T/T	G/G	T/T	T/T	G/G	A/A	C/C	G/G	T/T	G/G
570	T/T	T/C	A/A	T/T	T/T	G/G	T/T	T/T	G/G	A/A	C/C	A/A	T/C	G/A
571	T/G	T/C	A/A	T/T	T/T	G/G	T/T	T/T	G/G	A/A	T/C	G/A	T/T	G/G
572	T/T	C/C	A/A	T/T	T/T	G/G	T/T	T/T	G/G	T/A	T/C	A/A	T/C	G/G
573	T/T	T/C	A/A	T/T	T/T	G/G	T/T	T/T	G/A	T/T	C/C	G/A	T/T	G/G
574	T/T	C/C	A/A	T/T	T/T	G/G	T/T	T/C	G/G	T/A	T/T	A/A	T/C	G/G
575	T/T	T/T	A/A	T/T	T/T	G/G	T/T	T/T	G/G	T/T	T/C	A/A	T/C	G/A
576	T/T	C/C	A/A	T/T	T/C	G/A	T/T	T/C	G/G	T/T	T/C	G/A	T/C	G/A
577	T/G	T/C	G/A	T/C	T/T	G/G	T/T	T/C	G/G	T/A	C/C	G/G	T/C	G/A
578	T/T	T/C	A/A	T/T	T/T	G/G	T/T	T/C	G/G	T/A	T/T	A/A	T/T	G/G
579	T/T	T/T	A/A	T/T	T/T	G/G	T/T	T/T	G/G	T/A	T/C	G/A	T/T	G/G
580	T/T	C/C	A/A	T/T	T/T	G/G	T/T	T/T	G/G	T/A	T/C	G/A	T/C	G/G
581	T/T	T/C	A/A	T/T	T/T	G/G	T/T	T/C	G/A	T/T	T/C	G/A	T/T	G/G
582	T/G	C/C	A/A	T/C	T/T	G/G	T/T	T/C	G/G	T/T	T/C	G/A	T/T	G/G
583	T/T	T/C	A/A	T/C	T/T	G/G	T/T	T/T	G/A	T/A	T/C	G/A	T/T	G/G
584	T/T	T/C	A/A	T/T	T/T	G/G	T/T	T/T	G/G	A/A	T/C	G/A	T/T	G/G
585	T/T	C/C	A/A	T/T	T/C	G/A	T/T	T/C	G/G	A/A	C/C	G/G	C/C	A/A
586	T/T	C/C	A/A	T/T	T/T	G/G	T/T	T/T	G/G	A/A	T/C	G/A	T/T	G/G
587	T/T	T/C	A/A	T/T	T/T	G/G	T/T	T/T	G/G	T/A	C/C	G/A	C/C	A/A
588	T/T	C/C	A/A	T/T	T/T	G/G	T/T	T/T	G/G	T/A	T/C	G/A	T/T	G/G
589	T/T	T/C	A/A	T/T	T/T	G/G	T/G	T/C	G/G	T/A	T/C	G/A	T/T	G/G
590	T/T	C/C	A/A	T/T	T/T	G/G	T/T	T/T	G/G	T/A	T/T	A/A	T/C	G/G
591	T/T	T/C	A/A	T/C	T/T	G/G	T/T	T/T	G/G	A/A	T/C	G/A	T/T	G/G
592	T/T	T/C	A/A	T/T	T/T	G/G	T/T	T/T	G/G	T/T	T/C	G/A	T/T	G/G
593	T/G	T/C	A/A	T/T	T/T	G/G	T/T	T/C	G/G	T/A	T/C	G/A	T/T	G/G
594	T/T	C/C	A/A	T/T	T/T	G/G	T/T	T/T	G/G	T/T	C/C	A/A	T/T	G/G

Sample number	rs10064525	rs27072	rs1042098	rs6347	rs10770140	rs10770141	rs3842727	rs6356	rs11575553	rs12666409	rs129882	rs129915	rs1611114	rs5320
595	T/T	T/C	A/A	T/T	T/T	G/G	T/T	T/T	G/A	T/A	C/C	G/G	T/C	G/G
596	T/T	T/T	A/A	T/T	T/T	G/G	T/T	T/C	G/G	A/A	C/C	G/G	T/C	G/A
597	T/T	T/C	A/A	T/T	T/C	G/A	T/T	T/T	G/A	T/A	T/T	A/A	T/T	G/G
598	T/T	T/C	A/A	T/C	T/T	G/G	T/T	T/T	G/A	T/A	T/C	G/A	T/T	G/G
599	T/T	C/C	A/A	T/T	T/T	G/G	T/T	T/T	G/G	T/A	T/T	G/A	T/T	G/G
600	T/T	C/C	A/A	T/T	T/T	G/G	T/T	T/T	G/G	T/A	T/C	G/A	T/C	G/G
601	T/T	C/C	A/A	T/T	T/T	G/G	T/T	T/C	G/A	T/A	C/C	G/A	T/T	G/G
602	T/G	C/C	A/A	T/C	T/C	G/A	T/T	T/T	G/G	T/A	C/C	G/G	C/C	G/G
603	T/T	T/C	A/A	T/T	T/T	G/G	T/T	T/T	G/A	T/T	C/C	G/G	T/T	G/G
604	T/T	C/C	A/A	T/T	T/C	G/A	T/T	T/T	G/A	T/A	T/C	G/A	T/C	G/G
605	T/T	T/C	A/A	T/T	T/C	G/G	T/T	T/C	G/A	T/A	T/T	A/A	T/T	G/G
606	T/T	T/C	A/A	T/C	T/T	G/G	T/T	T/T	G/G	T/A	T/T	A/A	T/T	G/G
607	T/T	C/C	A/A	T/T	T/T	G/G	T/T	T/T	G/A	T/T	C/C	G/A	T/C	G/A
608	T/T	C/C	A/A	T/T	T/T	G/G	T/T	T/T	G/G	T/T	T/C	G/A	T/T	G/G
609	T/T	C/C	A/A	T/T	T/T	G/G	T/T	T/C	G/G	T/A	C/C	A/A	T/C	G/G
610	T/G	T/C	A/A	T/T	T/T	G/G	T/T	T/T	G/G	T/A	T/C	G/A	T/T	G/G
611	T/T	C/C	A/A	T/T	T/T	G/G	T/T	T/T	G/G	T/T	C/C	G/G	C/C	A/A
612	T/T	C/C	G/A	T/C	T/T	G/G	T/T	T/T	G/A	T/A	T/C	G/A	T/T	G/G
613	T/T	C/C	A/A	T/T	T/T	G/G	T/G	T/C	G/A	T/A	C/C	G/G	C/C	G/A
614	T/G	T/T	A/A	T/T	T/C	G/A	T/G	C/C	G/G	A/A	C/C	G/A	T/C	G/A
615	T/T	C/C	A/A	T/T	T/T	G/G	T/T	T/T	G/G	T/A	C/C	G/A	T/T	G/G
616	T/T	T/C	A/A	T/T	T/T	G/G	T/T	T/T	G/G	A/A	T/C	A/A	C/C	A/A
617	T/T	C/C	A/A	T/T	T/T	G/G	T/T	T/T	G/A	T/T	T/C	A/A	T/T	G/G
618	T/T	C/C	A/A	T/T	T/T	G/G	T/T	T/C	G/A	T/T	C/C	G/G	C/C	A/A
619	T/T	C/C	A/A	T/T	T/T	G/G	T/T	T/T	G/A	T/T	C/C	G/G	T/C	G/G
620	T/T	C/C	A/A	T/T	T/C	G/A	T/G	T/C	G/G	T/A	T/C	G/A	C/C	G/A
621	T/T	C/C	A/A	T/C	T/T	G/G	T/T	T/C	G/A	T/A	C/C	G/G	T/T	G/G
622	T/T	C/C	G/A	T/C	T/T	G/G	T/T	T/T	G/G	A/A	T/T	A/A	T/C	G/G
623	T/T	C/C	A/A	T/T	T/T	G/G	T/T	T/T	G/G	T/T	T/C	A/A	T/T	G/G
624	T/T	C/C	A/A	T/T	T/T	G/G	T/T	T/C	G/G	T/A	C/C	G/A	T/C	G/G
625	T/T	T/C	A/A	T/T	T/T	G/G	T/T	T/T	G/G	T/A	T/T	A/A	T/C	G/G
626	T/T	T/C	A/A	T/T	T/T	G/G	T/T	T/C	G/G	T/T	T/C	A/A	T/T	G/G
627	T/T	C/C	A/A	T/T	T/T	G/G	T/T	T/C	G/G	T/T	C/C	G/G	T/T	G/G

Sample number	rs10064525	rs27072	rs1042098	rs6347	rs10770140	rs10770141	rs3842727	rs6356	rs11575553	rs12666409	rs129882	rs129915	rs1611114	rs5320
628	T/T	C/C	A/A	T/T	T/T	G/G	T/T	T/T	G/G	T/A	T/C	G/A	T/C	G/G
629	T/G	C/C	G/A	T/T	T/T	G/G	T/T	T/T	G/G	T/T	T/C	G/A	T/C	G/A
630	T/T	T/C	A/A	T/T	T/C	G/A	T/G	T/C	G/G	T/A	C/C	G/G	T/C	G/A
631	T/T	C/C	G/A	T/C	T/T	G/G	T/T	T/T	G/A	T/A	C/C	G/G	C/C	G/G
632	T/T	T/T	A/A	T/C	T/T	G/G	T/T	T/T	G/G	A/A	C/C	A/A	C/C	G/A
633	T/T	C/C	G/A	T/T	T/T	G/G	T/T	T/T	G/G	T/A	T/T	A/A	T/C	G/A
634	T/T	C/C	A/A	T/T	T/T	G/G	T/T	T/T	G/A	T/A	T/C	G/A	T/T	G/G
635	T/T	T/C	A/A	T/T	T/T	G/G	T/T	T/C	G/A	T/A	T/C	G/A	T/T	G/G
636	T/T	C/C	A/A	T/T	T/T	G/G	T/T	T/T	G/G	T/A	T/C	A/A	T/C	G/G
637	T/T	T/C	A/A	T/T	T/T	G/G	T/T	T/T	G/A	T/A	T/C	G/A	T/T	G/G
638	T/G	T/C	A/A	T/T	T/T	G/G	T/T	T/C	G/G	A/A	C/C	G/G	T/C	G/G
639	T/T	C/C	A/A	T/T	T/T	G/G	T/T	T/T	G/G	A/A	C/C	G/A	T/T	G/G
640	T/T	C/C	A/A	T/T	T/T	G/G	T/T	T/T	G/A	T/A	C/C	G/G	T/T	G/G
641	T/T	C/C	G/A	T/C	T/C	G/A	T/T	T/C	G/G	A/A	C/C	G/G	T/T	G/G
642	T/T	C/C	A/A	T/T	T/T	G/G	T/T	T/T	G/G	T/T	T/T	A/A	T/C	G/G
643	T/T	T/C	A/A	T/T	T/T	G/G	T/T	T/T	G/G	T/A	T/T	A/A	T/C	G/G
644	T/T	T/C	G/A	T/T	T/T	G/G	T/T	T/T	G/G	T/A	T/T	A/A	T/T	G/G
645	T/T	C/C	A/A	T/T	T/T	G/G	T/T	T/T	G/G	T/T	T/C	G/A	T/C	G/G
646	T/T	T/C	G/A	T/C	T/T	G/G	T/T	T/T	G/G	T/A	T/C	G/A	T/C	G/A
647	T/T	C/C	G/A	T/T	T/T	G/G	T/T	T/T	G/G	A/A	C/C	G/G	T/T	G/G
648	T/T	T/C	A/A	T/T	T/T	G/G	T/T	T/T	G/G	T/A	C/C	G/A	T/T	G/G
649	T/T	T/C	A/A	T/T	T/T	G/G	T/T	C/C	G/G	A/A	T/C	G/A	T/C	G/G
650	T/T	C/C	A/A	T/T	T/C	G/A	T/G	T/C	G/G	T/T	C/C	G/A	C/C	G/A
651	T/T	T/C	G/A	C/C	T/T	G/G	T/T	T/T	G/G	T/T	T/C	G/A	T/T	G/G
652	T/T	T/C	G/A	T/C	T/C	G/A	T/G	C/C	G/G	T/A	T/C	G/A	T/C	G/G
653	T/T	T/C	A/A	T/T	T/T	G/G	T/T	T/C	G/G	A/A	C/C	G/G	T/C	G/G
654	T/T	C/C	G/A	T/T	T/T	G/G	T/T	T/T	G/G	T/A	T/C	A/A	T/T	G/G
655	T/T	C/C	A/A	T/T	T/T	G/G	T/T	T/T	G/G	A/A	T/T	A/A	C/C	G/A
656	T/T	C/C	A/A	T/T	T/T	G/G	T/T	T/T	G/G	T/A	C/C	G/A	T/C	G/A
657	T/T	C/C	G/A	T/C	T/T	G/G	T/T	T/T	G/G	T/T	T/T	A/A	C/C	G/G
658	T/T	T/T	A/A	T/C	T/T	G/G	T/T	T/T	G/G	A/A	T/C	G/A	T/T	G/G
659	T/T	C/C	G/A	T/C	T/T	G/G	T/T	T/T	G/G	T/A	T/C	G/A	T/C	G/G
660	T/T	C/C	A/A	T/T	T/C	G/A	T/G	T/C	G/G	A/A	C/C	G/G	T/T	G/G

Sample number	rs10064525	rs27072	rs1042098	rs6347	rs10770140	rs10770141	rs3842727	rs6356	rs11575553	rs12666409	rs129882	rs129915	rs1611114	rs5320
661	T/G	C/C	A/A	T/T	T/T	G/G	T/T	T/T	G/A	T/T	T/C	A/A	T/C	G/A
662	T/T	C/C	G/A	T/T	T/T	G/G	T/T	T/T	G/G	T/T	C/C	A/A	T/T	G/G
663	T/T	T/C	A/A	T/T	T/C	G/A	T/T	T/T	G/A	T/A	C/C	G/G	T/T	G/G
664	T/T	C/C	A/A	T/C	T/T	G/G	T/T	T/T	G/G	T/A	T/T	A/A	T/C	G/G
665	T/T	C/C	A/A	T/T	T/C	G/A	T/T	T/C	G/G	A/A	C/C	G/A	T/T	G/G
666	T/T	C/C	A/A	T/T	T/T	G/G	T/T	T/C	G/G	T/A	C/C	G/A	T/C	G/G
667	T/T	C/C	A/A	T/T	T/T	G/G	T/T	T/T	G/A	T/A	C/C	G/A	T/C	G/G
668	T/G	T/C	A/A	T/T	T/T	G/G	T/T	T/T	G/G	T/T	C/C	G/G	T/T	G/G
669	T/T	T/C	A/A	T/T	T/T	G/G	T/T	T/T	G/A	T/T	T/C	G/A	T/T	G/G
670	T/T	T/C	A/A	T/T	T/T	G/G	T/T	T/C	G/G	T/A	C/C	G/G	T/T	G/G
671	T/G	C/C	A/A	T/C	T/T	G/G	T/T	T/C	G/A	T/T	T/C	G/A	T/T	G/G
672	T/T	C/C	A/A	T/T	T/T	G/G	T/T	T/T	G/A	T/A	T/C	G/A	T/C	G/G
673	T/T	C/C	A/A	T/T	T/T	G/G	T/T	T/T	G/G	T/A	C/C	G/G	T/C	G/G
674	T/T	C/C	A/A	T/T	T/T	G/G	T/T	T/T	G/G	T/T	C/C	G/A	T/C	G/A
675	T/T	T/C	A/A	T/T	T/C	G/A	T/G	T/C	G/G	T/T	T/C	G/A	T/C	G/A
676	T/T	C/C	A/A	T/C	T/T	G/G	T/T	T/T	G/A	A/A	C/C	G/G	T/T	G/G
677	T/T	C/C	A/A	T/T	T/T	G/G	T/T	T/T	G/G	T/T	T/C	A/A	T/C	G/G
678	T/T	C/C	A/A	T/T	T/T	G/G	T/T	T/T	G/G	T/A	C/C	G/A	T/C	G/A
679	T/T	T/C	A/A	T/T	T/C	G/A	T/T	T/C	G/G	T/A	T/C	A/A	C/C	G/A
680	T/T	C/C	G/A	T/C	T/T	G/G	T/T	T/T	G/G	A/A	C/C	G/G	T/C	G/G
681	T/T	C/C	A/A	T/T	T/T	G/G	T/T	T/T	G/G	A/A	C/C	G/A	T/T	G/G
682	T/T	C/C	G/A	T/C	T/T	G/G	T/T	T/C	G/G	T/A	C/C	G/G	T/T	G/G
683	T/T	T/C	A/A	T/T	T/T	G/G	T/T	T/T	G/G	T/T	T/C	G/A	T/T	G/G
684	T/T	C/C	A/A	T/T	T/C	G/A	T/T	T/C	G/G	A/A	T/C	G/A	T/C	G/G
685	T/T	C/C	G/A	T/C	T/T	G/G	T/T	T/T	G/A	T/A	T/C	G/A	T/C	G/G
686	T/G	T/C	A/A	T/T	T/T	G/G	T/T	T/T	G/G	T/T	C/C	G/G	T/C	G/G
687	T/T	C/C	A/A	T/T	T/T	G/G	T/T	T/T	G/A	T/A	C/C	G/A	T/C	G/G
688	T/T	T/C	A/A	T/T	T/T	G/G	T/T	T/T	G/G	T/A	C/C	G/G	T/T	G/G
689	T/G	T/C	A/A	T/C	T/T	G/G	T/T	T/T	G/A	T/T	C/C	G/A	C/C	G/A
690	T/T	C/C	G/A	C/C	T/T	G/G	T/T	T/T	G/G	T/A	T/C	G/A	T/C	G/G
691	T/T	C/C	A/A	T/T	T/T	G/G	T/T	T/T	G/G	T/T	T/C	A/A	T/T	G/G
692	T/T	T/C	A/A	T/T	T/T	G/G	T/T	T/T	G/G	A/A	T/C	G/A	T/T	G/G
693	T/T	C/C	A/A	T/T	T/T	G/G	T/T	T/T	G/G	T/A	C/C	A/A	T/T	G/G

Sample number	rs10064525	rs27072	rs1042098	rs6347	rs10770140	rs10770141	rs3842727	rs6356	rs11575553	rs12666409	rs129882	rs129915	rs1611114	rs5320
694	T/T	C/C	A/A	T/T	T/T	G/G	T/T	T/T	A/A	T/T	C/C	G/G	T/T	G/G
695	T/T	C/C	A/A	T/T	T/T	G/G	T/T	T/T	G/G	T/T	T/C	G/A	T/C	G/G
696	T/T	C/C	A/A	T/T	T/T	G/G	T/T	T/C	G/A	T/A	C/C	G/G	T/C	G/G
697	T/T	C/C	A/A	T/T	T/T	G/G	T/T	T/T	G/G	T/T	C/C	G/G	T/T	G/G
698	T/T	T/T	A/A	T/T	T/C	G/A	T/T	T/C	G/G	T/A	T/C	A/A	T/C	G/A
699	T/T	T/C	A/A	T/T	T/C	G/G	T/T	T/T	G/A	T/A	C/C	G/G	T/T	G/G
700	T/G	C/C	A/A	T/T	T/C	G/A	T/T	C/C	G/G	A/A	T/C	A/A	T/T	G/G
701	T/T	T/T	A/A	T/T	T/C	G/A	T/G	T/C	G/A	T/A	C/C	G/G	T/C	G/G
702	T/T	C/C	A/A	T/T	T/C	G/A	T/G	T/C	G/G	T/A	T/C	A/A	T/C	G/G
703	T/T	T/C	A/A	T/C	T/T	G/G	T/T	T/T	G/G	T/A	C/C	G/A	T/T	G/G
704	T/T	C/C	A/A	T/T	T/T	G/G	T/T	T/T	G/G	T/A	T/T	A/A	T/T	G/G
705	T/T	C/C	A/A	T/T	T/T	G/G	T/T	T/T	G/A	T/A	C/C	G/A	T/C	G/A
706	T/T	C/C	A/A	T/T	T/T	G/G	T/T	T/T	G/A	T/T	C/C	G/G	T/T	G/G
707	T/G	T/C	A/A	T/T	T/T	G/G	T/T	T/T	G/G	T/T	T/C	A/A	T/C	G/G
708	T/T	C/C	A/A	T/T	T/C	G/A	T/T	T/C	G/G	A/A	T/C	G/A	C/C	G/A
709	T/T	C/C	G/A	T/C	T/T	G/G	T/G	T/C	G/G	T/A	T/C	A/A	T/T	G/G
710	T/G	T/C	A/A	T/T	T/T	G/G	T/T	T/T	G/A	T/T	T/C	G/A	T/T	G/G
711	T/G	C/C	A/A	T/C	T/T	G/G	T/T	T/T	G/G	T/A	T/C	A/A	T/C	G/A
712	T/T	C/C	A/A	T/C	T/C	G/A	T/G	T/C	G/G	T/A	T/C	G/A	T/T	G/G
713	T/T	C/C	A/A	T/T	T/T	G/G	T/T	T/T	G/A	T/A	C/C	G/A	T/C	G/G
714	T/T	C/C	A/A	T/T	T/C	G/A	T/T	T/C	G/A	T/T	T/C	A/A	T/T	G/G
715	T/T	C/C	G/A	T/C	T/C	G/A	T/T	T/T	G/G	T/A	T/C	A/A	T/T	G/G
716	T/T	C/C	G/A	T/C	T/C	G/A	T/T	T/C	G/G	T/A	C/C	G/G	T/C	G/G
717	T/T	C/C	A/A	T/T	T/T	G/G	T/T	T/T	G/G	T/T	T/C	G/A	T/T	G/G
718	T/T	C/C	A/A	T/T	T/T	G/G	T/T	T/C	A/A	T/T	T/T	A/A	T/T	G/G
719	T/T	T/C	A/A	T/T	T/C	G/A	T/G	T/C	G/A	T/T	T/T	A/A	T/C	G/G
720	T/T	C/C	G/A	T/C	T/T	G/G	T/T	T/T	G/G	T/A	C/C	G/A	T/T	G/G
721	T/T	C/C	A/A	T/T	T/T	G/G	T/T	T/C	G/G	A/A	C/C	G/A	T/T	G/G
722	T/G	T/C	A/A	T/C	T/T	G/G	T/T	T/C	G/G	T/A	C/C	G/G	T/T	G/G
723	T/T	T/C	A/A	T/T	T/T	G/G	T/T	T/T	G/G	T/T	T/C	G/A	T/T	G/G
724	T/T	C/C	A/A	T/T	T/T	G/G	T/T	T/C	G/G	A/A	T/C	G/A	T/C	G/G
725	T/T	T/C	A/A	T/T	T/T	G/G	T/T	T/C	G/G	T/A	C/C	G/G	T/C	G/G
726	T/T	C/C	A/A	T/T	T/T	G/G	T/T	T/T	G/G	T/A	C/C	G/G	C/C	G/A

Sample number	rs10064525	rs27072	rs1042098	rs6347	rs10770140	rs10770141	rs3842727	rs6356	rs11575553	rs12666409	rs129882	rs129915	rs1611114	rs5320
727	T/T	C/C	A/A	T/T	T/C	G/A	T/G	T/C	G/A	T/A	T/C	G/A	T/C	G/G
728	T/T	C/C	A/A	T/T	T/C	G/A	T/T	C/C	G/G	T/T	T/C	G/A	T/T	G/G
729	T/T	C/C	G/A	T/T	T/T	G/G	T/T	T/T	G/G	T/A	C/C	G/G	T/C	G/G
730	T/T	C/C	A/A	T/T	T/T	G/G	T/T	T/T	G/A	T/T	T/C	G/A	T/C	G/A
731	G/G	T/T	A/A	T/T	T/T	G/G	T/T	T/T	G/G	T/A	C/C	A/A	T/T	G/G
732	T/T	T/T	A/A	T/T	T/T	G/G	T/T	T/T	G/G	T/T	T/C	G/A	T/T	G/G
733	T/T	T/C	A/A	T/T	T/T	G/G	T/T	T/T	G/A	T/A	C/C	A/A	T/T	G/G
734	T/T	C/C	A/A	T/T	T/T	G/G	T/T	T/T	G/G	T/A	C/C	G/G	T/T	G/G
735	T/T	T/C	G/A	T/T	T/T	G/G	T/T	T/T	G/A	T/A	T/C	G/A	T/T	G/G
736	T/T	C/C	G/A	T/C	T/T	G/G	T/T	T/T	G/G	T/T	T/C	G/A	T/T	G/G
737	T/T	T/C	G/A	T/C	T/T	G/G	T/T	T/T	G/G	A/A	T/C	A/A	T/C	G/G
738	T/T	T/C	A/A	T/T	T/C	G/A	T/T	T/C	G/G	T/A	C/C	G/G	T/C	G/A
739	T/T	T/T	A/A	T/T	T/T	G/G	T/T	T/T	G/G	T/A	C/C	G/A	T/T	G/G
740	T/T	T/C	A/A	T/T	T/T	G/G	T/T	T/T	G/G	A/A	T/C	G/A	T/C	G/G
741	T/T	C/C	A/A	T/T	T/T	G/G	T/T	T/T	G/G	T/A	C/C	G/G	T/T	G/G
742	T/T	C/C	A/A	T/T	T/T	G/G	T/T	T/C	G/G	T/A	C/C	G/A	T/C	G/A
743	T/T	T/C	A/A	T/T	T/T	G/G	T/T	T/T	G/G	T/A	T/C	G/A	C/C	A/A
744	T/T	T/C	A/A	T/T	T/T	G/G	T/T	T/C	G/G	T/A	C/C	G/G	T/T	G/G
745	T/T	C/C	A/A	T/T	T/T	G/G	T/T	T/T	G/G	A/A	T/C	G/A	T/C	G/G
746	T/T	C/C	G/A	T/T	T/T	G/G	T/T	T/T	G/G	A/A	T/C	G/A	T/C	G/A
747	T/T	T/T	A/A	T/T	T/T	G/G	T/T	T/T	G/G	T/T	T/C	G/A	T/T	G/G
748	T/T	C/C	A/A	T/T	T/T	G/G	T/T	T/T	G/G	A/A	C/C	G/G	T/C	G/G
749	T/G	C/C	A/A	T/C	T/T	G/G	T/T	T/C	G/G	T/A	C/C	G/A	C/C	G/A
750	T/T	T/C	A/A	T/T	T/T	G/G	T/T	T/T	G/G	A/A	C/C	G/A	T/C	G/G
751	T/G	T/C	A/A	T/T	T/T	G/G	T/T	T/T	G/A	T/A	C/C	G/A	T/T	G/G
752	T/G	C/C	A/A	T/C	T/T	G/G	T/T	T/T	G/G	T/T	T/C	G/A	T/C	G/A
753	T/T	C/C	A/A	T/T	T/C	G/A	T/T	T/C	G/G	T/A	T/C	G/A	C/C	G/A
754	T/T	C/C	A/A	T/T	T/T	G/G	T/T	T/T	G/G	A/A	T/C	G/A	T/C	G/G
755	T/T	C/C	A/A	T/T	T/T	G/G	T/T	C/C	G/A	T/T	T/C	G/A	T/T	G/G
756	T/T	T/C	A/A	T/T	T/T	G/G	T/T	T/T	G/A	T/A	C/C	G/G	T/T	G/G
757	T/T	C/C	A/A	T/T	T/T	G/G	T/T	T/T	G/G	T/A	C/C	G/G	T/C	G/G
758	T/T	C/C	A/A	T/T	T/T	G/G	T/T	T/T	G/G	A/A	T/C	G/A	C/C	G/A
759	T/T	C/C	A/A	T/T	T/C	G/A	T/G	T/C	G/G	T/A	C/C	G/G	T/T	G/G

Sample number	rs10064525	rs27072	rs1042098	rs6347	rs10770140	rs10770141	rs3842727	rs6356	rs11575553	rs12666409	rs129882	rs129915	rs1611114	rs5320
760	T/T	C/C	A/A	T/T	T/C	G/A	T/G	T/T	G/G	T/A	C/C	G/G	T/T	G/G
761	T/T	C/C	A/A	T/C	T/T	G/G	T/T	T/T	G/G	A/A	T/C	G/A	T/T	G/G
762	T/T	C/C	A/A	T/T	T/T	G/G	T/T	T/T	G/G	T/T	T/C	G/A	C/C	G/A
763	T/T	T/C	A/A	T/C	T/T	G/G	T/T	T/T	G/A	T/T	T/C	G/A	T/T	G/G
764	T/G	C/C	G/A	C/C	T/T	G/G	T/T	T/T	G/G	T/A	C/C	G/A	C/C	G/A
765	T/T	C/C	G/A	T/C	T/T	G/G	T/T	T/T	G/G	T/T	T/C	G/A	T/T	G/G
766	T/T	C/C	G/A	T/T	T/T	G/G	T/T	C/C	G/G	T/A	C/C	G/A	T/C	G/A
767	T/T	T/T	A/A	T/T	T/T	G/G	T/T	T/C	G/G	T/A	T/T	A/A	T/T	G/G
768	T/G	C/C	A/A	T/T	T/T	G/G	T/T	T/T	G/G	A/A	T/C	A/A	T/C	G/G
769	T/T	C/C	A/A	T/T	T/T	G/G	T/T	T/T	G/G	T/A	C/C	A/A	C/C	G/G
770	T/T	C/C	G/A	T/C	T/T	G/G	T/T	T/T	G/G	T/T	T/C	A/A	T/C	G/A
771	T/T	C/C	A/A	T/T	T/T	G/G	T/T	T/T	G/G	T/A	C/C	G/G	T/C	G/A
772	T/T	T/C	A/A	T/T	T/C	G/A	T/T	T/C	G/G	T/A	T/C	G/A	T/T	G/G
773	T/T	C/C		T/C	T/T	G/G	T/T	T/T	G/G	T/A	T/T	A/A	T/T	G/G
774	T/T	T/C	A/A	T/T	T/T	G/G	T/T	T/T	G/G	A/A	C/C	G/A	T/C	G/G
775	T/T	C/C	G/A	T/T	T/T	G/G	T/T	T/T	G/G	T/T	T/C	G/A	T/T	G/G
776	T/T	C/C	A/A	T/T	T/T	G/G	T/T	T/T	G/G	T/T	C/C	G/G	T/T	G/G
777	T/T	T/C	A/A	T/T	T/T	G/G	T/T	T/C	G/G	A/A	T/C	G/A	T/C	G/G
778	T/T	T/C	G/A	T/C	T/T	G/G	T/T	T/T	G/G	T/A	T/C	G/A	T/C	G/G
779	T/T	T/C	A/A	T/T	T/T	G/G	T/T	T/T	G/A	T/A	T/C	G/A	T/C	G/A
780	T/T	C/C	A/A	T/T	T/T	G/G	T/T	T/T	G/A	T/T	T/C	G/A	T/C	G/G
781	T/T	C/C	G/A	T/C	T/T	G/G	T/T	T/C	G/G	T/T	T/C	G/A	T/C	G/A
782	T/T	T/C	A/A	T/T	T/T	G/G	T/T	T/T	G/G	T/A	T/C	G/A	T/C	G/G
783	T/T	T/C	A/A	T/C	T/T	G/G	T/T	T/T	G/G	T/A	C/C	G/A	T/C	G/G
784	T/T	C/C	A/A	T/T	T/T	G/G	T/T	T/T	G/G	T/T	T/C	G/A	T/T	G/G
785	T/G	C/C	A/A	T/C	T/T	G/G	T/T	T/T	G/A	T/A	C/C	G/A	T/C	G/A
786	T/T	C/C	G/A	T/T	T/T	G/G	T/T	T/T	G/G	A/A	C/C	G/A	T/C	G/A
787	T/T	T/T	A/A	T/T	T/T	G/G	T/T	T/T	G/G	T/A	T/C	G/A	T/T	G/G
788	T/T	C/C	A/A	T/T	T/T	G/G	T/T	T/T	G/G	T/A	T/T	A/A	T/T	G/G
789	T/T	C/C	A/A	T/T	T/T	G/G	T/T	T/C	G/A	T/A	C/C	G/A	T/C	G/G
790	T/G	T/C	A/A	T/T	T/T	G/G	T/T	T/T	G/G	A/A	C/C	A/A	T/T	G/G
791	T/T	T/C	A/A	T/T	T/C	G/A	T/T	T/C	G/G	T/A	T/C	A/A	T/C	G/G
792	T/T	C/C	G/A	T/C	T/T	G/G	T/T	T/T	G/G	T/T	T/C	G/A	T/T	G/G

Sample number	rs10064525	rs27072	rs1042098	rs6347	rs10770140	rs10770141	rs3842727	rs6356	rs11575553	rs12666409	rs129882	rs129915	rs1611114	rs5320
793	T/T	C/C	A/A	T/T	T/C	G/A	T/T	C/C	G/G	T/T	C/C	G/A	T/C	G/G
794	T/T	C/C	A/A	T/T	T/T	G/G	T/T	T/C	G/G	T/T	C/C	G/A	T/C	G/G
795	T/T	C/C	G/A	T/C	T/C	G/A	T/T	T/C	G/G	T/T	C/C	G/A	T/C	G/G
796	T/T	C/C	A/A	T/T	T/T	G/G	T/T	T/T	G/A	T/T	T/T	A/A	T/C	G/A
797	T/T	C/C	A/A	T/T	T/T	G/G	T/T	T/T	G/G	T/T	T/T	A/A	T/T	G/G
798	T/T	C/C	G/G	T/C	T/T	G/G	T/T	T/T	G/G	T/A	T/C	G/A	T/C	G/G
799	T/T	C/C	A/A	T/C	T/T	G/G	T/T	T/T	G/A	T/T	T/C	A/A	C/C	G/G
800	T/T	C/C	A/A	T/T	T/T	G/G	T/T	T/T	G/G	T/A	C/C	G/G	T/T	G/G
801	T/T	C/C	G/A	T/C	T/T	G/G	T/T	T/T	G/G	T/T	T/C	G/A	T/T	G/G
